# Formation reaction mechanism and infrared spectra of *anti*-*trans*-methacrolein oxide and its associated precursor and adduct radicals

**DOI:** 10.1038/s42004-022-00644-0

**Published:** 2022-03-04

**Authors:** Jia-Rong Cai, Jung-Hsuan Su, Yuan-Pern Lee

**Affiliations:** 1 Department of Applied Chemistry and Institute of Molecular Science, National Yang Ming Chiao Tung University, Hsinchu, 300093 Taiwan; 2 Center for Emergent Functional Matter Science, National Yang Ming Chiao Tung University, Hsinchu, 300093 Taiwan; 3 Institute of Atomic and Molecular Sciences, Academia Sinica, Taipei, 106319 Taiwan

**Keywords:** Infrared spectroscopy, Atmospheric chemistry, Reaction kinetics and dynamics

## Abstract

Methacrolein oxide (MACRO) is an important carbonyl oxide produced in ozonolysis of isoprene, the most abundantly-emitted non-methane hydrocarbon in the atmosphere. We employed a step-scan Fourier-transform infrared spectrometer to investigate the source reaction of MACRO in laboratories. Upon UV irradiation of precursor CH_2_IC(CH_3_)CHI **(1)**, the CH_2_C(CH_3_)CHI radical **(2)** was detected, confirming the fission of the allylic C‒I bond rather than the vinylic C‒I bond. Upon UV irradiation of **(1)** and O_2_ near 21 Torr, *anti-trans*-MACRO **(3a)** was observed to have an intense OO-stretching band near 917 cm^−1^, much greater than those of *syn*-CH_3_CHOO and (CH_3_)_2_COO, supporting a stronger O‒O bond in MACRO because of resonance stabilization. At increased pressure (86‒346 Torr), both reaction adducts CH_2_C(CH_3_)CHIOO **(4)** and (CHI)C(CH_3_)CH_2_OO **(5)** radicals were observed, indicating that O_2_ can add to either carbon of the delocalized propenyl radical moiety of **(2)**. The yield of MACRO is significantly smaller than other carbonyl oxides.

## Abstract (web summary)

Methacrolein oxide (MACRO) is an important carbonyl oxide produced in the atmospheric ozonolysis of isoprene, but its formation is still not fully described. Here, the authors characterize the source reaction of MACRO via infrared spectroscopy and report spectra of *anti-trans*-MACRO as well as its associated precursor and adduct radicals.

## Introduction

Isoprene [2-methyl-1,3-butadiene, CH_2_ = CH–C(CH_3_) = CH_2_] is the most abundantly emitted non-methane volatile organic compound (VOC) emitted into Earth’s atmosphere; an emission budget ~530 Tg year^−1^, ~70% of the total biogenic VOC emission, was estimated^1,2^. Ozone is responsible for the removal of ~10% of isoprene^3,4^. The ozonolysis of isoprene produces three carbonyl oxides (so-called Criegee intermediates); formaldehyde oxide (CH_2_OO), methyl vinyl ketone oxide [MVKO, C_2_H_3_C(CH_3_)OO], and methacrolein oxide [MACRO, CH_2_C(CH_3_)CHOO] are produced with estimated branching ratios 58, 23, and 19%, respectively^3,5,6^.

Previously, detecting carbonyl oxides from ozonolysis of alkenes in laboratories was difficult because these source reactions are slow but the carbonyl oxides thus produced are highly reactive. Welz et al. reported an original reaction scheme to generate the simplest carbonyl oxide CH_2_OO in laboratories from the reaction of CH_2_I with O_2_ on photolysis of CH_2_I_2_ in O_2_ with ultraviolet (UV) light^7^. Further extension of this scheme to produce substituted carbonyl oxides has promoted active research, as discussed in several reviews^8–14^.

**Fig. 1:**
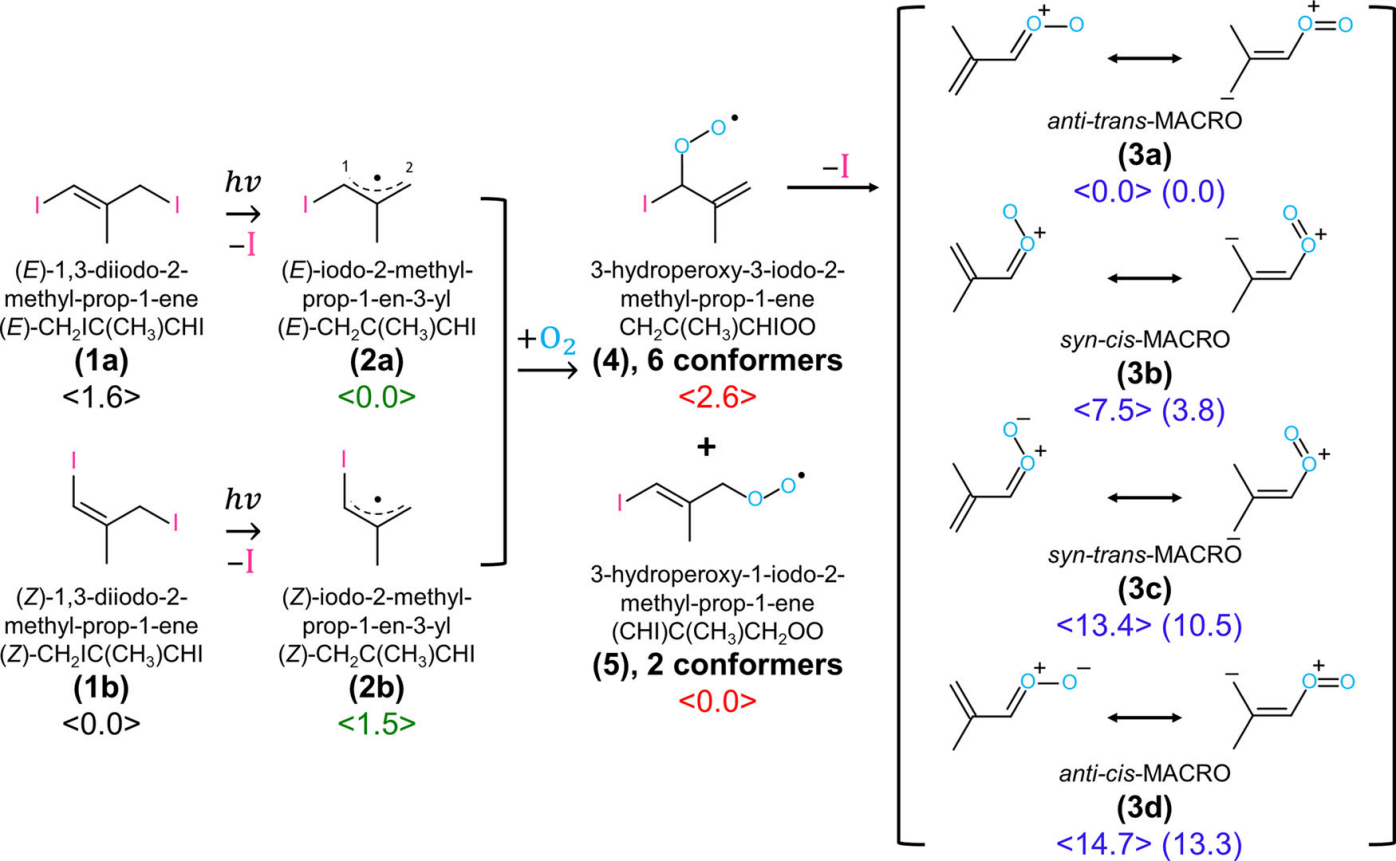
Reaction scheme of photolysis of 1,3-diiodo-2-methyl-prop-1-ene (1) to produce methacrolein oxide (MACRO, 3). Two conformers of 1,3-diiodo-2-methyl-prop-1-ene [CH_2_IC(CH_3_)CHI] **(1)** and iodo-2-methyl-prop-1-en-3-yl [CH_2_C(CH_3_)CHI] **(2)**, and four conformers of MACRO [CH_2_C(CH_3_)CHOO] **(3)** are depicted. Iodoperoxy adducts 3-hydroperoxy-3-iodo-2-methyl-prop-1-ene **(4)**, CH_2_C(CH_3_)CHIOO, and 3-hydroperoxy-1-iodo-2-methyl-prop-1-ene **(5)**, (CHI)C(CH_3_)CH_2_OO, have 6 and 2 conformers (not shown), respectively. The major resonance structures of MACRO are also shown. The relative energies (in kJ mol^–1^), computed with the B3LYP/aug-cc-pVTZ-pp method, are shown in brackets for conformers of each species; those of CCSD(T)-F12/CBS(TZ-F12,QZ-F12)//B2PLYP-D3/cc-pVTZ, reported by Vansco et al.^15^ are listed in parentheses.

To produce MACRO, CH_2_C(CH_3_)CHOO, following this method from photolysis of CH_2_C(CH_3_)CHI_2_ in O_2_ is, however, difficult because this precursor is extremely unstable. Vansco et al. reported a unique method to produce MACRO on photolysis at 248 nm of a gaseous mixture of 1,3-diiodo-2-methyl-prop-1-ene [CH_2_IC(CH_3_)CHI] **(1)** and O_2_ that was pulsed into a quartz capillary reactor tube^15^. These authors assumed that photolysis of CH_2_IC(CH_3_)CHI **(1)** at 248 nm resulted in a preferential dissociation of the allylic, rather than the vinylic, C–I bond, to form the iodoalkenyl radical 3-iodo-2-methyl-prop-1-en-3-yl [CH_2_C(CH_3_)CHI] **(2)**. Subsequent addition of O_2_ with this resonance-stabilized radical **(2)** to form adduct 3-hydroperoxy-3-iodo-2-methyl-prop-1-ene CH_2_C(CH_3_)CHIOO **(4)** that readily breaks the remaining C–I bond to produce the carbonyl oxide MACRO **(3)**. A detailed reaction scheme appears in Fig. 1; the chemical formula and labels of key species are also presented. Four conformers of MACRO, *anti*-*trans*, *syn*-*cis*, *syn*-*trans*, and *anti*-*cis* (with increasing energy, shown in Fig. 1) are predicted to exist; *syn*- and *anti*- indicate the orientation of the CH_2_ = C(CH_3_) moiety relative to the terminal oxygen atom, and *cis*- and *trans*- indicate the relative orientation of the C = C bond and the C = O bond. The UV-visible spectrum of jet-cooled MACRO was obtained by means of UV-visible depletion of the parent ion signal of MACRO at *m*/*e* = 86 upon photoionization at 10.5 eV^15^, but this spectrum with maximum absorption near 380 nm provides no specific information on the conformation of MACRO; equal populations of the conformers were assumed because these four conformers were predicted to have energies within 13.3 kJ mol^−1^ according to the CCSD(T)-F12/CBS(TZ-F12,QZ-F12)//B2PLYP-D3/cc-pVTZ method^15^. Lin et al. reported the direct UV-visible absorption spectrum of MACRO with a maximum at 397 nm upon photoirradiation of the same precursors at 248 nm and 298 K^16^. Unlike MVKO^17^, the near-infrared action spectra of MACRO could not be obtained by probing OH radicals because OH is not a significant reaction product. The mechanism for the formation of MACRO, including the characterization of the associated iodoalkenyl radical **(2)** before its reaction with O_2_ and the iodoperoxy radical adducts **(4)** and **(5)** before the fission of the second C–I bond, has not been identified. The mid-infrared spectrum of MACRO and other related intermediates will provide a clue to the conformation of these species and a detailed mechanism for the production of MACRO from UV photolysis of **(1)** in O_2_.

We have previously employed a unique step-scan Fourier-transform infrared (FTIR) absorption technique to detect unstable species^18^. The wide spectral and temporal coverage enables us to monitor several reaction intermediates simultaneously; their temporal behavior provides valuable information to understand the detailed reaction mechanism. With this technique, we have successfully detected infrared spectra of carbonyl oxides CH_2_OO^19,20^, CH_3_CHOO^21^, (CH_3_)_2_COO^22^, MVKO^23^, and the associated adduct CH_2_IOO^24^; we explored also the mechanism and the intermediates in reactions of CH_2_OO with CH_2_OO^25^, SO_2_^26^_,_ HC(O)OH^27^, and HCl^28^.

In this work, we extended our focus to MACRO and investigated the UV photodissociation of the precursor CH_2_IC(CH_3_)CHI **(1)** to observe the CH_2_C(CH_3_)CHI radical **(2)**, confirming that only the allylic C–I bond was broken. When oxygen at varied pressure was added, the IR spectra of the *anti*-*trans*-MACRO [CH_2_C(CH_3_)CHOO] **(3a)** and the adducts CH_2_C(CH_3_)CHIOO **(4)** and (CHI)C(CH_3_)CH_2_OO **(5)** radicals were characterized. The IR spectrum of MACRO indicates that the preferred conformation is *anti-trans* and provides direct spectral evidence for resonance stabilization and hyper-conjugation of MACRO.

## Results and discussion

### Quantum-chemical calculations

Although Vansco et al.^15^ has reported high-level calculations for conformers of MACRO, we performed calculations at the B3LYP/aug-cc-pVTZ level of theory mainly for predictions of vibrational wavenumbers and IR intensities of various conformers of MACRO and other associated species. As summarized in Supplementary Note 1, the optimized geometries and Cartesian coordinates of conformers of precursors **(1)**, iodoalkenyl radicals CH_2_C(CH_3_)CHI **(2)** and CH_2_IC(CH_3_)CH **(6)**, carbonyl oxides MACRO **(3)**, a possible cyclic peroxide product dioxole from unimolecular isomerization of MACRO^29,30^, and iodoperoxy radical adducts **(4)** and **(5)** are presented in Supplementary Figs. 1–4 and Supplementary Tables 1–4. Relative energies of conformers are also listed in these figures and those of MACRO are compared with high-level calculations by Vansco et al.^15^.

Computed scaled harmonic vibrational wavenumbers and IR intensities of these species are listed in Supplementary Tables 5–9, respectively. The harmonic vibrational wavenumbers of all species discussed in this paper were scaled according to the equation *y* = 0.9683 *x* + 11.5, in which *y* and *x* are scaled and harmonic vibrational wavenumbers, respectively; this equation was derived on fitting the observed bands of precursor **(1a)** with computed harmonic vibrational wavenumbers. The average absolute deviation of scaled harmonic vibrational wavenumbers of **(1a)** from observed wavenumbers is 7.5 ± 6.4 cm^−1^; the error represents one standard deviation in the fitting. The computed anharmonic vibrational wavenumbers of the four conformers of MACRO, **(3a)**−**(3d)**, and dioxole are also listed in Supplementary Table S7. The rotational parameters for each vibrational state of **(3a)**−**(3d)** are listed in Supplementary Table S10.

### IR spectra of the iodoalkenyl radical CH_2_C(CH_3_)CHI (2)

**Fig. 2:**
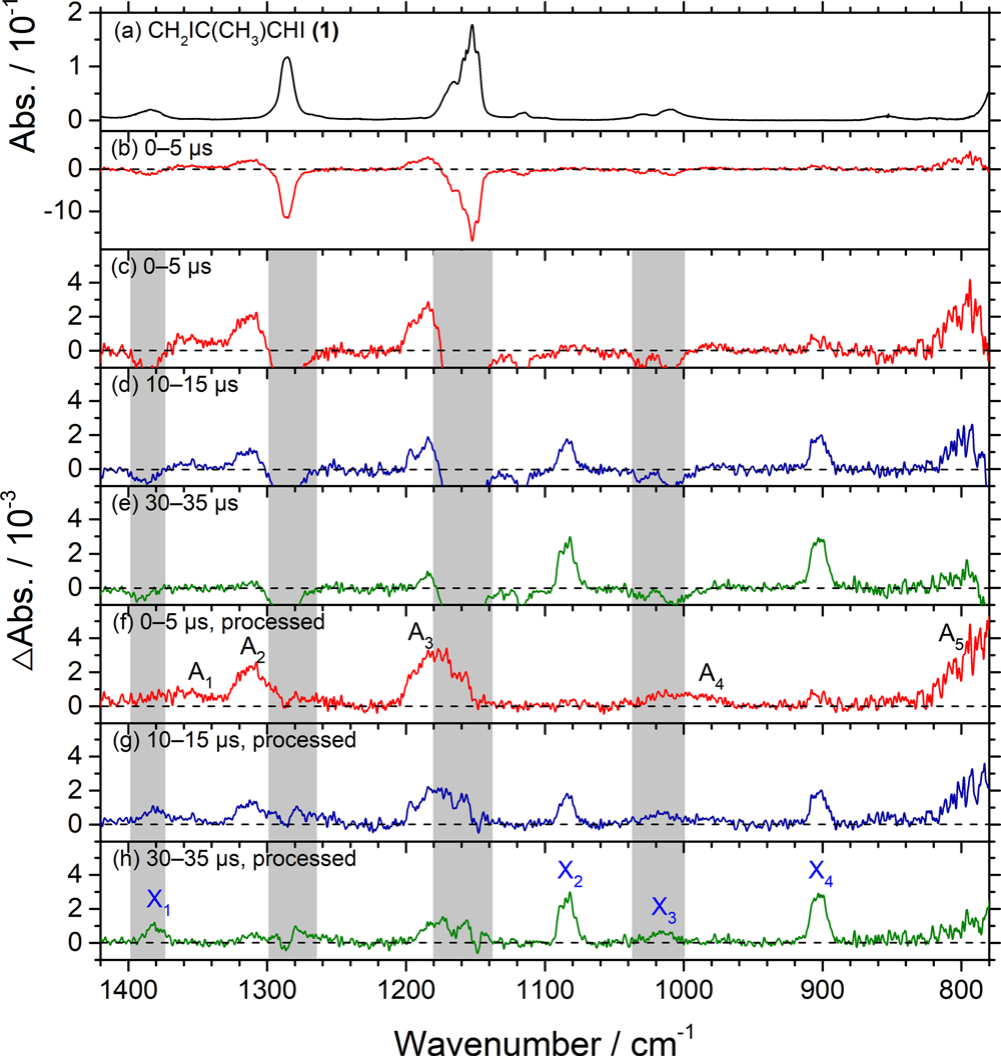
Observed and processed spectra in region 1420–780 cm^–1^ upon photolysis at 248 nm of a flowing mixture of CH_2_IC(CH_3_)CHI (1)/N_2_ (0.030/20.0 Torr). **a** Absorption spectrum before photolysis. **b** Difference spectra recorded 0–5 µs after photolysis. **c**–**e** Expanded difference spectra recorded 0–5, 10–15, and 30–35 µs after irradiation; negative bands are truncated. **f**–**h** Processed spectra of **c**‒**e** with absorption bands of precursor **(1)**, spectrum **a**, added back to eliminate negative bands. Gray areas represent regions of possible interference from absorption of the parent molecule. New features with decreasing intensity are marked A_1_–A_5_ in **f**. New features with increasing intensity are marked X_1_–X_4_ in **h**. The spectral resolution is 1.0 cm^–1^.

Precursor **(1)** is predicted to exist in (*Z*)- and (*E*)-conformations; the (*E*)-conformer **(1a)** is predicted to have energy 1.6 kJ mol^−1^ greater than the (*Z*)-conformer **(1b)** at the B3LYP/aug-cc-pVTZ level of theory. The IR spectra of **(1a)** and **(1b)** are shown in Supplementary Fig. 5 and their wavenumbers are listed in Supplementary Table 5. These spectra and results on photolysis (Fig. 2, same as Supplementary Fig. 6) are discussed in Supplementary Note 2. When precursor **(1)**, a mixture of **(1a)** and **(1b)** with spectrum shown in Fig. 2a, was irradiated with light at 248 nm, the intensity of its bands decreased significantly and weak new features appeared (Fig. 2b). Processing these difference spectra (Fig. 2c–e) by adding back the loss of the precursor yielded Fig. 2f–h, as discussed in Supplementary Note 2; the regions in which the absorption of the precursor might interfere are marked with gray rectangles. Three features near 1312, 1178, and 778 cm^−1^ with greater intensities and two weak ones near 1364 and 1002 cm^−1^ appeared upon UV irradiation and decreased in intensity with reaction period following nearly the same proportions; these transient features in group A, marked as A_1_‒A_5_ in Fig. 2f, are associated with the primary photolysis product. Four features near 1380, 1084, 1014, and 902 cm^−1^ increased in intensity continuously; these features in group X, marked as X_1_–X_4_ in Fig. 2h, are associated with the final product.

**Fig. 3:**
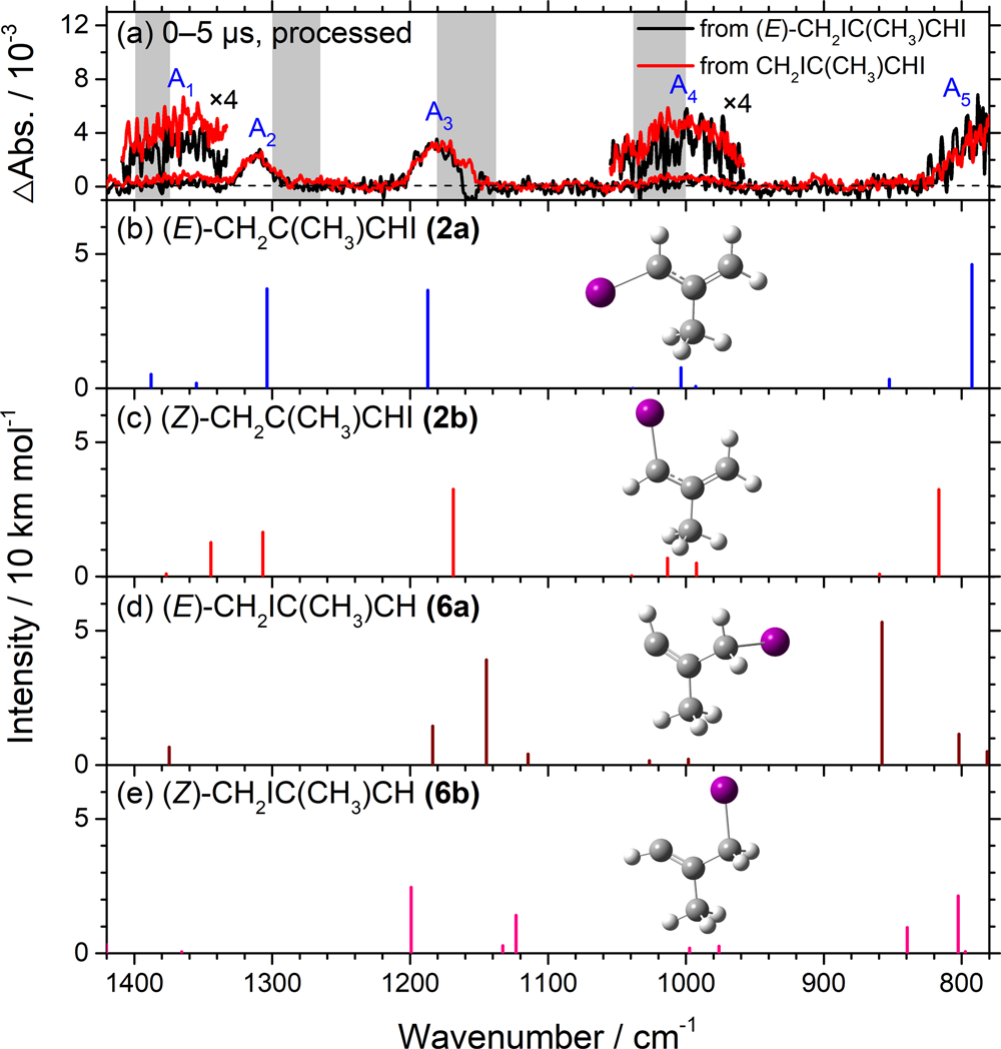
Comparison of bands in group A with IR stick spectra of four isomers of iodoalkenyl radicals. **a** Spectra of bands in group A recorded 0–5 μs after photolysis of (*E*)-CH_2_IC(CH_3_)CHI **(1a)** (black) and of a ~1:1 mixture of (*E*)- and (*Z*)-CH_2_IC(CH_3_)CHI **(1)** (red, taken from Fig. 2f; bands are labeled A_1_–A_5_. Weak bands A_1_ and A_4_ are expanded and shifted for clarity. Gray areas represent regions of possible interference from absorption of the parent molecule. IR stick spectra predicted for **b** (*E*)-CH_2_C(CH_3_)CHI **(2a)**, **c** (*Z*)-CH_2_C(CH_3_)CHI **(2b)**, **d** (*E*)-CH_2_IC(CH_3_)CH **(6a)**, and **e** (*Z*)-CH_2_IC(CH_3_)CH **(6b)** according to scaled harmonic vibrational wavenumbers and IR intensities predicted with the B3LYP/aug-cc-pVTZ-pp method are shown.

The spectrum consisting of bands in group A is reproduced in Fig. 3a as the red trace. A corresponding spectrum observed in experiments with (*E*)-CH_2_IC(CH_3_)CHI **(1a)** is shown as a black trace in Fig. 3a; these two traces are similar except that the red trace has a better signal-to-noise ratio (SNR). The stick IR spectra of both conformers of two possible photolysis products, (*E*)- and (*Z*)-CH_2_C(CH_3_)CHI, **(2a)** and **(2b)**, and (*E*)- and (*Z*)-CH_2_IC(CH_3_)CH, **(6a)** and **(6b)**, according to the scaled harmonic vibrational wavenumbers and intensities predicted with the B3LYP method are shown in Fig. 3b–e. The observed new features near 1364, 1312, 1178, 1002, and 778 cm^−1^ agree best, in terms of wavenumbers and relative intensities, with lines predicted near 1355, 1304, 1187, 1004, and 793 cm^−1^ for **(2a)**, the conformer with the least energy; a small contribution of **(2b)** cannot be positively excluded because of the similarity in predicted spectra. A comparison of observed bands with calculations in this spectral region is presented in Table 1. In contrast, the observed features in group A agree poorly with the spectra predicted for **(6a)** and **(6b)**. This observation of bands of **(2)** confirms that irradiation of **(1)** at 248 nm caused the breaking of the allylic C‒I bond rather than the vinylic C–I bond. Furthermore, because the spectra observed after photolysis of **(1a)** and **(1)** are similar, we deduced that the conformation of **(2)** was scrambled upon UV photolysis of **(1)**; the barrier to convert **(2b)** to **(2a)** is ~50 kJ mol^−1^, much smaller than the excess energy ~300 kJ mol^−1^ at 248-nm photolysis. The calculated energy difference, ~1.5 kJ mol^−1^, between **(2a)** and **(2b)** implies a population ratio 65:35 according to the Boltzmann distribution at 298 K, but the observed spectra seem to show a contribution of **(2b)** smaller than the predicted ratio if one considers that a doublet of similar intensity was predicted for the (*Z*)-conformer in region 1300–1350 cm^−1^ (Fig. 3c), but only one significant feature was observed. We are, however, uncertain about this population ratio because the predicted IR intensities might have large errors.

**Table 1:** Comparison of observed vibrational wavenumbers and IR intensities of (*E*)-CH_2_C(CH_3_)CHI (**2a**) in region 750–1500 cm^−1^ with those calculated with the B3LYP/aug-cc-pVTZ-pp method.

Mode^a^	Sym.	Experiment		Calculation		Mode description^e^
		*ν*/cm^−1^	Intensity^b^	*ν*/cm^−1c^	Intensity^d^	
*v*_8_	a′			1458	21.2	CH_3_ def.
*v*_9_	a′′			1448	11.1	CH_3_ def.
*v*_10_	a′			1388	5.2	CH_3_ umbrella
*v*_11_	a′	1364	28	1355	2.0	C^(1)^C^(3)^ str.
*v*_12_	a′	1312	43	1304	37.1	C^(1)^C^(2)^C^(4)^ asym. str.
*v*_13_	a′	1178	81	1187	36.5	CHI bend
*v*_14_	a′′			1039	0.0	C^(3)^H^(3)^H^(4)^ twist
*v*_15_	a′	1002	33	1004	7.7	C^(3)^H^(3)^H^(4)^ wag
*v*_16_	a′			993	0.8	CH_2_ *ip* bend
*v*_17_	a′			853	3.4	C^(1)^C^(3)^ str./C^(1)^C^(2)^C^(4)^ bend
*v*_18_	a′′	778	100	793	46.1	CH_2_ wag

^a^Mode numbers are ordered without consideration of symmetry to conform with those of (*Z*)-CH_2_C(CH_3_)CHI **(2b)**.

^b^Percentage of IR intensity relative to the most intense band near 778 cm^−1^.

^c^Harmonic vibrational wavenumber *x* scaled according to 0.9683 *x* + 11.5; see text.

^d^In unit km mol^−1^.

^e^Approximate mode description. asym.: *anti*-symmetric; str.: stretch; def.: deform; *ip*: in-plane.

**Fig. 4:**
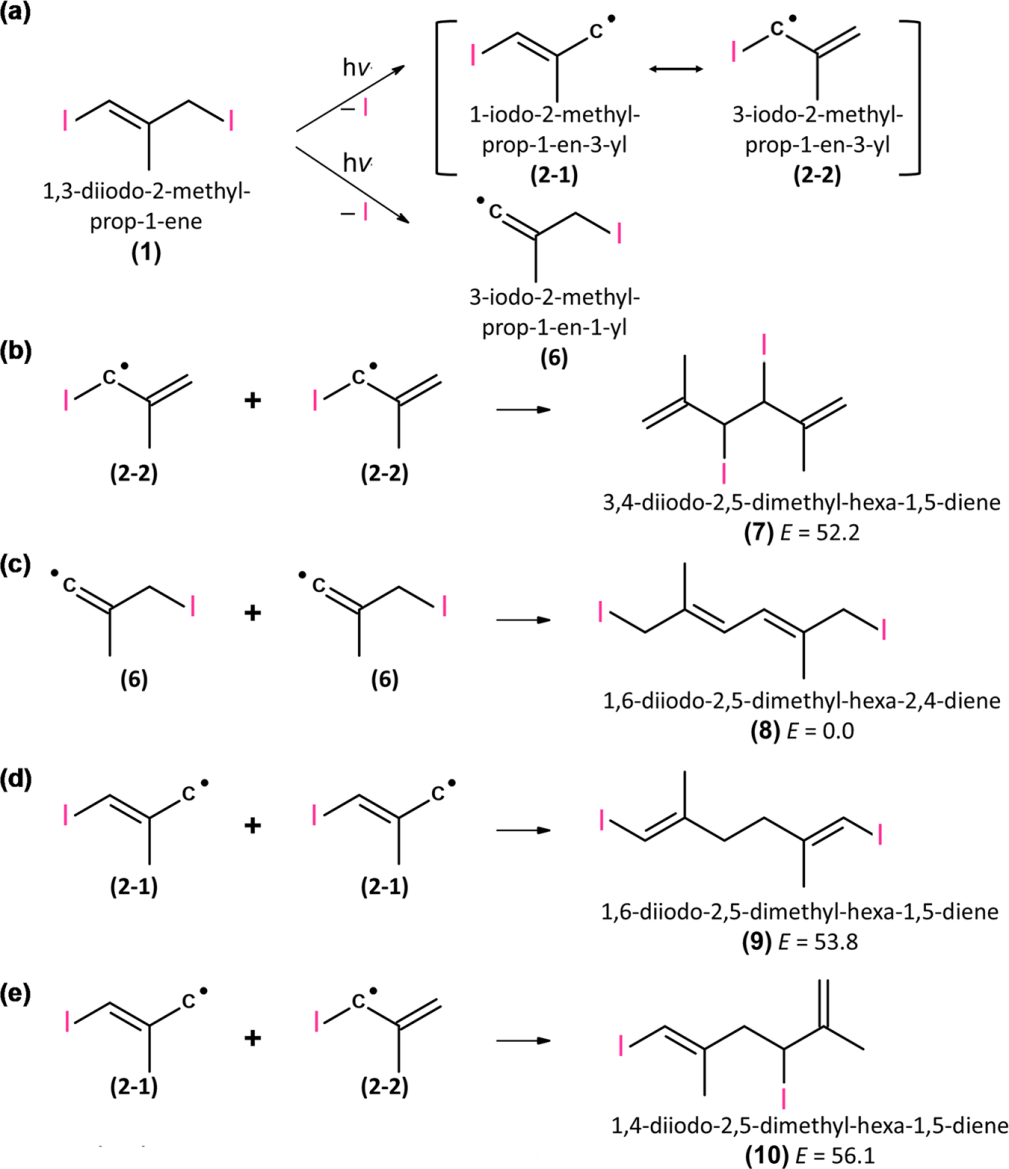
Possible dimeric products produced on photolysis of 1,3-diiodo-2-methyl-prop-1-ene (1). **a** Mechanism of photolysis of 1,3-diiodo-2-methyl-prop-1-ene **(1)** to produce 1-iodo-2-methyl- prop-1-en-3-yl **(2-1)**, 3-iodo-2-methyl-prop-1-en-3-yl **(2-2)**, and 3-iodo-2-methyl-prop-1-en-1-yl **(6)**. **b**–**e** Possible dimerization reactions among **(2-1)**, **(2-2)**, and **(6)**; the relative energies (kJ mol^–1^) of **(7)**−**(10)** are computed with the B3LYP/aug-cc-pVTZ-pp method.

**Fig. 5:**
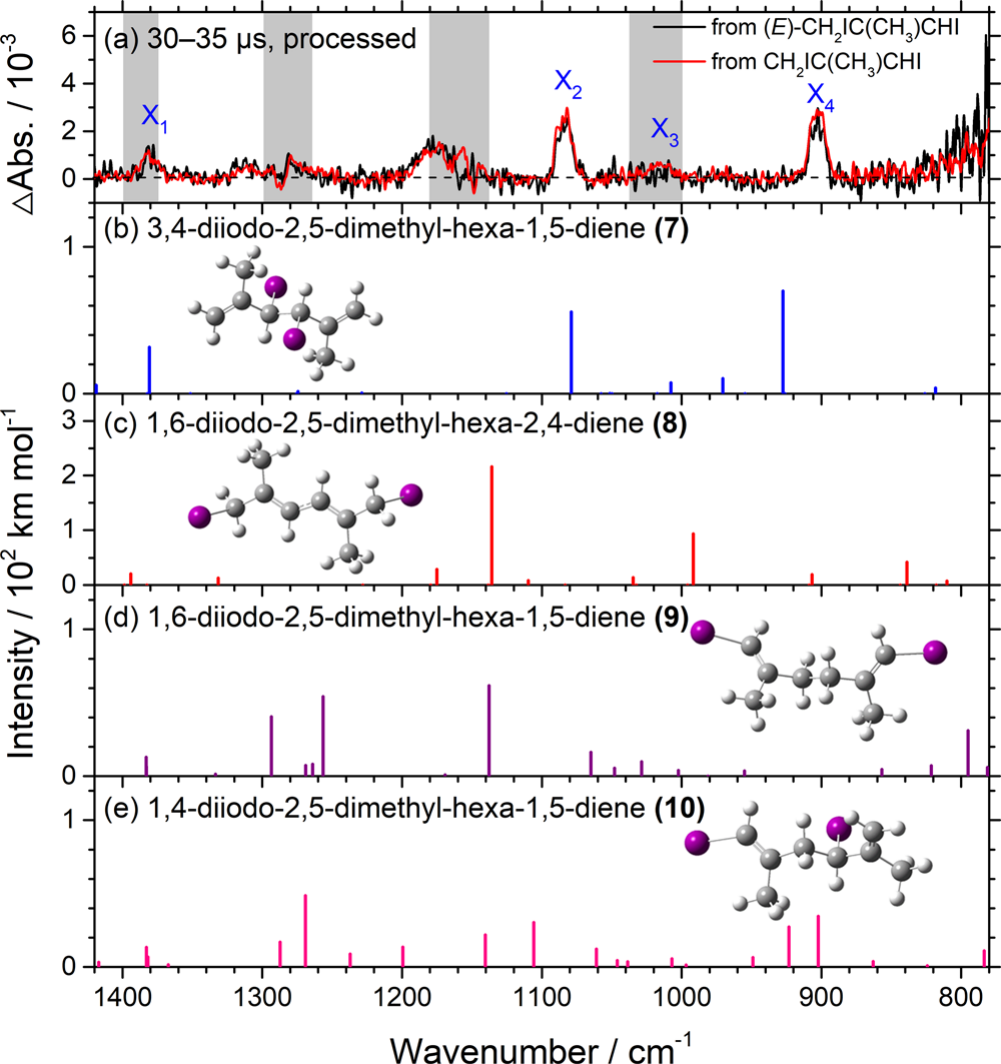
Comparison of IR spectra of end products (group X) on photolysis of CH_2_IC(CH_3_)CHI (1)/N_2_ with predicted IR stick spectra of possible products. **a** Spectrum of bands in group X recorded 30–35 μs after photolysis of (*E*)-CH_2_IC(CH_3_)CHI (black) or a mixture of (*E*)-and (*Z*)-CH_2_IC(CH_3_)CHI (red); taken from Fig. 2h; the resolution is 1.0 cm^–1^. Gray areas represent regions of possible interference from absorption of the parent molecule. IR stick spectra according to scaled harmonic vibrational wavenumbers and IR intensities predicted with the B3LYP/aug-cc-pVTZ-pp method are shown for four possible dimers: **b** 3,4-diiodo-2,5- dimethyl-hexa-1,5-diene **(7)**, **c** 1,6-diiodo-2,5-dimethyl-hexa-2,4-diene **(8)**, **d** 1,6-diiodo- 2,5-dimethyl-hexa-1,5-diene **(9)**, and **e** 1,4-diiodo-2,5-dimethyl-hexa-1,5-diene **(10)**; the structures are shown in Fig. 4.

The product of dimerization of iodo-radicals **(2)** was also observed at a later period. Because of resonance, two forms 1-iodo-2-methyl-prop-1-en-3-yl **(2-1)** and 3-iodo-2-methyl-prop-1-en-3-yl **(2-2)** might exist for **(2)**, as shown in Fig. 4a for the *E*-conformer as an example. Possible secondary reactions are shown in Fig. 4b–e, with diene products **(7)**−**(10)**. The observed spectrum of the end product, group X, is reproduced in Fig. 5a and compared with predicted spectra of **(7)**−**(10)** in Fig. 5b–e. Observed bands in group X near 1380, 1084, 1014, and 902 cm^−1^ agree with scaled harmonic vibrational wavenumbers and relative intensities predicted near 1381, 1079, 1008, and 927 cm^−1^ for 3,4-diiodo-2,5-dimethyl-hexa-1,5-diene **(7)**, produced from dimerization of **(2-2)**, shown in Fig. 4b. The observation of end product **(7)** further supports that only the allylic C‒I bond rather than the vinylic C–I bond was broken upon irradiation at 248 nm, and that the radical thus produced, **(2)**, exists in 3-iodo-2-methyl-prop-1-en-3-yl **(2-2)** as its major form (in which C1–C4 has a double bond character), consistent with the predicted bond length of C1–C4 (1.380 Å) slightly smaller than that of C1–C2 (1.391 Å) for **(2a)**; C1 is the central carbon atom and C2 is the carbon with the I atom, as labeled in Supplementary Fig. 1c.

### IR spectrum of carbonyl oxide *anti-trans*-MACRO (3a)

**Fig. 6:**
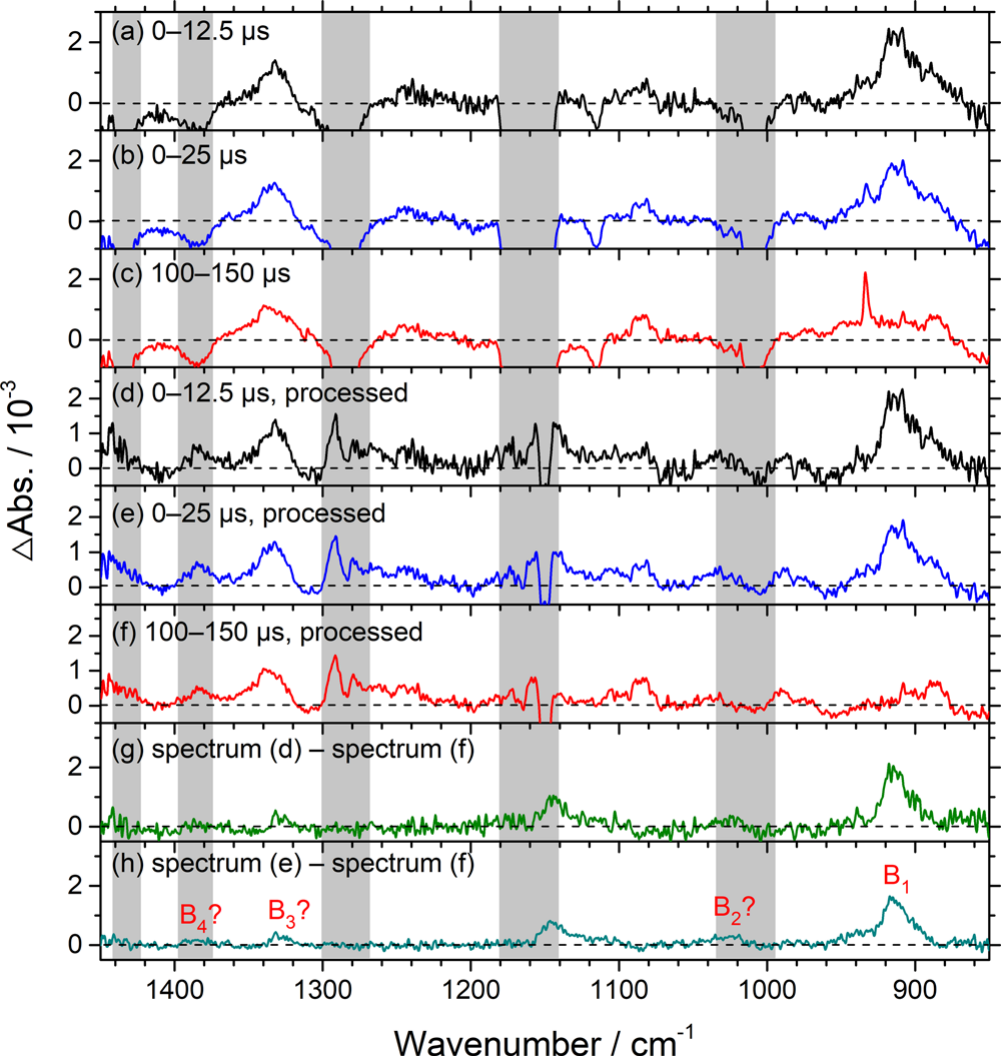
Observed and processed spectra in region 1450–850 cm^–1^ upon photolysis at 248 nm of a flowing mixture of CH_2_IC(CH_3_)CHI (1)/O_2_ (0.030/20.0 Torr). Difference spectra recorded 0–12.5 (**a**), 0–25 (**b**), and 100–150 (**c**) µs after irradiation; negative bands are truncated. **d**–**f** Processed spectra of **a**–**c** with bands of CH_2_C(CH_3_)CHI **(2)** and methacrolein (MACR) removed and those of the precursor **(1)** added back. Gray areas represent regions of possible interference from absorption of the parent molecules. **g** Spectrum **d** subtracts spectrum **f**. **h** Spectrum **e** subtracts spectrum **f**. New features are marked B_1_–B_4_ in **h**; the latter three are uncertain because of their small intensities. The spectral resolution is 1.0 cm^–1^.

Results and data processing of photolysis of flowing mixtures of **(1)** (30 mTorr) and O_2_ (20.0–21.0 Torr), recorded with external and internal ADC (with improved SNR), are presented in Supplementary Figs. 7 and 8 (reproduced as Fig. 6), respectively, and discussed in detail in Supplementary Note 3. Processing observed difference spectra recorded with an internal ADC (Fig. 6a–c) by removing contributions of **(2)** and end product methacrolein and adding back the loss of the precursor yielded Fig. 6d–f; the regions in which the absorption of the precursor might interfere are marked with gray rectangles. Further processing by taking out contributions of other stable products (Fig. 6f) produced cleaner spectra, as shown in Fig. 6g, h. Only one prominent band near 917 cm^−1^ showed a transient nature and attained its maximum 5‒10 µs after irradiation; we mark this as B_1_ in Fig. 6h. Weak features B_2_–B_4_ near 1025, 1332, and 1386 cm^−1^ might also belong to this group; as we are uncertain about these bands because of their small intensities and possible interference from absorption of the parent or the stable products, we indicate them with ? marks.

**Fig. 7:**
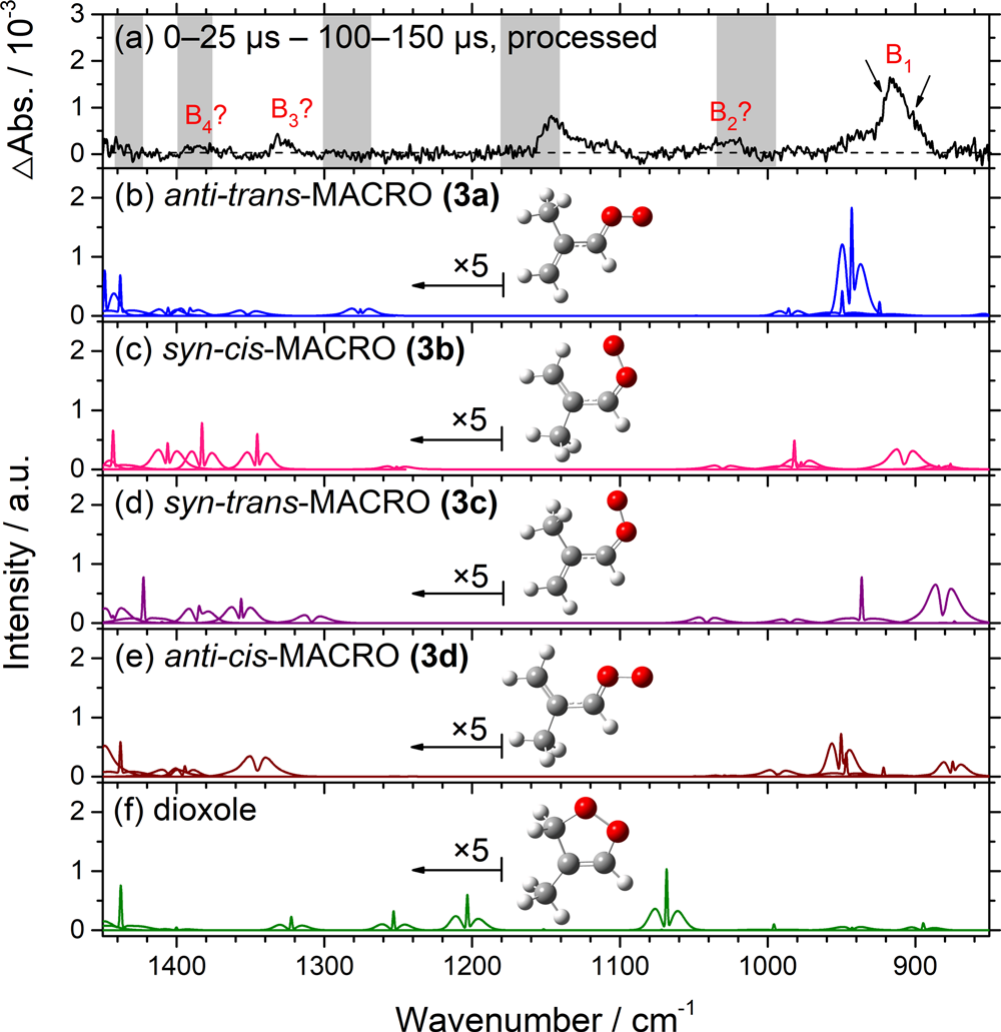
Comparison of bands in group B with simulated spectra of various conformers of carbonyl oxide MACRO and dioxole. **a** Spectrum of bands in group B after photolysis; taken from Fig. 6h. The spectral resolution is 1.0 cm^–1^. Gray areas represent regions of possible interference from absorption of the parent molecule. IR spectra simulated with PGOPHER (*J*_max_ = 150, *T* = 298 K, fwhm = 1.28 cm^–1^) are shown for **b** *anti-trans*-MACRO **(3a)**, **c** *syn-cis*-MACRO **(3b)**, **d** *syn-trans*-MACRO **(3c)**, **e** *anti-cis*-MACRO **(3d)**, and **f** dioxole. Band intensities are multiplied by 5 in region 1180–1450 cm^–1^.

The photolysis of a mixture of precursor **(1)** and O_2_ at 248 nm is expected to produce carbonyl oxide MACRO **(3)**, as reported by Vansco et al.^15^. The spectrum of group B in Fig. 6h is reproduced in Fig. 7a to compare with the simulated spectra of four conformers of the carbonyl oxides MACRO: *anti*-*trans*-, *syn*-*cis*-, *syn*-*trans*-, and *anti*-*cis*-CH_2_C(CH_3_)CHOO, **(3a)**−**(3d)**, in Fig. 7b–e. We employed anharmonic vibrational wavenumbers (Supplementary Table 7) and rotational parameters (Supplementary Table 10) predicted with the B3LYP method and simulated the rotational contours with the PGOPHER program (Supplementary Figs. 9 and 10)^31^, as described in detail in the Supplementary Note 4. The spectrum of a possible product dioxole simulated according to anharmonic vibrational calculations is also shown in Fig. 7f for comparison.

Observed intense band B_1_ near 917 cm^−1^ matches best with the spectrum simulated for *anti-trans*-MACRO **(3a)** in terms of vibrational wavenumbers and relative intensities. The OO- stretching (ν_15_) band of **(3a)** was predicted at 944 cm^−1^ and to have the largest IR intensity (201 km mol^−1^); other bands in region 850–1450 cm^−1^ have IR intensities less than 26 km mol^−1^. For the four conformers, only **(3a)** was predicted to have a single intense band in this region; others were predicted to have more than two bands with comparable intensities. Furthermore, two *c*-type bands were predicted for modes ν_24_ and ν_25_ of **(3a)** near 950 and 924 cm^−1^; they might correspond to the small narrow features near 921 and 902 cm^−1^, indicated with arrows in Fig. 7a; other conformers were predicted to have only one *c*-type band near 900 cm^−1^. A comparison of observed bands with predicted vibrational wavenumbers and intensities of **(3a)** is listed in Table 2. The observed band near 917 cm^−1^ appears to have an insignificant Q-branch and a broader rotational contour as compared with that simulated with the PGOPHER program, presumably because of the contribution of hot bands from excited states of the low-energy vibrational modes. Similar broadening was observed for carbonyl oxides containing a methyl rotor, CH_3_CHOO^21^, (CH_3_)_2_COO^22^, and MVKO^23^.

**Table 2:** Comparison of observed vibrational wavenumbers and IR intensities of *anti-trans*-CH_2_C(CH_3_)CHOO (3a) and *syn*-*cis*-CH_2_C(CH_3_)CHOO (3b) in region 840–1410 cm^−1^ with those calculated with the B3LYP/aug-cc-pVTZ method.

Mode	Sym.	Experiment		Harmonic		Anharmonic	Mode description^d^
		*ν*/cm^−1^	Int.^a^	*ν*/cm^−1b^	Int.^c^	ν/cm^−1^	
*anti-trans*-CH_2_C(CH_3_)CHOO **(3a)**							
* v*_9_	a′			1408	4.8	1406	CH_2_ bend
* v*_10_	a′			1391	4.5	1392	CH_3_ umbrella
* v*_11_	a′			1356	4.4	1352	C^(3)^C^(1)^C^(2)^ asym. str.
* v*_12_	a′			1273	5.8	1276	CH *ip* bend
* v*_13_	a′			1033	0.3	1034	C^(3)^H^(3)^H^(4)^ wag
* v*_14_	a′			983	16.7	985	CH_2_ rock
* v*_15_	a′	917	100	946	200.6	944	OO str.
* v*_16_	a′			848	7.4	848	C^(1)^C^(3)^ str.
* v*_23_	a”			1052	0.4	1048	C^(3)^H^(3)^H^(4)^ twist
* v*_24_	a”	921?	^e^	950	26.2	950	CH *oop* bend
* v*_25_	a”	902?	^e^	930	14.7	924	CH_2_ wag/CH *oop* bend
*syn*-*cis***-**CH_2_C(CH_3_)CHOO **(3b)**							
* v*_9_	a′			1396	15.0	1406	CH_3_ umbrella/ CH_2_ bend
* v*_10_	a′	1386?	50	1380	12.4	1383	CH_3_ umbrella/ CH_2_ bend
* v*_11_	a′	1332?	60	1350	12.4	1346	CH *ip* bend
* v*_12_	a′			1247	2.3	1252	C^(3)^C^(1)^C^(2)^ asym. str.
* v*_13_	a′	1025?	100	1022	15.5	1030	CH_2_ rock/ C^(3)^H^(3)^H^(4)^ wag
* v*_14_	a′			986	38.1	978	C^(3)^H^(3)^H^(4)^ wag
* v*_15_	a′	917?	^*e*^	910	81.0	907	OO str.
* v*_16_	a′			884	14.0	884	C^(1)^C^(3)^ str./OO str.
* v*_23_	a”			1056	0.4	1053	C^(3)^H^(3)^H^(4)^ twist
* v*_24_	a”			988	26.7	982	CH_2_ *oop* bend
* v*_25_	a”			893	5.2	876	CH *oop* bend

^a^Percentage of IR intensity relative to the most intense band near 917 cm^−1^.

^b^Harmonic vibrational wavenumber *x* scaled according to 0.9683 *x* + 11.5; see text.

^c^In unit km mol^−1^.

^d^Approximate mode description. sym.: symmetric; asym.: *anti*-symmetric; str.: stretch; def.: deform; *ip*: in-plane; *oop*: out-of-plane.

^e^Overlap with the band at 917 cm^−1^.

A small contribution of *syn*-*cis*-MACRO **(3b)**, the second least-energy conformer with energy 7.5 (3.8 from CCSD(T)-F12^15^) kJ mol^−1^ greater than *anti-trans*-MACRO **(3a)**, might be assigned to the observed weak bands B_2_–B_4_, but we are unable to confirm this definitively because of the small intensity and interference. The observed bands near 1025, 1332, and 1386 cm^−1^ agree satisfactorily with the more intense bands of **(3b)** predicted near 1030, 1346, and 1383 cm^−1^. The band predicted near 907 cm^−1^ might overlap with band B_1_ of **(3a);** two bands predicted near 980 cm^−1^ might suffer interference from the parent absorption, so they are unobserved.

The agreement of observed bands with those predicted for the *syn*-*trans*- and *anti-cis*-MACRO, **(3c)** and **(3d)**, is less satisfactory. Considering the computed relative energies of these two conformers, 10.5 (13.4) and 13.3 (14.7) kJ mol^−1^ above **(3a)**, our observation of predominant *anti-trans*-MACRO **(3a)** with a negligibly small contribution of **(3c)** and **(3d)** is reasonable; the listed energies are from CCSD(T)-F12^15^ and B3LYP (listed parenthetically). The observed spectrum agrees poorly with that predicted for dioxole (Fig. 7f).

### Resonance stabilization of MVKO and MACRO (3)

We compared the lengths of bonds O‒O and C‒O and the OO-stretching vibrational wavenumbers of **(3a)** with *syn*-*trans*-MVKO, *syn-*CH_3_CHOO, *anti-*CH_3_CHOO, and (CH_3_)_2_COO in Table 3 to confirm the resonance stabilization of MVKO and MACRO. The O‒O lengths 1.365 Å for **(3a)** and 1.353 Å for *syn*-*trans*-MVKO are significantly smaller than those of other carbonyl oxides (~1.380 Å). The observed OO-stretching vibrational wavenumbers 917 cm^−1^ for **(3a)** and 948 cm^−1^ for *syn*-*trans*-MVKO are much greater than the corresponding values 871‒887 cm^−1^ for other species. All this evidence supports that the COO moieties of MACRO and MVKO are resonance stabilized by the adjacent vinyl group so that the extended π-electron delocalization strengthens the O‒O bond significantly; the major resonance structures of MACRO and MVKO are shown in Supplementary Fig. 11a and b, respectively; the major resonance structures for four conformers of MACRO are also depicted in Fig. 1. Furthermore, the molecular orbitals of MACRO and MVKO also show delocalization of π-electron densities over the CCCOO skeleton, as shown in Supplementary Fig. 11c for node = 0–2; a more complete set of molecular orbitals have been reported by Vansco et al.^15^.

**Table 3:** Comparison of O–O and C–O bond lengths and OO-stretching vibrational wavenumbers of Criegee intermediates.

	*anti*-*trans*-MACRO	*syn*-*trans*-MVKO	*syn*-CH_3_CHOO	*anti*-CH_3_CHOO	(CH_3_)_2_COO
r(O‒O)/Å	1.365	1.353	1.380	1.381	1.380
r(C‒O)/Å	1.266	1.297	1.284	1.279	1.270
ν(OO)/cm^−1^	917	948	871	884	887
Calculation method	B3LYP/aug-cc-pVTZ	CCSD(T)/cc-pVTZ	NEVPT2(8,8)/aug-cc-pVDZ	NEVPT2(8,8)/aug-cc-pVDZ	B3LYP/aug-cc-pVTZ
Reference	This work	^22^	^26^	^26^	^27^

That the observed vibrational wavenumber 917 cm^−1^ for the OO-stretching mode of **(3a)** is smaller than a value, 948 cm^−1^, observed for MVKO can be explained with a concept of hyper-conjugation generally employed in organic chemistry; this resonance structure is also shown in Supplementary Fig. 11a. In this hyper-conjugation structure, the O–O bond has single-bond character, whereas the adjacent C=O bond has double-bond character; this contribution explains that MACRO has a longer O–O bond length and a shorter C–O length, with *R*_OO_ = 1.365 Å and *R*_CO_ = 1.266 Å for MACRO and *R*_OO_ = 1.353 Å and *R*_CO_ = 1.297 Å for MVKO. Our observation of the OO-stretching wavenumbers of carbonyl oxides provided a direct spectral confirmation of the resonance stabilization and hyper-conjugation of MACRO. Because of this resonance stabilization, a smaller reactivity of MACRO and MVKO was predicted and observed^16,32,33^.

### Assignments of iodoperoxy radical adducts

**Fig. 8:**
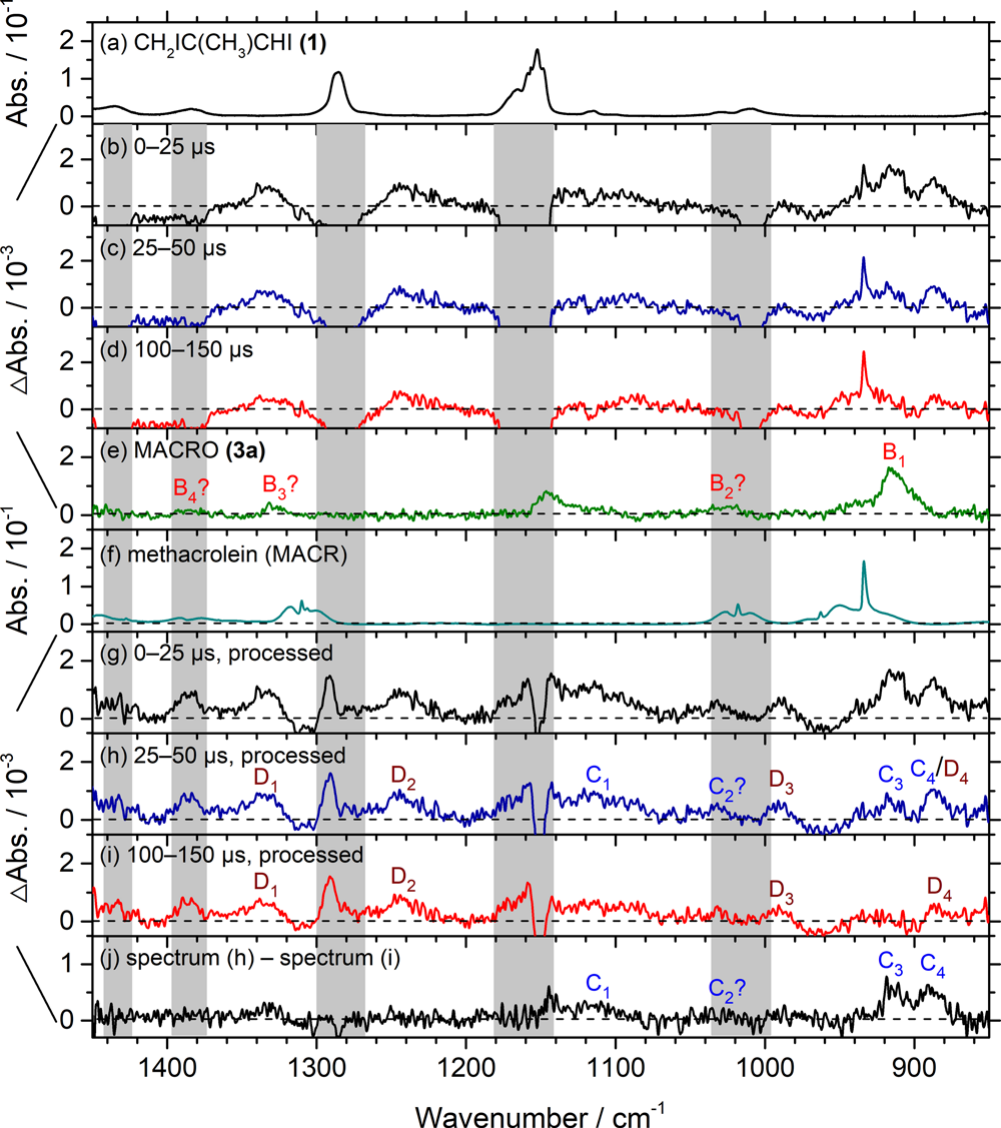
Observed and processed spectra in region 1450–850 cm^–1^ upon photolysis at 248 nm of a flowing mixture of CH_2_IC(CH_3_)CHI (1)/O_2_ (0.060/334 Torr). **a** Absorption spectrum before photolysis. Difference spectra recorded 0–25 (**b**), 25–50 (**c**), and 100–150 (**d**) µs after irradiation. **e** Spectrum of MACRO **(3)** taken from Fig. 6h. **f** Absorption spectrum of methacrolein (MACR). **g**–**i** Processed spectra of **b**–**d** with bands of CH_2_C(CH_3_)CHI **(2)** and MACR removed and those of the precursor **(1)** added back. **j** Spectrum **h** subtracts spectrum **i** to remove the contribution of bands in group D. Gray areas represent regions of possible interference from absorption of the parent molecules **(1)**. New features are marked C_1_–C_4_ and D_1_–D_4_ in **h**–**j**. The spectral resolution is 1.0 cm^–1^.

Results on photolysis of a flowing mixture of **(1)** (60 mTorr) and O_2_ (21–346 Torr) are discussed in detail in Supplementary Note 5. Figure 8 (same as Supplementary Fig. 12) shows the results of experiments on CH_2_IC(CH_3_)CHI **(1)**/O_2_ (0.060/334 Torr) in a flowing mixture. Details in processing observed difference spectra (Fig. 8b–d) by stripping bands of MACRO and methacrolein and adding back the decrease in precursor bands to obtain Fig. 8g–i are discussed in Supplementary Note 5; the regions in which the absorption of the precursor might interfere are marked with gray rectangles. Two sets of new bands were observed. Bands of group D near 1333, 1243, 990, and 886 cm^−1^, marked D_1_–D_4_ in Fig. 8i, are associated with a more stable intermediate; bands of group C near 1116, 1031, 914, and 888 cm^−1^, marked C_1_–C_4_ in Fig. 8j, are associated with a less stable intermediate, according to their temporal profiles.

For the simplest carbonyl oxide CH_2_OO, both CH_2_OO and the adduct ICH_2_OO were produced from UV photolysis of CH_2_I_2_ + O_2_; the yield of ICH_2_OO increased with pressure because the adduct was stabilized at high pressure^24^. Similarly, the adduct C_2_H_3_C(CH_3_)IOO was produced on photolysis of a mixture of the precursor of MVKO, (CH_2_I)HC = C(CH_3_)I, and O_2_ at 248 nm at high pressure^23^. As discussed previously, photolysis of precursor **(1)** produces only CH_2_C(CH_3_)CHI **(2)**, not CH_2_IC(CH_3_)CH **(6)**; possible structures of the adducts are hence CH_2_C(CH_3_)CHIOO **(4)** or (CHI)C(CH_3_)CH_2_OO **(5)**, with O_2_ added to the carbon atom on either side of the delocalized propenyl radical moiety.

Six conformers exist for **(4)** (Supplementary Fig. 3). The conformer of least energy is designated **(4a)**. Only conformer **(4b)** has energy within 3 kJ mol^−1^ of **(4a)**; the other four conformers have energies 9–16 kJ mol^−1^ greater than **(4a)**. Two conformers exist for (CHI)C(CH_3_)CH_2_OO **(5)** (Supplementary Fig. 4). Conformer **(5b)** has energy 0.6 kJ mol^−1^ greater than **(5a)**. The energy of **(4a)** is greater than that of **(5a)** by 2.6 kJ mol^−1^.

**Fig. 9:**
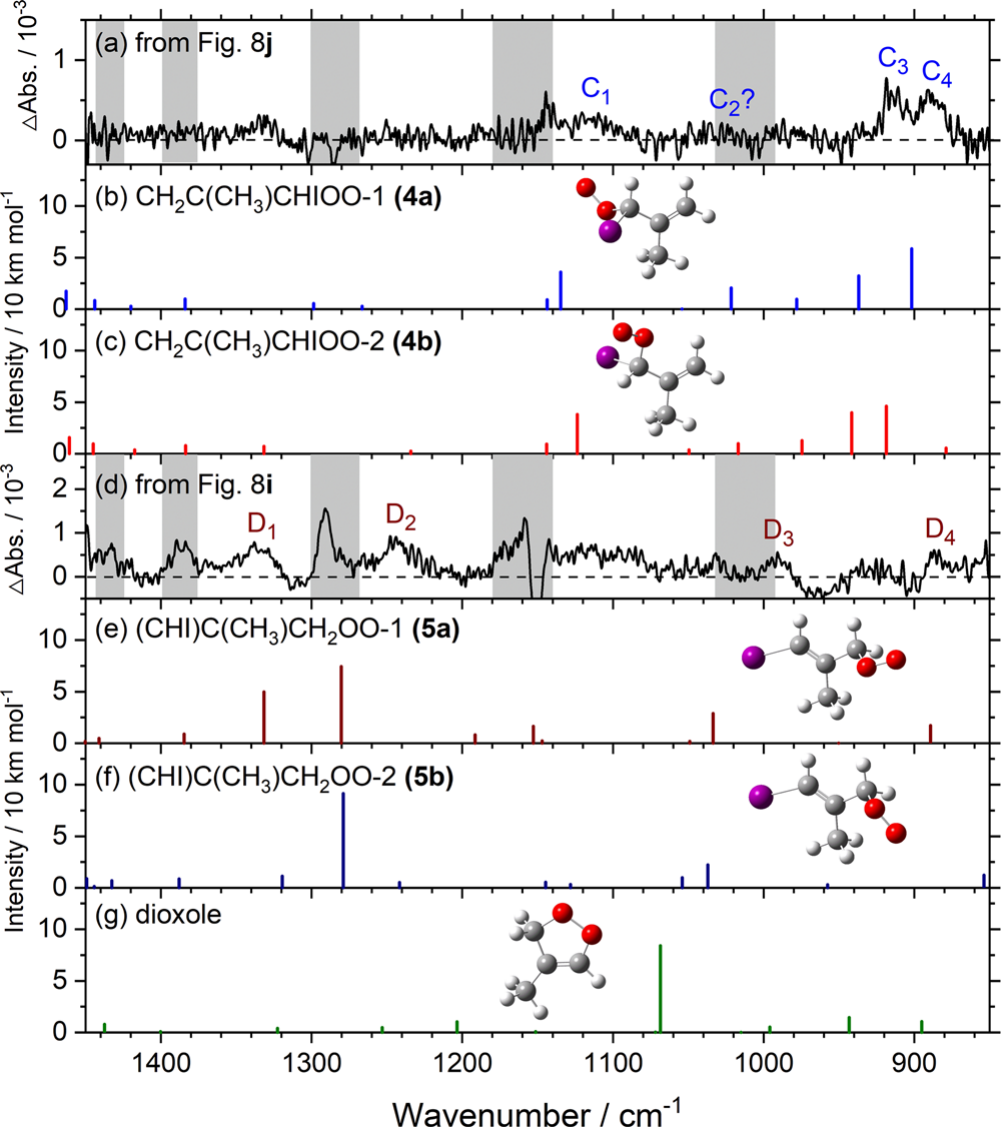
Comparison of bands in groups C and D with predicted IR stick spectra of representative isomers of iodoperoxy radicals and dioxole. **a** Experimental spectrum of group C, taken from Fig. 8j; bands are labeled C_1_–C_4_. Gray areas represent regions of possible interference from absorption of the precursor. **d** Experimental spectrum of group D, taken from Fig.8i; bands are labeled D_1_–D_4_. IR stick spectra according to scaled harmonic vibrational wavenumbers and IR intensities predicted with the B3LYP/aug-cc-pVTZ-pp method are shown for two least-energy conformers of CH_2_C(CH_3_)CHIOO, **(4a)** and **(4b)**, in **b**, **c**, both conformers of (CHI)C(CH_3_)CH_2_OO, **(5a)** and **(5b)**, in **e**, **f**, and dioxole, in **g**.

The spectra of bands in groups C and D are reproduced in Fig. 9a, d, respectively. These spectra are compared with the predicted stick spectra of the two least-energy conformers CH_2_C(CH_3_)CHIOO, **(4a)** and **(4b)**, in Fig. 9b, c, respectively, and the two conformers of (CHI)C(CH_3_)CH_2_OO, **(5a)** and **(5b)**, and dioxole in Fig. 9e–g, respectively. Observed bands C_1_‒C_4_ near 1116, 1031, 914, and 888 cm^−1^ agree satisfactorily with the scaled harmonic vibrational wavenumbers predicted for the four most intense bands of **(4a)**, near 1135, 1022, 937, and 902 cm^−1^ in this region (Table 4). We could not positively exclude a possible contribution of **(4b)** to the observed spectrum because of the similarity in predicted spectra. The other four conformers **(4c)−(4** **f)** are expected to have insignificant contributions because of greater energy.

**Table 4:** Comparison of observed vibrational wavenumbers and IR intensities of CH_2_C(CH_3_)CHIOO (**4a**) and (CHI)C(CH_3_)CH_2_OO (**5a**) in region 800‒1500 cm^−1^ with those calculated with the B3LYP/aug-cc-pVTZ-pp method.

Mode	CH_2_C(CH_3_)CHIOO (4a)				(CHI)C(CH_3_)CH_2_OO (5a)			
	Experiment		Calculation		Experiment		Calculation	
	*ν*/cm^−1^	Intensity^a^	*ν*/cm^−1b^	Intensity^c^	*ν*/cm^−1^	Intensity^a^	*ν*/cm^−1b^	Intensity^c^
*v*_8_			1463	17.5			1453	10.3
*v*_9_			1444	8.7			1450	1.9
*v*_10_			1420	2.9			1441	4.9
*v*_11_			1384	10.1			1385	9.0
*v*_12_			1299	5.6	1333	63	1332	49.9
*v*_13_			1267	3.1	1243	100	1280	74.4
*v*_14_			1144	9.3			1192	8.4
*v*_15_	1116	100	1135	36.0			1153	16.6
*v*_16_			1054	0.3			1147	2.6
*v*_17_	1031	50	1022	20.8			1049	2.0
*v*_18_			978	9.7	990	63	1034	29.0
*v*_19_	914	65	937	32.4			950	0.3
*v*_20_	888^d^	75	902	58.7	886^d^	79	889	17.3
*v*_21_			837	9.7			842	3.3

^a^Percentage of IR intensity relative to the most intense bands near 1116 and 1243 cm^−1^, respectively.

^b^Harmonic vibrational wavenumber *x* scaled according to 0.9683 *x* + 11.5; see text.

^c^In unit km mol^−1^.

^d^Bands of CH_2_C(CH_3_)CHIOO **(4a)** and (CHI)C(CH_3_)CH_2_OO **(5a)** overlap.

Observed bands D_1_‒D_4_ near 1333, 1243, 990, and 886 cm^−1^ agree satisfactorily with the four most intense bands of (CHI)C(CH_3_)CH_2_OO **(5a)** predicted near 1332, 1280, 1034, and 889 cm^−1^ in this region (Table 4). Similarly, we could not exclude positively a possible contribution of **(5b)** to the observed spectrum of group D because of the similarity in predicted spectra. Neither spectrum of group C or D shows a satisfactory agreement with that predicted for dioxole, even though we cannot exclude the possibility that dioxole might contribute to part of band C_1_.

Adducts **(4)** and **(5)** correspond to the addition of O_2_ to either carbon of the delocalized propenyl radical moiety of **(2)**; this is the first case that both adducts were observed. Because the C–I bond in **(4)** is allylic, whereas that in **(5)** is vinylic, we expect that the dissociation of the C–I bond of **(5)** has a greater barrier; **(5)** is hence expected to be more stable, as was observed experimentally.

### Relative yields of (3), (4), and (5) as a function of pressure

We performed similar experiments with O_2_ at pressures near 21, 86, 229, and 346 Torr and recorded the spectra with an external digitizer. The processed spectra for 0–5 and 30–35 μs after photolysis are compared in Supplementary Fig. 13. We estimated the relative yields of **(3)**, **(4)**, and **(5)** at varied pressures using two methods, as discussed in Supplementary Note 6 and summarized in Supplementary Table 11. The relative variations in intensities derived from spectral stripping factors for bands in groups C and D (method I) are more reliable. For **(3)**, the ratio relative to the experiment at 21.0 Torr remains similar (1.29–1.38) at 86–346 Torr. For **(4)** and **(5)**, the ratios increase with pressure, with ratios 1.91 and 1.62 at 346 Torr, respectively; **(4)** increases more than **(5)**. Unlike what was observed for CH_2_OO^24^ and MVKO^23^, our experiments show no significant increase of **(4)** at the expense of **(3)** as pressure increases. Furthermore, even though the estimate might have large errors due to uncertainties in predicted IR intensities, the yield of MACRO **(3a)** from photolysis of the precursor is significantly smaller than other carbonyl oxides, in the range 6–8% as listed in Supplementary Table 11.

**Fig. 10:**
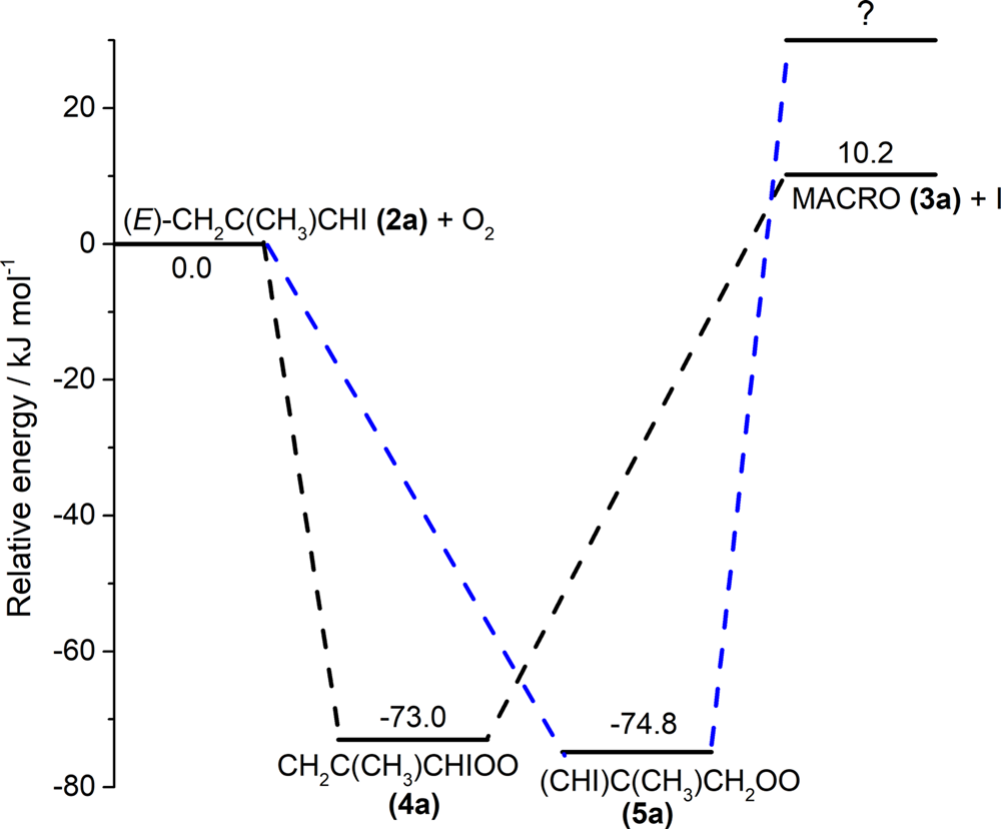
Potential energy schematics showing possible reactions of (*E*)-CH_2_C(CH_3_)CHI **(2a)** with O_2_. Relative energies (kJ mol^–1^) are computed with the CCSD(T)/aug-cc-pVTZ-pp//B3LYP/aug-cc-pVTZ-pp method and corrected with zero-point vibrational energy (ZPVE) from the harmonic vibrational wavenumbers calculated with the B3LYP/aug-cc-pVTZ-pp method.

We calculated the potential-energy scheme for the source reaction with the CCSD(T)//B3LYP/aug-cc-pVTZ-pp method, as shown in Fig. 10. The formation of adducts **(4a)** and **(5a)** from **(2a)** + O_2_ is exothermic by ~73 and 75 kJ mol^−1^, respectively, whereas the formation of **(3a)** + I is endothermic by ~10 kJ mol^−1^. This endothermicity might explain that MACRO was produced with a yield significantly smaller than other carbonyl oxides because other reactions are slightly exothermic. The smaller pressure effect for **(5)** than for **(4)** is consistent with a larger barrier for the C–I bond fission of **(5)**. It is unclear, however, why the yield of MACRO did not decrease for pressure above 21 Torr. One possibility is that O_2_ serves as a reactor instead of a quencher and some decomposition occurs at 21 Torr. Further investigations are needed to clarify this.

## Conclusion

In the absence of O_2_, upon irradiation of gaseous precursor 1,3-diiodo-2-methyl-prop-1-ene **(1)**, CH_2_IC(CH_3_)CHI, at 248 nm, iodoalkenyl radical CH_2_C(CH_3_)CHI **(2)** was produced, as characterized with bands near 1364, 1312, 1178, 1002, and 778 cm^−1^. This result provided direct spectral evidence to confirm that only the allylic C–I bond, not the vinylic C–I bond, dissociated upon photolysis through observation of only radical product **(2)**, not **(6)**, and only end product 3,4-diiodo-2,5-dimethyl-hexa-1,5-diene **(7)**, produced from the dimerization reaction of **(2)**.

When O_2_ at 21.0 Torr was added to the system, we report the identification of *anti*-*trans*-CH_2_C(CH_3_)CHOO **(3a)** with its intense infrared OO-stretching band near 917 cm^−1^, whereas some weaker bands might be tentatively attributed to the *syn-cis*-conformer **(3b)**. The observation of a significantly larger OO-stretching wavenumber provides also direct spectral support for a resonance stabilization of MACRO; a OO-stretching wavenumber smaller than that of its isomer MVKO is associated with the hyper-conjugation of MACRO that weakens the O–O bond slightly.

With O_2_ at greater pressure, iodoperoxy radical adducts 3-hydroperoxy-3-iodo-2-methyl-prop-1-ene **(4)**, CH_2_C(CH_3_)CHIOO, characterized with infrared absorption bands at 1116, 1031, 914, and 888 cm^−1^, and 3-hydroperoxy-1-iodo-2-methyl-prop-1-ene **(5)**, (CHI)C(CH_3_)CH_2_OO, characterized with infrared absorption bands at 1333, 1243, 990, and 886 cm^−1^, were observed. This spectral evidence shows O_2_ can add to either end of the propenyl radical moiety in **(2)**. As pressure increases, the yield of **(3)** remained small, whereas those of **(4)** and **(5)** increased; the enhancement with pressure was more for **(4)**, consistent with the expectation that **(5)** has a much larger barrier for the C–I fission. The much smaller yield of **(3)** than for other carbonyl oxides produced from similar reaction schemes is explained by a small endothermicity for the formation of **(3)** + I from **(2)** + O_2_; formation reactions of other carbonyl oxides are slightly exothermic.

The IR spectra of these four intermediates **(2)**−**(5)** are new; they provide valuable information to probe the associated species to understand the mechanism for the formation of carbonyl oxide MACRO from the source reaction. These spectral detections are also valuable to probe *anti*-*trans*-MACRO and associated adducts to investigate reactions of MACRO with atmospheric species in laboratories, even though the limited detectivity of this technique and the small yield of MACRO might require a much greater proportion of the precursor.

## Methods

### Experimental

The step-scan Fourier-transform infrared (FTIR) absorption technique is described in detail elsewhere^18,19^. A White cell of effective path length 3.6 m (base length 15 cm) and volume ~1370 cm^3^ was installed on the external port of the spectrometer to serve as a reactor and an absorption cell. A KrF excimer laser (248 nm, 6–11 Hz, ≈ 230 mJ pulse^−1^, beam size 1.2 × 9.3 cm^2^) was employed to photodissociate either pure (*E*)-CH_2_IC(CH_3_)CHI **(1a)** or a mixture of (*E*)-CH_2_IC(CH_3_)CHI **(1a)** and (*Z*)-CH_2_IC(CH_3_)CHI **(1b)**, denoted **(1)**. The photolysis laser beam was multiply reflected between a pair of external laser mirrors and propagated sideways, nearly perpendicular to the IR beams in the White cell.

The IR probe light from the FTIR spectrometer was detected with a HgCdTe detector at 77 K; the signal was sent to an external 14-bit digitizer with a temporal resolution 4 ns. Period 40 μs (10,000 data points) was typically covered. In some cases, an internal 24-bit digitizer (temporal resolution 12.5 µs) was used to cover a longer period with an improved SNR. We employed appropriate optical filters to limit the spectral region so as to perform undersampling to decrease the data-acquisition time. For spectral range 753–1504 cm^−1^ at instrumental resolution 1 cm^−1^, 1523 scan steps (each averaged with 6–11 laser shots) were completed in ≈50 min. The spectral width (full width at half maximum) after apodization with the Blackman-Harris 3-term function is 1.28 times the listed instrumental resolution. In all, 3‒7 spectra accumulated under similar conditions were averaged to yield a spectrum with a satisfactory SNR.

The liquid sample of **(1)** was placed in a dark flask at 298 K; a stream of gaseous N_2_ or O_2_ was passed over the sample to carry the vapor into the reactor. The partial pressures of **(1)** were estimated with Beer’s law using the observed integrated absorbance of IR bands in regions 1362–1412, 1272–1301, 759–791 cm^–1^, and calculated IR intensities. The average photolysis fraction of **(1)** was estimated to be typically ~19% according to its decrease in infrared absorbance. The decrease of the precursor upon irradiation was estimated to be (8.9‒11.1) × 10^13^ molecule cm^−3^. The flow rates were *F*_N2_ ≈ 21.4 STP cm^3^ s^–1^ (STP denotes standard temperature 273 K and pressure 1 atm) or *F*_O2_ ≈ 21.1‒73.9 STP cm^3^ s^−1^. Partial pressures were *P*_CH2IC(CH3)CHI_ ≈ 32–55 mTorr, *P*_N2_ ≈ 20.0 Torr, or *P*_O2_ = 21.0–346 Torr. (*E*)-CH_2_IC(CH_3_)CHI **(1a)** (>95%, Accela ChemBio), 1:1 mixture of (*E*)-/(*Z*)-CH_2_IC(CH_3_)CHI **(1)** (>97%, Accela ChemBio, the ratio of conformation was determined with NMR), N_2_ (99.9995%, Chiah-Lung), and O_2_ (99.99%, Chiah-Lung) were used as received. In this paper, we denote the mixture of conformers (*E*)-/(*Z*)-CH_2_IC(CH_3_)CHI as CH_2_IC(CH_3_)CHI or **(1)**; the mixture is significantly more economical than **(1a)**.

### Computational

Quantum-chemical calculations were performed with the Gaussian 16 program suite^34^. The equilibrium geometry, rotational parameters, harmonic vibrational wavenumbers, and IR intensities of all conformers of precursor **(1)**, isomers of CH_2_C(CH_3_)CHI **(2)** and CH_2_IC(CH_3_)CH **(6)**, MACRO **(3)**, dioxole, and the iodoperoxy adducts CH_2_C(CH_3_)CHIOO **(4)** and (CHI)C(CH_3_)CH_2_OO **(5)** were computed with the B3LYP density-functional theory (DFT), which uses Becke’s three-parameter hybrid exchange functional with a correlation functional of Lee et al.^35–37^. The anharmonic vibrations were calculated for isomers of MACRO with a second-order perturbation approach using an effective finite-difference evaluation of the third and semi-diagonal fourth derivatives; as MACRO contains no I atom, anharmonic vibrational calculations for these species are more practical. In both methods, the standard Dunning’s correlation-consistent basis set augmented with diffuse functions, aug-cc-pVTZ, was used^38,39^. For the iodine atom, the additional pseudopotential, indicated as pp, was implemented^40^. In some cases, the geometries obtained from the B3LYP/aug-cc-pVTZ method were used for calculations of single-point electronic energies with the coupled-cluster single-double and perturbative triple, CCSD(T), method^41^. All energies were corrected for zero-point vibrational energy (ZPVE), which was taken from the harmonic vibrational energies calculated at the B3LYP level.

## Supplementary information

Supporting Material

Peer Review File

## Data Availability

The data supporting the findings of this study are available within the paper and its Supplementary Information. All other relevant data are available from the authors upon reasonable request.
