# Supplementary material for: Formation reaction mechanism and infrared spectra of anti-trans-methacrolein oxide and its associated precursor and adduct radicals
Source: Commun Chem. 2022 Mar 4;5:26. doi: 10.1038/s42004-022-00644-0 (PMC9814089; doi:10.1038/s42004-022-00644-0)
Supplement: Supplementary file 1 — Supporting Material [file 42004_2022_644_MOESM1_ESM.pdf]

Supplementary Information

Formation reaction mechanism and infrared spectra of *anti*-*trans*-methacrolein oxide and its associated precursor and adduct radicals

Jia-Rong Cai[1], Jung-Hsuan Su[1], and Yuan-Pern Lee[1,2,3*]

[1]Department of Applied Chemistry and Institute of Molecular Science, National Yang Ming Chiao Tung University, Hsinchu 300093, Taiwan,

[2]Center for Emergent Functional Matter Science, National Yang Ming Chiao Tung University, Hsinchu 300093, Taiwan.

[3]Institute of Atomic and Molecular Sciences, Academia Sinica, Taipei 106319, Taiwan.

(Y.-P. L) : yplee@nycu.edu.tw

Table of Contents

Supplementary Note 1. Computational results --1

Supplementary Note 2. Photolysis of precursor $CH_2IC(CH_3)CHI$ (1) -------------------------1

Supplementary Note 3. Photolysis of $CH_2IC(CH_3)CHI$ (1) in O_2 at 21.0 Torr ----------------- 3

Supplementary Note 4. Spectral simulation of conformers of MACRO ------------------------4

Supplementary Note 5. Photolysis of $CH_2IC(CH_3)CHI$ (1) in O_2 at increased pressure------ 4

Supplementary Note 6. Estimates of relative yields of MACRO (3), $CH_2C(CH_3)CHIOO$ (4), and $(CHI)C(CH_3)CH_2OO$ (5)--5

Supplementary Table 1. Cartesian coordinates of optimized geometries of precursors (*E*)- and (*Z*)-$CH_2IC(CH_3)CHI$ **(1)** and iodoalkenyl radicals (*E*)- and (*Z*)-$CH_2C(CH_3)CHI$ **(2)** and (*E*)- and (*Z*)- $CH_2IC(CH_3)CH$ **(6)** predicted with the B3LYP/aug-cc-pVTZ-pp method -- 7

Supplementary Table 2. Cartesian coordinates of optimized geometries of four conformers of carbonyl oxides *anti-trans-*, *syn-cis-*, *syn-trans-*, and *anti-cis*-$CH_2C(CH_3)CHOO$ (MACRO) **(3)** and dioxole predicted with the B3LYP/aug-cc-pVTZ method ------------------- 8

Supplementary Table 3. Cartesian coordinates of optimized geometries of six conformers of iodoperoxy radical 3-hydroperoxy-3-iodo-2-methyl-prop-1-ene $CH_2C(CH_3)CHIOO$ **(4)** predicted with the B3LYP/ aug-cc-pVTZ-pp method--- 9

Supplementary Table 4. Cartesian coordinates of optimized geometries of two conformers of iodoperoxy radical 3-hydroperoxy-1-iodo-2-methyl-prop-1-ene $(CHI)C(CH_3)CH_2OO$ **(5)** predicted with the B3LYP/aug-cc-pVTZ-pp method -- 10

Supplementary Table 5. Vibrational wavenumbers and IR intensities of (*E*)- and (*Z*)-$CH_2IC(CH_3)CHI$, **(1a)** and **(1b)**, predicted with the B3LYP/aug-cc-pVTZ-pp method --------- 11

Supplementary Table 6. Vibrational wavenumbers and IR intensities of (*E*)- and (*Z*)-$CH_2C(CH_3)CHI$ **(2)** and (*E*)- and (*Z*)-$CH_2IC(CH_3)CH$ **(6)** predicted with the B3LYP/aug-cc-pVTZ-pp method -- 12

Supplementary Table 7. Vibrational wavenumbers and IR intensities of four conformers of Carbonyl oxides MACRO **(3)** and dioxole predicted with the B3LYP/aug-cc-pVTZ method -- 13

Supplementary Table 8. Vibrational wavenumbers and IR intensities of six conformers of iodoperoxy radical 3-hydroperoxy-3-iodo-2-methyl-prop-1-ene $CH_2C(CH_3)CHIOO$ **(4)** predicted with the B3LYP/ aug-cc-pVTZ-pp method--- 16

Supplementary Table 9. Vibrational wavenumbers and IR intensities of two conformers of iodoperoxy radical 3-hydroperoxy-1-iodo-2-methyl-prop-1-ene $(CHI)C(CH_3)CH_2OO$, **(5a)** and **(5b),** predicted with the B3LYP/aug-cc-pVTZ-pp method ------------------------------------- 18

Supplementary Table 10. Rotational parameters and ratios of types for each vibrational state of four conformers of Carbonyl oxides MACRO **(3)** and dioxole predicted with the B3LYP/ aug-cc-pVTZ method-- 19

Supplementary Table 11. Summary of estimates of relative abundance of species **(3)**−**(5)** observed under varied pressures--- 23
Supplementary Figure 1. Geometries of conformers of precursor $CH_2IC(CH_3)CHI$ **(1)**, iodoalkenyl radicals $CH_2C(CH_3)CHI$ **(2)** and $CH_2IC(CH_3)CH$ **(6)** predicted with the B3LYP/aug-cc-pVTZ-pp method --- 24
Supplementary Figure 2. Geometries of conformers of methacrolein oxide (MACRO) and dioxole predicted with the B3LYP/aug-cc-pVTZ method--- 25
Supplementary Figure 3. Geometries of six conformers of iodoperoxy radicals 3-hydroperoxy-3-iodo-2-methyl-prop-1-ene $CH_2C(CH_3)CHIOO$ **(4)** predicted with the B3LYP/aug-cc-pVTZ-pp method --- 26
Supplementary Figure 4. Geometries of two conformers of 3-hydroperoxy-1-iodo-2-methyl-prop-1-ene $(CHI)C(CH_3)CH_2OO$ **(5)** predicted with the B3LYP/aug-cc-pVTZ-pp method---- 27
Supplementary Figure 5. Comparison of IR spectra of gaseous (*E*)-/(*Z*)-$CH_2IC(CH_3)CHI$, **(1a)** and **(1b)**, with predicted IR stick spectra --- 28
Supplementary Figure 6. Observed and processed spectra in region 1420−780 cm^{-1} upon photolysis at 248 nm of a flowing mixture of $CH_2IC(CH_3)CHI$ **(1)**/O_2 (0.030/20.0 Torr). ----- 29
Supplementary Figure 7. Temporal evolution of observed and processed spectra in region 1450–850 cm^{-1} on photolysis at 248 nm of a flowing mixture of $CH_2IC(CH_3)CHI$ **(1)**/O_2 (0.030/21.0 Torr) -- 30
Supplementary Figure 8. Observed and processed spectra in region 1450−850 cm^{-1} upon photolysis at 248 nm of a flowing mixture of $CH_2IC(CH_3)CHI$ **(1)**/O_2 (0.030/20.0 Torr)------ 31
Supplementary Figure 9. Displacement vectors (blue arrows) and directions of dipole derivatives (yellow arrows) for modes v_{14}, v_{15}, v_{24}, and v_{25} of *anti-trans*-MACRO **(3a)** predicted with the B3LYP/aug-cc-pVTZ method--- 32
Supplementary Figure 10. Rotational contours simulated for modes v_{14}, v_{15}, v_{24}, and v_{25} of *anti-trans-* MACRO **(3a)** --- 33
Supplementary Figure 11. Resonance structures and frontier orbitals of *anti-trans*-MACRO **(3a)** and *syn-trans*-MVKO --- 34
Supplementary Figure 12. Observed and processed spectra in region 1450−850 cm^{-1} upon photolysis at 248 nm of a flowing mixture of $CH_2IC(CH_3)CHI$ **(1)**/O_2 (0.060/334 Torr)------- 35
Supplementary Figure 13. Comparison of processed spectra at various pressures recorded 0–5 and 30−35 μs in region 1450–850 cm^{-1} on photolysis at 248 nm of a flowing mixture of $CH_2IC(CH_3)CHI$ **(1)**/O_2 at 298 K--- 36
Supplementary references --37

Supplementary Note 1. Computational results

Cartesian coordinates of optimized geometries of precursors (*E*)- and (*Z*)-$CH_2IC(CH_3)CHI$ **(1)** and iodoalkenyl radicals (*E*)- and (*Z*)-$CH_2C(CH_3)CHI$ **(2)** and (*E*)- and (*Z*)-$CH_2IC(CH_3)CH$ **(6)** predicted with the B3LYP/aug-cc-pVTZ-pp method are listed in Supplementary Table 1. Those of four conformers of carbonyl oxide (Criegee intermediates) *anti-trans-*, *syn-cis-*, *syn-trans-*, and *anti-cis-* $CH_2C(CH_3)CHOO$ (MACRO) **(3)** and dioxole are listed in Supplementary Table 2. Those of conformers of iodoperoxy radicals, six for 3-hydroperoxy-3-iodo-2-methyl-prop-1-ene $CH_2C(CH_3)CHIOO$ **(4)** and two for 3-hydroperoxy-1-iodo-2-methyl-prop-1-ene $(CHI)C(CH_3)CH_2OO$ **(5)**, are listed in Supplementary Tables 3 and 4, respectively.

The geometries and relative energies of precursors (*E*)- and (*Z*)-$CH_2IC(CH_3)CHI$, **(1a)** and **(1b)**, iodoalkenyl radicals (*E*)- and (*Z*)-$CH_2C(CH_3)CHI$, **(2a)** and **(2b)**, and (*E*)- and (*Z*)-$CH_2IC(CH_3)CH$, **(6a)** and **(6b)**, are shown in Supplementary Fig. 1. Those of four conformers of Carbonyl oxides MACRO **(3)** and dioxole are shown in Supplementary Fig. 2. Those of conformers of iodoperoxy radicals, six for $CH_2C(CH_3)CHIOO$ **(4)** and two for $(CHI)C(CH_3)CH_2OO$ **(5)**, are presented in Supplementary Figs. 3 and 4, respectively. Relative energies of conformers are also listed; most values were derived from the B3LYP/aug-cc-pVTZ unless otherwise noted. Energies of four conformers of MACRO calculated with the CCSD(T)-F12/CBS(TZ-F12,QZ-F12)//B2PLYP-D3/cc-pVTZ method by Vansco *et al.*[1] are also listed for comparison.

Computed scaled harmonic vibrational wavenumbers and IR intensities of precursors (*E*)- and (*Z*)-$CH_2IC(CH_3)CHI$, **(1a)** and **(1b)**, predicted with the B3LYP/aug-cc-pVTZ-pp method are compared with experiments in Supplementary Table 5. Computed scaled harmonic vibrational wavenumbers and IR intensities of iodoalkenyl radicals (*E*)- and (*Z*)-$CH_2C(CH_3)CHI$, **(2a)** and **(2b)**, and (*E*)- and (*Z*)-$CH_2IC(CH_3)CH$, **(6a)** and **(6b)**, are shown in Supplementary Table 6. Those of four conformers of Carbonyl oxides MACRO **(3)** and dioxole are listed in Supplementary Table 7. Those of conformers of iodoperoxy radicals, six for $CH_2C(CH_3)CHIOO$ **(4)** and two for $(CHI)C(CH_3)CH_2OO$ **(5)**, are listed in Supplementary Tables 8 and 9, respectively. Rotational parameters and ratios of types for each vibrational state of four conformers of Carbonyl oxides MACRO **(3)** and dioxole predicted with the B3LYP/aug-cc-pVTZ method are listed in Supplementary Table 10.

Supplementary Note 2. Photolysis of precursor $CH_2IC(CH_3)CHI$ (1)

Precursor 1,3-diiodo-2-methyl-prop-1-ene $CH_2IC(CH_3)CHI$ **(1)** is predicted to exist in (*Z*)- and

(E)-conformations; the energy of the (E)-conformer **(1a)** is predicted to be 1.6 kJ mol^{-1} greater than that of the (Z)-conformer **(1b)** at the B3LYP level of theory. The IR spectra of gaseous precursor **(1)** and **(1a)** in region 1560–750 cm^{-1} are presented in Supplementary Figs. 5a and 5b, respectively; in the former case, **(1)** indicates a mixture of **(1a)** and **(1b)**. To derive a spectrum of **(1b)**, we subtracted a spectrum in Supplementary 5b multiplied by 0.63 from that in Supplementary Fig. 5a so that the band near 1116 cm^{-1} disappeared; the result (expanded twice in absorbance) for **(1b)** is shown in red in Supplementary Fig. 5c and compared with that of **(1a)** in grey. The spectra of **(1a)** and **(1b)** are similar and overlapped in most bands except a band of **(1a)** near 1116 cm^{-1}. The spectra of **(1a)** and **(1b)** are compared with the stick spectra of **(1a)** and **(1b)** predicted with the B3LYP/aug-cc-pVTZ method, shown in Supplementary Figs. 5d and 5e, respectively. Two most intense bands of (**1a**) are observed at 1148/1152 and 1286 cm^{-1}, in agreement with the scaled harmonic vibrational wavenumbers predicted at 1148/1152 and 1279 cm^{-1}. Three most intense bands of (**1b**) are observed at 1156, 1286, and 776 cm^{-1}, in agreement with the scaled harmonic vibrational wavenumbers predicted at 1146, 1275, and 788 cm^{-1}. A complete list of observed bands and comparison with calculations appears in Supplementary Table 5.

Supplementary Fig. 6 was reproduced from Fig. 2 in the main text. When the diiodoalkene precursor **(1)**, of which a spectrum is shown in Supplementary Fig. 6a, was irradiated with light at 248 nm, the intensity of its bands decreased significantly, as shown in Supplementary Fig. 6b as a difference spectrum obtained on the *ac*-channel recorded 0−5 μs after irradiation; negative bands indicate the destruction of the precursor, whereas the formation of products is indicated by some extremely weak positive features. The expanded spectra of products recorded 0−5, 10−15, and 30−35 μs after irradiation are shown in Supplementary Figs. 6c−e, respectively, with the negative bands truncated. The features corresponding to the primary photolysis product appeared immediately after UV irradiation and decreased in intensity with time, but those of the end products increased continuously. To minimize the interference from the destruction of the precursor, we added the bands of the precursor (Supplementary Fig. 6a) back to the spectra in Supplementary Figs. 6c−e to compensate the loss of the precursor and presented them in Supplementary Figs. 6f−h; the regions in which the absorption of the precursor might interfere are marked with grey rectangles. In these regions some features of the parent absorption could not be compensated completely, partly because of saturation and partly because some precursors might become internally excited upon irradiation, so that their absorption spectrum differed from that before irradiation. The interference is more severe for bands with large intensities.

Supplementary Note 3. Photolysis of $CH_2IC(CH_3)CHI$ (1) in O_2 at 21.0 Torr

The top trace in Supplementary Fig. 7a shows the absorption spectrum, on a reduced scale in region 1450−850 cm^{-1}, of a flowing mixture of **(1)** (30 mTorr) and O_2 (21.0 Torr) before photolysis; Supplementary Figs. 7b–d show expanded difference spectra recorded 0–5, 5–10 and 30–35 μs, respectively, after photolysis of the mixture at 248 nm; the negative bands corresponding to the destruction of the precursor are truncated. The spectrum of the iodoalkenyl radical **(2)** shown in Supplementary Fig. 6a is reproduced in Supplementary Fig. 7e for comparison; a nearly negligible contribution of **(2)** was observed because of the rapid reaction of **(2)** with O_2. Some new features appeared and their intensities attained their maxima near 5–10 μs, whereas some additional bands appeared at a later period and became more prominent in the spectrum recorded 30–35 μs after irradiation; the latter features correspond to absorption of end product methacrolein (MACR, $CH_2C(CH_3)CHO$), of which a spectrum is presented in Supplementary Fig. 7f for comparison. We stripped absorption bands of the iodoalkenyl radical **(2)** and MACR and added back the bands of the precursor **(1),** and present the resultant spectra in Supplementary Figs. 7g–i. The regions with which intense absorption of the precursor might interfere are indicated with grey rectangles. Only one prominent band near 917 cm^{-1} showed a transient nature and attained its maximum 5–10 μs after irradiation; we mark this as B_1 in Supplementary Fig. 7h. Three weaker features near 1025, 1332, and 1386 cm^{-1} were also observed, but they appeared to suffer interference from end products or parent absorption and do not show correlated intensity with that of the band near 917 cm^{-1}; we are uncertain about their association with band B_1.

To have an improved signal-to-noise ratio (SNR), we recorded the transient spectra of a similar system with an internal digitizer (12.5 μs, 24-bit); the results are shown in Fig. 6 in the main text and reproduced in Supplementary Fig. 8. The difference spectra recorded 0–12.5, 0–25, and 100–150 μs, respectively, after photolysis of a mixture of 30 mTorr **(1)** in O_2 (20.0 Torr) at 248 nm are shown in traces a−c; the negative bands corresponding to the destruction of the precursor are truncated. The similarly processed spectra, after removing **(2)** and MACR and adding back **(1)**, are presented in Supplementary Figs. 8d−f. To further eliminate contributions from other stable products, we subtracted spectrum **8f**, which has a negligible B_1 band, from spectra **8d** and **8e** to obtain spectra shown in Supplementary Figs. 8g and 8h, respectively. These two spectra are similar except for the SNR. In addition to band B_1, weak features B_2−B_4 might also belong to this group; as we are uncertain about these bands because of their small intensities and possible interference from absorption of the parent

or the end product, we indicate them with ? marks.

Supplementary Note 4. Spectral simulation of conformers of MACRO

With program PGopher[2], we simulated the rotational contours of some vibrational modes of carbonyl oxide *syn-trans-*, *syn-cis-*, *anti-trans-*, and *anti-cis-*MACRO **(3)** and dioxole using rotational parameters of the lower state (A'', B'', and C'') and upper states (A', B', and C'), and ratios of *a*-type/*b*-type/*c*-type predicted with the B3LYP/aug-cc-pVTZ method (Supplementary Table 10). The parameters employed in the simulations are J_{max} = 150, T = 298 K, Gaussian width (fwhm) = 1.28 cm^{-1} (corresponding to instrument resolution of 1.0 cm^{-1}). The weighting factors of bands of types *a*, *b*, and *c* in each resultant vibrational absorption band were determined by the squares of the projections of the dipole derivatives for each vibrational mode onto rotational axes *a*, *b* and *c*.

Displacement vectors (blue arrows) and directions of dipole derivatives (yellow arrows) for modes v_{14}, v_{15}, v_{24}, and v_{25} of *anti-trans-*MACRO **(3a)** are shown in Supplementary Fig. 9. Rotational contours simulated for these modes of *anti-trans-*MACRO **(3a)** are shown in Supplementary Fig. 10. Resonance structures and frontier orbitals of *anti-trans-*MACRO **(3a)** and *syn-trans-*MVKO are presented in Supplementary Fig. 11. The resonance structures of MVKO and MACRO indicate delocalization of π-electrons over the CCCOO skeleton; the molecular orbitals of MACRO and MVKO also show delocalization of π-electron densities over the CCCOO skeleton. For MACRO, an additional resonance structure is shown on the right; this hyper-conjugation structure is generally employed in organic chemistry. In this hyper-conjugation structure, the O−O bond has single-bond character, whereas the adjacent C=O bond has double-bond character. This contribution explains that MACRO has a longer O−O bond length and a shorter C−O length.

Supplementary Note 5. Photolysis of $CH_2IC(CH_3)CHI$ (1) in O_2 at increased pressure.

Supplementary Fig. 12 was reproduced from Fig. 8 in the main text. Supplementary Fig. 12a shows the absorption spectrum of $CH_2IC(CH_3)CHI$ **(1)**/O_2 (0.060/334 Torr) in a flowing mixture before photolysis. Supplementary Figs. 12b–d present the difference absorption spectra of this flowing mixture 0–25, 25–50, and 100–150 μs after photolysis; the spectra were recorded with an internal 24-bit digitizer with temporal resolution 12.5 μs. Supplementary Figs. 12e and 12f depict reference spectra of MACRO **(3a)** and MACR, respectively. Supplementary Figs. 12g–i show the spectra

processed from Supplementary Figs. 12b–d, with absorption of MACR stripped and that of the precursor **(1)** added back. The processed spectrum for that recorded 100−150 μs after photolysis, Supplementary Fig. 12i, shows bands of group D near 1333, 1243, 990, and 886 cm^{-1}, marked D_1−D_4; they are associated with a more stable intermediate and are assigned to 3-hydroperoxy-1-iodo-2-methyl-prop-1-ene **(5),** $(CHI)C(CH_3)CH_2OO$, in the main text.

When we subtracted this spectrum in Supplementary Fig. 12i from that in Supplementary Fig. 12h, recorded 25−50 μs after photolysis, the resultant spectrum is shown in Supplementary Fig. 12j. This spectrum shows bands of group C near 1116, 1031, 914, and 888 cm^{-1}, marked C_1−C_4 in Supplementary Fig. 12j; band C_2 is uncertain because of its small intensity and possible interference from the parent absorption. These features in group C are associated with a less stable intermediate and are assigned to 3-hydroperoxy-3-iodo-2-methyl-prop-1-ene **(4),** $CH_2C(CH_3)CHIOO$, in the main text.

Supplementary Note 6. Estimates of relative yields of MACRO (3), $CH_2C(CH_3)CHIOO$ (4), and $(CHI)C(CH_3)CH_2OO$ (5)

Supplementary Fig. 13 shows the resultant spectra recorded 0−5 and 30−35 μs experiments with O_2 at pressures near 21, 86, 229, and 346 Torr recorded with an external digitizer; the spectra were processed with absorption of iodoalkenyl radical **(2)** and end product MACR removed and precursor **(1)** added back. Bands in groups C and D are marked in Supplementary Fig. 13d; band B_1 overlaps with band C_3 and band D_4 overlaps with band C_4. We employed processed spectra recorded 0−5 μs after photolysis to estimate the relative variations of MACRO **(3)** and iodoperoxy adducts $CH_2C(CH_3)CHIOO$ **(4)** and $(CHI)C(CH_3)CH_2OO$ **(5)** at varied pressures. Bands of the depletion of precursor **(1)** (1272−1301 cm^{-1}) were integrated to indicate decrease in absorbance of **(1)**, listed in line 4 of Supplementary Table 11; lines 1−3 list experimental conditions in four experiments.

Two methods were used for the estimates of relative yields; these methods differed by how we estimated the overlapping bands. In method I, because some bands of groups B, C, and D were overlapped, we used spectral stripping to obtain the relative populations of **(4)** and **(5)** in Supplementary Fig. 13; the reference spectra of groups C and D, corresponding to **(4)** and **(5)**, were those in Supplementary Figs. 12j and 12i, respectively. The factors for spectral stripping of bands in groups C and D are listed in lines 6 and 7 of Supplementary Table 11, respectively. This method is

more reliable than simply integrating a specific band for comparison because the entire spectral pattern including all bands was considered. Both **(4)** and **(5)** increase with pressure; **(4)** appeared to increase more than **(5)** at higher pressure. We then integrated band B_1 from the spectra with band C_3 in group C spectrally stripped; the results are shown in line 5. After taking into account the small variations in the initial loss of **(1)**, represented by the decrease of the absorbance of **(1)** in line 4, in each experiment, we derived the relative proportions of **(3)**−**(5)** in four experiments at varied pressures, as shown in lines 8−10, respectively. The errors in these estimates are estimated to be within 20 %, mainly from the errors in baseline and stripping factors.

In Method II we integrate the absorbance of representative bands after stripping the spectrum of bands in group D corresponding to species **(5)**, which is more reliable than those in group C. Bands B_1/C_3 (895–925 cm^{-1}), C_4 (880–895 cm^{-1}), and C_1 (1083–1138 cm^{-1}) were integrated, and band D_1 (1324–1344 cm^{-1}) was derived from the stripping factors listed in line 7, as listed in lines 11−14.

We employed two methods to remove the contribution of band C_3 from the integrated absorbance of band B_1/C_3. In method II-C_4, we used band C_4 (with band D_4 deducted, as stated previously) as a reference to estimate the integrated absorbance of band C_3. We relied on the calculated IR intensities of bands C_4 and C_3, 58.7 and 32.4 km mol^{-1}, respectively, to estimate the integrated absorbance of band C_3 from that of band C_4, as listed in line 15. In method II-C_1, we used band C_1 as a reference to estimate the integrated absorbance of band C_3; IR intensities of bands C_1 and C_3 are 36.0 and 32.4 km mol^{-1}, respectively. The estimated integrated absorbance of band C_3 are listed in line 16. The integrated absorbance of band B_1 with that of C_3 deducted, derived from methods II-C_4 and II-C_1 are listed in lines 17 and 18, respectively. The ratios of integrated band B_1 normalized to experiment 1 from these two methods are listed in lines 19 and 20. Methods I (line 8) and II-C_4 (line 19) gave more consistent ratios and are considered to be more reliable. Similar consistency was found for the variations of **(4)** derived from method I (line 9) and method II-C_4 (line 21). Values from method II-C_1 (lines 20 and 22) are significantly different, indicating that perhaps band C_1 is not "clean", with impurity bands increasing with pressure, as shown in line 22.

We also derived the loss of **(1)** upon photolysis to estimate the partial pressure of **(2)** thus produced, as listed in line 23; integrated absorbance of three bands were used to minimize the error in predicted IR intensities of **(1)**. Similarly, we estimated the partial pressure of **(3)** from line 5 and the predicted IR intensity of **(3a)**. The ratio of values in line 24 to those in line 23 yielded the estimated percentage yield of **(3a)**. Even though this method has significant errors, the values 5.8−8.2 % indicate relatively small yield of MACRO compared to those of smaller carbonyl oxides.

Supplementary Table 1 Cartesian coordinates of optimized geometries of precursors (*E*)- and (*Z*)-$CH_2IC(CH_3)CHI$ (1) and iodoalkenyl radicals (*E*)- and (*Z*)-$CH_2C(CH_3)CHI$ (2) and (*E*)- and (*Z*)- $CH_2IC(CH_3)CH$ (6) predicted with the B3LYP/aug-cc-pVTZ-pp method.

	x	*y*	*z*	*x*	*y*	*z*
	(*E*)-$CH_2IC(CH_3)CHI$ **(1a)**			(*Z*)-$CH_2IC(CH_3)CHI$ **(1b)**		
C1	0.02413	0.62635	0.49584	-0.01196	1.52828	0.15510
C2	0.99751	-0.26536	0.68743	1.21626	1.30746	-0.31521
C3	0.11914	1.86938	-0.33440	-0.71290	2.82166	-0.17671
C4	-1.26638	0.38425	1.19850	-0.74759	0.57914	1.02150
H1	0.86009	-1.14595	1.29612	1.73323	2.02944	-0.92791
H2	-0.63619	1.85664	-1.12236	-1.65523	2.62233	-0.69090
H3	-0.07523	2.75141	0.28089	-0.95142	3.37278	0.73615
H4	1.09710	1.97880	-0.79420	-0.10287	3.45961	-0.81274
H5	-1.22305	-0.42456	1.91694	-0.12751	-0.20540	1.43477
H6	-1.66875	1.28163	1.65704	-1.31466	1.07799	1.79967
I1	2.94252	-0.18996	-0.11511	2.38814	-0.41793	-0.01323
I2	-2.89725	-0.22486	-0.17769	-2.31350	-0.52124	-0.09332
	(*E*)-$CH_2C(CH_3)CHI$ **(2a)**			(*Z*)-$CH_2C(CH_3)CHI$ **(2b)**		
C1	1.95592	-0.08017	0.00000	-1.93051	0.08631	0.00000
C2	0.77330	-0.81318	0.00004	-0.78431	-0.70703	0.00000
C3	1.94861	1.42755	0.00001	-3.24891	-0.66891	0.00000
C4	3.15668	-0.76124	-0.00002	-1.93103	1.45857	0.00000
H1	0.77092	-1.89172	0.00003	-0.83720	-1.78425	0.00000
H2	2.96392	1.81846	-0.00003	-4.08990	0.02188	0.00000
H3	1.42946	1.81697	-0.87679	-3.33522	-1.30747	-0.88009
H4	1.42950	1.81699	0.87682	-3.33521	-1.30747	0.88009
H5	3.19046	-1.84176	-0.00003	-1.01167	2.02497	0.00000
H6	4.09844	-0.23222	-0.00001	-2.85927	2.01044	0.00000
I1	-1.14886	-0.00235	0.00000	1.18560	-0.01267	0.00000
	(*E*)-$CH_2IC(CH_3)CH$ **(6a)**			(*Z*)-$CH_2IC(CH_3)CH$ **(6b)**		
C1	2.00613	-0.14126	0.03849	-1.93252	0.16287	0.00000
C2	2.75714	-1.04612	-0.54339	-1.73346	1.45569	0.00000
C3	2.31300	1.33386	-0.00755	-3.32355	-0.44821	0.00000
C4	0.81573	-0.57707	0.83654	-0.82540	-0.85168	-0.00001
H1	2.76807	-2.11604	-0.67468	-2.33138	2.35191	0.00000
H2	1.49047	1.87958	-0.47297	-3.46631	-1.07840	-0.88106
H3	2.43643	1.73027	1.00311	-3.46630	-1.07841	0.88106
H4	3.22279	1.52658	-0.57035	-4.09237	0.32157	0.00002
H5	0.73395	-1.65252	0.92861	-0.86229	-1.48551	-0.88324
H6	0.78025	-0.10542	1.81419	-0.86231	-1.48553	0.88322
I1	-1.10913	0.02493	-0.07495	1.16926	0.01023	0.00000

Supplementary Table 2 Cartesian coordinates of optimized geometries of four conformers of Carbonyl oxides *anti-trans-*, *syn-cis-*, *syn-trans-*, and *anti-cis*-$CH_2C(CH_3)CHOO$ (MACRO) (3) and dioxole predicted with the B3LYP/aug-cc-pVTZ method.

	x	*y*	*z*	*x*	*y*	*z*
	anti-trans-MACRO **(3a)**			*syn-cis*-MACRO **(3b)**		
C1	0.88217	-0.47203	0.00000	0.00000	0.80690	0.00000
C2	0.00000	0.66153	0.00000	0.82489	-0.36438	0.00000
C3	0.28982	-1.85289	0.00000	0.76636	2.11113	0.00000
C4	2.20388	-0.22737	0.00000	-1.34870	0.76719	0.00000
H1	0.34940	1.68896	0.00000	1.90446	-0.26260	0.00000
H2	1.07420	-2.60640	0.00000	0.08218	2.95675	0.00000
H3	-0.34253	-2.00450	0.87554	1.40726	2.19623	0.87964
H4	-0.34253	-2.00450	-0.87554	1.40726	2.19623	-0.87964
H5	2.59136	0.78247	0.00000	-1.87493	-0.17083	0.00000
H6	2.92501	-1.03232	0.00000	-1.90675	1.69538	0.00000
O1	-1.25316	0.48201	0.00000	0.49886	-1.59760	0.00000
O2	-2.06061	1.58310	0.00000	-0.80821	-1.96943	0.00000
	syn-trans-MACRO (3c)			*anti-cis*-MACRO **(3d)**		
C1	0.00000	0.87304	0.00000	-0.67370	-0.69722	0.00000
C2	-0.94882	-0.21411	0.00000	0.00000	0.58397	0.00000
C3	1.48980	0.66850	0.00000	-2.17912	-0.62697	0.00000
C4	-0.55452	2.10075	0.00000	0.00573	-1.85380	0.00000
H1	-2.00905	0.01231	0.00000	-0.53843	1.52694	0.00000
H2	1.99182	1.63557	0.00000	-2.61241	-1.62455	0.00000
H3	1.80438	0.09467	0.86886	-2.54891	-0.09491	0.87885
H4	1.80438	0.09467	-0.86886	-2.54891	-0.09491	-0.87885
H5	-1.62683	2.24314	0.00000	1.08585	-1.87804	0.00000
H6	0.05765	2.99151	0.00000	-0.51483	-2.80083	0.00000
O1	-0.75219	-1.46750	0.00000	1.26211	0.66371	0.00000
O2	0.50955	-1.98761	0.00000	1.83291	1.90259	0.00000
	dioxole					
C1	-0.70732	0.06321	0.00000			
C2	0.11213	1.10321	0.00000			
C3	-2.19523	0.02560	0.00000			
C4	0.16108	-1.16401	0.00000			
H1	-0.09425	2.16262	0.00000			
H2	-2.61498	1.03050	0.00000			
H3	-2.58068	-0.49941	-0.87851			
H4	-2.58068	-0.49941	0.87851			
H5	0.01778	-1.78632	-0.89235			
H6	0.01778	-1.78632	0.89236			
O1	1.44073	0.81449	0.00000			
O2	1.51066	-0.66320	0.00000			

Supplementary Table 3 Cartesian coordinates of optimized geometries of six conformers of iodoperoxy radical 3-hydroperoxy-3-iodo-2-methyl-prop-1-ene $CH_2C(CH_3)CHIOO$ (4) predicted with the B3LYP/ aug-cc-pVTZ-pp method.

	x	*y*	*z*	*x*	*y*	*z*
	$CH_2C(CH_3)CHIOO$ **(4a)**			$CH_2C(CH_3)CHIOO$ **(4b)**		
C1	-1.81609	-0.56788	-0.13025	1.80422	-0.59731	-0.01911
C2	-0.77029	0.45400	-0.41194	0.79139	0.40693	0.40717
C3	-2.08718	-0.93128	1.30080	1.84388	-1.85424	0.80445
C4	-2.46590	-1.10187	-1.16336	2.63149	-0.36841	-1.03790
H1	-0.72294	0.78034	-1.44278	0.73456	0.56189	1.47903
H2	-1.19050	-1.33165	1.77706	1.99985	-1.62697	1.86186
H3	-2.39205	-0.05617	1.87642	0.90601	-2.40693	0.72935
H4	-2.87601	-1.67786	1.36267	2.65197	-2.50205	0.47153
H5	-2.25119	-0.81588	-2.18429	2.59706	0.54005	-1.61958
H6	-3.23033	-1.85245	-1.01517	3.37789	-1.10204	-1.30927
O1	-0.98122	1.60605	0.41999	1.00321	1.67066	-0.22060
O2	-0.54523	2.71704	-0.13731	0.64311	2.67912	0.54882
I1	1.27757	-0.31602	-0.00390	-1.28045	-0.26008	-0.09748
	$CH_2C(CH_3)CHIOO$ **(4c)**			$CH_2C(CH_3)CHIOO$ **(4d)**		
C1	-1.78724	-0.59142	-0.02931	1.78537	-0.59414	0.00034
C2	-0.70632	0.14312	-0.75572	0.73706	0.19908	0.71023
C3	-2.10659	-0.23054	1.39116	1.73040	-2.07680	0.25130
C4	-2.42926	-1.55051	-0.69703	2.72474	-0.02642	-0.75293
H1	-0.63025	-0.16461	-1.79146	0.63803	-0.09485	1.75098
H2	-1.22341	-0.31402	2.02546	1.76826	-2.29776	1.32089
H3	-2.45249	0.80080	1.46945	0.80495	-2.50885	-0.13257
H4	-2.88223	-0.88710	1.78079	2.56886	-2.57588	-0.22956
H5	-2.18873	-1.79543	-1.72294	2.77280	1.03716	-0.92713
H6	-3.21441	-2.12829	-0.22840	3.48518	-0.63612	-1.22133
O1	-0.93685	1.56911	-0.86014	0.98645	1.60861	0.78719
O2	-0.83932	2.23361	0.27034	0.85620	2.25420	-0.35399
I1	1.30146	-0.23693	0.07040	-1.29518	-0.16673	-0.09963
	$CH_2C(CH_3)CHIOO$ **(4e)**			$CH_2C(CH_3)CHIOO$ **(4f)**		
C1	1.74102	-0.81210	0.02253	-1.71959	-0.73896	-0.04509
C2	0.76003	0.29932	0.27750	-0.69675	0.22622	-0.57656
C3	3.14742	-0.45228	0.43471	-3.12512	-0.42033	-0.49537
C4	1.42480	-1.98658	-0.50769	-1.43263	-1.79668	0.70189
H1	0.83338	0.70528	1.28164	-0.67282	0.22822	-1.66342
H2	3.50387	0.42760	-0.10312	-3.46728	0.52920	-0.08257
H3	3.19504	-0.21796	1.50106	-3.18364	-0.33824	-1.58348
H4	3.82861	-1.27680	0.23480	-3.81129	-1.20086	-0.17372
H5	0.41411	-2.23463	-0.79498	-0.42767	-2.01810	1.02736
H6	2.18387	-2.74128	-0.66229	-2.21495	-2.47633	1.01130
O1	1.07773	1.38218	-0.63356	-1.02478	1.60776	-0.27523
O2	0.94414	2.56945	-0.07855	-1.29004	1.81932	0.99622
I1	-1.36931	-0.16161	0.05429	1.39888	-0.10872	-0.03420

Supplementary Table 4 Cartesian coordinates of optimized geometries of two conformers of iodoperoxy radical 3-hydroperoxy-1-iodo-2-methyl-prop-1-ene (CHI)C(CH_3)CH_2OO (5) predicted with the B3LYP/aug-cc-pVTZ-pp method.

	x	*y*	*z*	*x*	*y*	*z*
	(CHI)C(CH_3)CH_2OO **(5a)**			(CHI)C(CH_3)CH_2OO **(5b)**		
C1	1.13377	0.24770	0.31032	1.17454	0.01277	0.36077
C2	0.10460	-0.59459	0.28043	0.07502	-0.72509	0.22733
C3	1.08801	1.72724	0.08237	1.26288	1.50559	0.30448
C4	2.48443	-0.32889	0.62383	2.46436	-0.72383	0.60734
H1	0.22162	-1.65415	0.44920	0.09301	-1.80307	0.27688
H2	1.42620	2.25879	0.97577	1.59752	1.90191	1.26644
H3	1.76266	2.00714	-0.72874	2.00127	1.81800	-0.43573
H4	0.08728	2.07030	-0.16470	0.30645	1.95787	0.05715
H5	2.98365	0.20662	1.43175	3.01507	-0.32229	1.45682
H6	2.44952	-1.39213	0.85149	2.30912	-1.79422	0.72203
O1	3.33883	-0.16981	-0.56470	3.35614	-0.60682	-0.55493
O2	4.59593	-0.44318	-0.29212	4.21115	0.38717	-0.43408
I1	-1.91083	-0.09248	-0.07060	-1.88154	-0.00788	-0.08360

Supplementary Table 5 Vibrational wavenumbers and IR intensities of (*E*)- and (*Z*)-$CH_2IC(CH_3)CHI$, (1a) and (1b), predicted with the B3LYP/aug-cc-pVTZ-pp method.

mode	(*E*)-$CH_2IC(CH_3)CHI$ **(1a)**				(*Z*)-$CH_2IC(CH_3)CHI$ **(1b)**			
	experiment		calculation		experiment		calculation	
	ν/cm^{-1}	intensity[a]	ν^{b}/cm^{-1}	intensity[c]	ν/cm^{-1}	intensity[a]	ν^{b}/cm^{-1}	intensity[c]
ν_1			3109	6.7			3114	6.6
ν_2			3086	0.5			3093	0.2
ν_3			3044	5.6			3027	14.2
ν_4			3016	4.1			3023	3.1
ν_5			2986	7.6			2985	9.3
ν_6			2942	14.7			2938	22.8
ν_7			1610	33.3			1608	11.7
ν_8	1435	14	1453	9.2			1453	3.1
ν_9			1449	1.2	1451	31	1449	14.5
ν_{10}			1437	2.5	1436	38	1438	8.0
ν_{11}	1388	26	1385	12.4	1381	12	1379	3.9
ν_{12}	1286	87	1279	67.8	1286	49	1275	30.2
	1167							
ν_{13}	1152	46	1152	44.1	1167	39	1167	21.6
ν_{14}	1148	100	1148	37.0	1156	100	1146	56.2
ν_{15}	1116	13	1115	15.9	1101	6	1097	0.7
ν_{16}	1031	9	1038	7.2	1029	10	1035	5.6
ν_{17}	1004	21	997	25.8	1011	21	1007	14.7
ν_{18}	851	13	851	7.8	859	4	853	3.6
ν_{19}	819	8	813	2.7			812	0.8
ν_{20}	781	32	798	9.1	776	62	788	30.3
ν_{21}			666	11.0			665	45.1
ν_{22}			562	29.7			607	16.3
ν_{23}			436	30.1			416	2.3
ν_{24}			416	10.3			393	6.5
ν_{25}			302	2.2			305	1.3
ν_{26}			204	0.4			232	0.2
ν_{27}			192	0.1			196	0.4
ν_{28}			126	0.7			161	0.3
ν_{29}			98	0.6			146	1.9
ν_{30}			53	0.4			42	0.2

[a]Percentage IR intensities relative to the most intense band near 1150 cm^{-1}.

[b]Harmonic vibrational wavenumber *x* scaled according to (0.9683 ± 0.0116) *x* + (11.5 ± 13.8); see text.

[c] in unit km mol^{-1}.

Supplementary Table 6 Vibrational wavenumbers and IR intensities of (*E*)- and (*Z*)-$CH_2C(CH_3)CHI$ (2) and (*E*)- and (*Z*)-$CH_2IC(CH_3)CH$ (6) predicted with the B3LYP/aug-cc-pVTZ-pp method.

mode	$CH_2C(CH_3)CHI$				$CH_2IC(CH_3)CH$			
	(*E*)-conformer **(2a)**[a]		(*Z*)-conformer **(2b)**		(*E*)-conformer **(6a)**[a]		(*Z*)-conformer **(6b)**	
	ν [b]/cm^{-1}	intensity[c]	ν [b]/cm^{-1}	intensity[c]	ν [b]/cm^{-1}	intensity[c]	ν [b]/cm^{-1}	intensity[c]
ν_1	3144	7.0	3152	5.8	3144	0.4	3151	0.1
ν_2	3116	4.3	3117	3.3	3085	0.5	3032	0.5
ν_3	3056	6.6	3065	3.7	3037	11.1	3026	13.6
ν_4	3028	16.6	3024	11.4	3009	3.7	2985	10.7
ν_5	2999	8.3	2999	12.8	2989	10.1	2980	14.3
ν_6	2951	10.1	2949	25.4	2941	16.0	2932	24.0
ν_7	1482	5.0	1489	7.4	1627	2.5	1651	10.6
ν_8	1458	21.2	1461	11.4	1451	12.0	1445	8.8
ν_9	1448	11.1	1451	7.3	1447	6.0	1445	10.4
ν_{10}	1388	5.2	1377	1.1	1432	2.9	1420	3.3
ν_{11}	1355	2.0	1345	12.7	1375	6.7	1366	0.5
ν_{12}	1304	37.1	1307	16.5	1184	14.5	1199	24.5
ν_{13}	1187	36.5	1169	32.5	1145	39.1	1133	2.9
ν_{14}	1039	0.0	1039	0.3	1115	4.1	1123	14.1
ν_{15}	1004	7.7	1013	6.9	1026	1.7	997	2.0
ν_{16}	993	0.8	992	5.0	998	2.2	976	2.7
ν_{17}	853	3.4	860	0.9	858	53.2	839	9.6
ν_{18}	793	46.1	817	32.4	802	11.6	803	21.4
ν_{19}	665	26.7	671	16.1	782	5.0	797	0.7
ν_{20}	627	9.0	604	7.6	681	23.9	679	34.1
ν_{21}	548	1.2	565	5.8	558	40.8	613	11.3
ν_{22}	477	11.9	474	15.0	428	3.6	409	3.3
ν_{23}	451	0.1	462	3.5	382	3.5	406	9.1
ν_{24}	324	0.7	296	1.6	344	2.5	278	0.2
ν_{25}	179	0.1	192	0.2	178	0.2	185	0.0
ν_{26}	152	0.7	148	0.5	157	1.3	147	0.6
ν_{27}	142	0.0	45	0.1	94	0.7	78	0.0

[a]This conformer has a planar symmetry, but we ordered the vibrational modes without considering that symmetry to match those of the other conformer.

[b]Harmonic vibrational wavenumber x scaled according to $y = (0.9683 \pm 0.0116)\, x + (11.5 \pm 13.8)$; see text.

[c]in unit km mol^{-1}.

Supplementary Table 7 Vibrational wavenumbers and IR intensities of four conformers of Carbonyl oxides MACRO (3) and dioxole predicted with the B3LYP/aug-cc-pVTZ method.

mode	sym.	*anti-trans*-MACRO **(3a)**			*syn-cis*-MACRO **(3b)**		
		harmonic[a]	anharmonic	intensity[b]	harmonic[a]	anharmonic	intensity[b]
ν_1	a'	3143	3098	2.8	3188	3129	3.5
ν_2	a'	3068	3027	1.9	3074	3041	4.4
ν_3	a'	3058	2986	0.9	3055	3010	15.2
ν_4	a'	3036	2991	9.5	3030	2981	7.4
ν_5	a'	2956	2939	8.0	2944	2938	16.1
ν_6	a'	1614	1616	6.9	1589	1586	13.2
ν_7	a'	1484	1481	2.3	1492	1481	43.5
ν_8	a'	1453	1449	15.4	1457	1452	6.7
ν_9	a'	1408	1406	4.8	1396	1406	15.0
ν_{10}	a'	1391	1392	4.5	1380	1383	12.4
ν_{11}	a'	1356	1352	4.4	1350	1346	12.4
ν_{12}	a'	1273	1276	5.8	1247	1252	2.3
ν_{13}	a'	1033	1034	0.3	1022	1030	15.5
ν_{14}	a'	983	985	16.7	986	978	38.1
ν_{15}	a'	946	944	200.6	910	907	81.0
ν_{16}	a'	848	848	7.4	884	884	14.0
ν_{17}	a'	564	565	12.5	693	699	1.8
ν_{18}	a'	473	476	0.3	419	416	0.6
ν_{19}	a'	368	372	1.4	342	337	6.1
ν_{20}	a'	193	187	10.8	285	277	7.0
ν_{21}	a"	3005	2956	5.7	2990	2936	12.3
ν_{22}	a"	1445	1438	8.6	1452	1443	7.3
ν_{23}	a"	1052	1048	0.4	1056	1053	0.4
ν_{24}	a"	950	950	26.2	988	982	26.7
ν_{25}	a"	930	924	14.7	893	876	5.2
ν_{26}	a"	684	683	0.4	690	683	2.3
ν_{27}	a"	504	501	13.4	539	525	14.9
ν_{28}	a"	230	224	0.4	326	311	1.1
ν_{29}	a"	193	182	1.9	140	240	1.0
ν_{30}	a"	159	138	2.7	114	6	5.4

mode	sym.	*syn-trans*-MACRO **(3c)**			*anti-cis*-MACRO **(3d)**		
		harmonic[a]	anharmonic	intensity[b]	harmonic[a]	anharmonic	intensity[b]
ν_1	a'	3140	3090	4.6	3151	3097	0.8
ν_2	a'	3076	3056	3.1	3066	3000	0.6
ν_3	a'	3057	3007	1.5	3060	3022	6.3
ν_4	a'	3033	2991	6.1	3033	3005	8.8
ν_5	a'	2963	2929	21.6	2944	2946	12.2
ν_6	a'	1601	1596	8.8	1623	1624	9.9
ν_7	a'	1495	1490	34.1	1478	1476	8.2
ν_8	a'	1448	1443	12.1	1460	1454	27.2
ν_9	a'	1407	1410	0.5	1412	1405	6.0
ν_{10}	a'	1390	1386	10.0	1386	1395	4.9
ν_{11}	a'	1352	1356	12.6	1336	1345	17.4
ν_{12}	a'	1310	1308	5.9	1243	1247	0.1
ν_{13}	a'	1039	1041	22.4	1025	1030	3.6
ν_{14}	a'	979	985	15.0	992	993	26.8
ν_{15}	a'	884	881	147.1	953	951	103.0
ν_{16}	a'	824	820	21.1	880	875	48.6
ν_{17}	a'	711	711	2.2	550	550	25.7
ν_{18}	a'	428	433	2.0	454	459	12.2
ν_{19}	a'	350	340	0.3	369	373	1.1
ν_{20}	a'	256	242	13.7	204	195	6.7
ν_{21}	a"	3034	2974	2.5	2989	2950	10.1
ν_{22}	a"	1433	1422	8.2	1448	1438	7.7
ν_{23}	a"	1054	1049	0.5	1055	1049	0.4
ν_{24}	a"	943	936	39.5	949	921	10.0
ν_{25}	a"	878	874	1.5	948	947	26.1
ν_{26}	a"	688	631	2.0	666	591	1.3
ν_{27}	a"	550	539	12.7	494	500	14.2
ν_{28}	a"	333	321	1.0	203	221	1.6
ν_{29}	a"	209	166	0.6	153	109	0.7
ν_{30}	a"	75	18	5.4	96	10	2.8

mode	sym.	dioxole		

		harmonic[a]	anharmonic	intensity[b]
ν_1	a	3136	3094	0.7
ν_2	a	3014	2973	15.6
ν_3	a	2959	2909	19.9
ν_4	a	2925	2900	44.4
ν_5	a	2901	2835	40.3
ν_6	a	2880	2800	67.1
ν_7	a	1694	1692	28.0
ν_8	a	1488	1478	2.7
ν_9	a	1460	1455	7.9
ν_{10}	a	1443	1438	8.1
ν_{11}	a	1393	1400	0.9
ν_{12}	a	1330	1323	4.2
ν_{13}	a	1261	1253	4.8
ν_{14}	a	1212	1204	10.5
ν_{15}	a	1148	1151	0.9
ν_{16}	a	1080	1069	84.1
ν_{17}	a	1070	1072	0.5
ν_{18}	a	1009	1015	0.3
ν_{19}	a	990	996	5.4
ν_{20}	a	947	943	14.7
ν_{21}	a	903	895	10.8
ν_{22}	a	819	814	16.9
ν_{23}	a	793	785	8.4
ν_{24}	a	768	769	8.3
ν_{25}	a	593	595	3.6
ν_{26}	a	462	457	11.4
ν_{27}	a	306	314	0.9
ν_{28}	a	260	274	0.7
ν_{29}	a	194	179	0.0
ν_{30}	a	57	280	2.7

[a]Harmonic vibrational wavenumber x scaled according to $y = (0.9683 \pm 0.0116)\,x + (11.5 \pm 13.8)$; see text.

[b]In unit km mol^{-1}.

Supplementary Table 8 Vibrational wavenumbers and IR intensities of six conformers of iodoperoxy radical 3-hydroperoxy-3-iodo-2-methyl-prop-1-ene $CH_2C(CH_3)CHIOO$ (4) predicted with the B3LYP/ aug-cc-pVTZ-pp method.

mode	$CH_2C(CH_3)CHIOO$ **(4a)**		$CH_2C(CH_3)CHIOO$ **(4b)**		$CH_2C(CH_3)CHIOO$ **(4c)**	
	ν [a] /cm^{-1}	intensity[b]	ν [a] /cm^{-1}	intensity[b]	ν [a] /cm^{-1}	intensity[b]
ν_1	3132	5.7	3150	2.2	3130	6.4
ν_2	3073	1.3	3066	4.1	3065	0.3
ν_3	3053	2.8	3048	1.3	3051	3.7
ν_4	3031	13.0	3030	12.9	3027	14.9
ν_5	2998	6.1	2988	8.4	3002	4.7
ν_6	2950	8.7	2941	14.2	2953	7.4
ν_7	1653	7.9	1652	5.3	1645	7.1
ν_8	1463	17.5	1461	15.9	1459	16.2
ν_9	1444	8.7	1445	9.9	1441	9.7
ν_{10}	1420	2.9	1417	3.9	1419	1.2
ν_{11}	1384	10.1	1384	8.0	1386	10.6
ν_{12}	1299	5.6	1332	7.4	1319	4.6
ν_{13}	1267	3.1	1234	2.7	1272	1.2
ν_{14}	1144	9.3	1144	9.6	1156	31.4
ν_{15}	1135	36.0	1124	38.3	1096	7.1
ν_{16}	1054	0.3	1050	4.0	1053	0.1
ν_{17}	1022	20.8	1017	10.0	1017	12.6
ν_{18}	978	9.7	975	12.8	976	9.3
ν_{19}	937	32.4	942	39.8	937	39.1
ν_{20}	902	58.7	919	46.3	865	25.5
ν_{21}	837	9.7	879	5.6	788	51.4
ν_{22}	731	24.7	732	25.8	736	35.6
ν_{23}	588	61.3	588	50.5	705	4.3
ν_{24}	537	2.0	520	9.9	572	31.8
ν_{25}	489	9.1	471	2.6	453	7.7
ν_{26}	404	10.2	420	12.2	388	4.6
ν_{27}	339	4.7	356	2.4	350	4.1
ν_{28}	246	0.7	234	2.8	245	1.9
ν_{29}	202	1.8	228	0.3	221	1.0
ν_{30}	177	0.3	171	0.3	185	1.0
ν_{31}	148	1.5	151	1.4	148	1.3
ν_{32}	81	0.3	72	1.0	108	0.2
ν_{33}	72	0.9	66	0.1	75	0.2

mode	$CH_2C(CH_3)CHIOO$ **(4d)**		$CH_2C(CH_3)CHIOO$ **(4e)**		$CH_2C(CH_3)CHIOO$ **(4f)**	
	ν [a] /cm^{-1}	intensity[b]	ν [a] /cm^{-1}	intensity[b]	ν [a] /cm^{-1}	intensity[b]
ν_1	3150	2.2	3144	3.5	3145	3.6
ν_2	3066	3.8	3062	5.4	3063	5.3
ν_3	3034	0.5	3033	0.0	3030	10.1
ν_4	3030	13.1	3028	11.1	3020	1.1
ν_5	2988	8.5	2989	9.2	2994	9.2
ν_6	2940	15.7	2940	16.2	2942	19.2
ν_7	1651	4.0	1673	7.8	1673	8.8
ν_8	1461	16.6	1460	16.9	1460	16.9
ν_9	1445	9.6	1444	9.4	1446	10.1
ν_{10}	1419	4.9	1413	6.6	1412	6.3
ν_{11}	1383	7.6	1380	3.2	1382	3.6
ν_{12}	1346	4.0	1289	15.0	1314	10.6
ν_{13}	1233	0.4	1230	8.1	1231	3.7
ν_{14}	1142	33.3	1160	30.5	1159	28.6
ν_{15}	1092	13.6	1135	8.5	1093	7.0
ν_{16}	1046	3.0	1052	2.9	1052	3.4
ν_{17}	1013	1.4	1005	5.9	1007	6.6
ν_{18}	967	10.1	959	18.3	961	11.9
ν_{19}	940	37.9	955	26.1	949	28.0
ν_{20}	901	19.2	885	32.9	877	1.7
ν_{21}	822	40.1	854	16.0	815	46.4
ν_{22}	745	34.6	702	10.7	739	30.5
ν_{23}	670	4.0	659	40.6	648	4.8
ν_{24}	559	33.7	539	7.4	594	24.2
ν_{25}	475	5.4	486	9.0	458	2.9
ν_{26}	375	4.4	410	5.2	433	3.7
ν_{27}	338	7.6	292	1.9	294	3.4
ν_{28}	287	0.5	256	1.3	267	2.2
ν_{29}	208	1.0	233	0.8	214	1.4
ν_{30}	173	0.3	181	0.9	179	0.8
ν_{31}	152	1.4	177	0.2	176	0.7
ν_{32}	129	0.0	71	1.4	92	0.6
ν_{33}	69	0.2	44	0.2	50	0.1

[a]Harmonic vibrational wavenumber x scaled according to $y = (0.9683 \pm 0.0116)\, x + (11.5 \pm 13.8)$; see text.

[b]In unit km mol^{-1}.

Supplementary Table 9 Vibrational wavenumbers and IR intensities of two conformers of iodoperoxy radical 3-hydroperoxy-1-iodo-2-methyl-prop-1-ene (CHI)C(CH_3)CH_2OO, (5a) and (5b), predicted with the B3LYP/aug-cc-pVTZ-pp method.

mode	(CHI)C(CH_3)CH_2OO **(5a)**		(CHI)C(CH_3)CH_2OO **(5b)**	
	ν [a] /cm^{-1}	intensity[b]	ν [a] /cm^{-1}	intensity[b]
ν_1	3110	6.0	3109	5.6
ν_2	3043	5.2	3042	5.0
ν_3	3028	8.9	3040	5.5
ν_4	2987	6.9	2990	6.5
ν_5	2968	10.2	2978	13.2
ν_6	2941	13.2	2943	10.5
ν_7	1631	22.4	1625	23.1
ν_8	1453	10.3	1449	9.0
ν_9	1450	1.9	1444	1.5
ν_{10}	1441	4.9	1433	7.1
ν_{11}	1385	9.0	1388	8.8
ν_{12}	1332	49.9	1320	11.4
ν_{13}	1280	74.4	1279	91.4
ν_{14}	1192	8.4	1242	5.4
ν_{15}	1153	16.6	1145	5.5
ν_{16}	1147	2.6	1128	3.5
ν_{17}	1049	2.0	1054	10.1
ν_{18}	1034	29.0	1037	22.3
ν_{19}	950	0.3	958	3.3
ν_{20}	889	17.3	854	12.4
ν_{21}	842	3.3	828	3.8
ν_{22}	797	31.0	787	29.1
ν_{23}	667	16.8	663	23.7
ν_{24}	553	17.8	574	9.8
ν_{25}	476	10.7	483	8.2
ν_{26}	387	2.1	424	3.8
ν_{27}	287	2.4	308	1.7
ν_{28}	255	0.4	243	0.6
ν_{29}	194	0.5	203	1.2
ν_{30}	148	2.0	160	1.1
ν_{31}	114	0.6	121	0.4
ν_{32}	74	1.1	84	2.3
ν_{33}	53	2.9	54	2.4

[a]Harmonic vibrational wavenumber x scaled according to $y = (0.9683 \pm 0.0116)\, x + (11.5 \pm 13.8)$; see text.

[b]In unit km mol^{-1}.

Supplementary Table 10 Rotational parameters and ratios of types for each vibrational state of four conformers of Carbonyl oxides MACRO (3) and dioxole predicted with the B3LYP/ aug-cc-pVTZ method.

mode	sym.	*anti-trans*-MACRO **(3a)**					
		A'/*A*"	*B*'/*B*"	*C*'/*C*"	type ratio		
					a	*b*	*c*
		A"=0.2796 cm^{-1}, B"=0.0715 cm^{-1}, C"=0.0575 cm^{-1}					
ν_1	a'	0.99942	0.99975	0.99972	0.02	0.98	0.00
ν_2	a'	0.99921	0.99908	0.99911	0.25	0.75	0.00
ν_3	a'	0.99936	0.99980	0.99972	0.81	0.19	0.00
ν_4	a'	0.99967	0.99976	0.99981	0.52	0.48	0.00
ν_5	a'	0.99969	0.99973	0.99986	0.05	0.95	0.00
ν_6	a'	0.99770	0.99866	0.99851	0.16	0.84	0.00
ν_7	a'	1.00058	0.99848	0.99884	0.90	0.10	0.00
ν_8	a'	1.00029	0.99958	1.00071	0.83	0.17	0.00
ν_9	a'	0.99932	0.99943	0.99950	0.45	0.55	0.00
ν_{10}	a'	0.99477	0.99958	0.99833	0.81	0.19	0.00
ν_{11}	a'	0.99879	0.99842	0.99804	0.02	0.98	0.00
ν_{12}	a'	1.00000	1.00055	0.99967	0.27	0.73	0.00
ν_{13}	a'	0.99892	0.99982	0.99934	0.99	0.01	0.00
ν_{14}	a'	1.00270	1.00045	0.99932	0.80	0.20	0.00
ν_{15}	a'	0.99881	0.99690	0.99699	0.93	0.07	0.00
ν_{16}	a'	0.99670	0.99943	0.99871	0.38	0.62	0.00
ν_{17}	a'	1.00047	0.99973	0.99941	0.63	0.37	0.00
ν_{18}	a'	0.99777	0.99966	0.99974	1.00	0.00	0.00
ν_{19}	a'	1.00090	1.00052	0.99953	0.11	0.89	0.00
ν_{20}	a'	1.00489	0.99965	0.99927	0.06	0.94	0.00
ν_{21}	a"	0.99995	0.99976	0.99986	0.00	0.00	1.00
ν_{22}	a"	1.00595	0.99994	1.00003	0.00	0.00	1.00
ν_{23}	a"	0.99816	1.00000	0.99981	0.00	0.00	1.00
ν_{24}	a"	0.99963	0.99897	1.00036	0.00	0.00	1.00
ν_{25}	a"	0.99704	0.99982	1.00038	0.00	0.00	1.00
ν_{26}	a"	0.99935	0.99966	1.00010	0.00	0.00	1.00
ν_{27}	a"	1.00465	0.99969	1.00043	0.00	0.00	1.00
ν_{28}	a"	0.99898	1.00036	1.00042	0.00	0.00	1.00
ν_{29}	a"	0.99698	1.00049	1.00104	0.00	0.00	1.00
ν_{30}	a"	0.99467	0.99894	0.99927	0.00	0.00	1.00
mode	sym.	*syn-cis*-MACRO **(3b)**					
		A'/*A*"	*B*'/*B*"	*C*'/*C*"	type ratio		
					a	*b*	*c*
		A"=0.2571 cm^{-1}, B"=0.0846 cm^{-1}, C"=0.0644 cm^{-1}					
ν_1	a'	1.00000	0.99789	0.99843	0.21	0.79	0.00
ν_2	a'	0.99916	0.99933	0.99932	0.62	0.38	0.00
ν_3	a'	0.99948	0.99993	0.99983	0.25	0.75	0.00
ν_4	a'	0.99991	0.99976	0.99984	0.51	0.49	0.00
ν_5	a'	0.99935	1.00005	1.00003	0.46	0.54	0.00
ν_6	a'	0.99761	1.00012	0.99971	0.00	1.00	0.00

ν_7	a'	0.99935	0.99777	0.99777	0.78	0.22	0.00
ν_8	a'	0.99959	1.00002	1.00099	0.70	0.30	0.00
ν_9	a'	0.99998	0.99889	0.99926	0.43	0.57	0.00
ν_{10}	a'	1.00010	0.99939	0.99918	0.98	0.02	0.00
ν_{11}	a'	0.99950	0.99987	0.99946	0.74	0.26	0.00
ν_{12}	a'	0.99858	0.99907	0.99806	0.30	0.70	0.00
ν_{13}	a'	0.99797	1.00057	0.99955	0.00	1.00	0.00
ν_{14}	a'	0.99882	0.99922	0.99870	0.21	0.79	0.00
ν_{15}	a'	0.99946	0.99879	0.99877	0.03	0.97	0.00
ν_{16}	a'	0.99989	0.99844	0.99856	0.30	0.70	0.00
ν_{17}	a'	1.00022	0.99885	0.99857	0.44	0.56	0.00
ν_{18}	a'	0.99981	0.99959	0.99974	0.27	0.73	0.00
ν_{19}	a'	1.00183	1.00026	0.99932	0.01	0.99	0.00
ν_{20}	a'	1.00240	0.99809	0.99727	0.22	0.78	0.00
ν_{21}	a"	0.99940	1.00024	1.00009	0.00	0.00	1.00
ν_{22}	a"	1.00046	1.00142	1.00003	0.00	0.00	1.00
ν_{23}	a"	1.00175	0.99915	0.99963	0.00	0.00	1.00
ν_{24}	a"	0.99776	0.99963	1.00020	0.00	0.00	1.00
ν_{25}	a"	0.99957	0.99948	1.00011	0.00	0.00	1.00
ν_{26}	a"	0.99793	1.00017	1.00040	0.00	0.00	1.00
ν_{27}	a"	0.99947	1.00047	1.00068	0.00	0.00	1.00
ν_{28}	a"	0.99995	1.00106	1.00158	0.00	0.00	1.00
ν_{29}	a"	1.00032	0.99923	1.00002	0.00	0.00	1.00
ν_{30}	a"	0.99589	0.99992	1.00168	0.00	0.00	1.00

mode	sym.	*syn-trans*-MACRO **(3c)**					
		A'/*A*"	*B*'/*B*"	*C*'/*C*"	type ratio		
					a	*b*	*c*
		A''=0.2290 cm^{-1}, B''=0.0911 cm^{-1}, C''=0.0660 cm^{-1}					
ν_1	a'	0.99956	0.99968	0.99968	0.98	0.02	0.00
ν_2	a'	0.99910	0.99925	0.99923	0.46	0.54	0.00
ν_3	a'	0.99950	0.99975	0.99967	0.34	0.66	0.00
ν_4	a'	0.99965	0.99979	0.99988	0.64	0.36	0.00
ν_5	a'	0.99966	0.99996	0.99995	0.47	0.53	0.00
ν_6	a'	0.99903	0.99866	0.99877	1.00	0.00	0.00
ν_7	a'	1.00015	0.99740	0.99805	0.30	0.70	0.00
ν_8	a'	1.00066	0.99956	1.00071	0.11	0.89	0.00
ν_9	a'	0.99815	0.99939	0.99964	0.79	0.21	0.00
ν_{10}	a'	0.99401	1.00003	0.99892	0.71	0.29	0.00
ν_{11}	a'	0.99960	1.00052	1.00006	0.45	0.55	0.00
ν_{12}	a'	0.99839	0.99883	0.99723	0.03	0.97	0.00
ν_{13}	a'	0.99905	0.99986	0.99929	0.00	1.00	0.00
ν_{14}	a'	1.00387	1.00005	0.99920	0.01	0.99	0.00
ν_{15}	a'	0.99772	0.99978	0.99912	0.04	0.96	0.00
ν_{16}	a'	0.99768	0.99906	0.99865	0.34	0.66	0.00
ν_{17}	a'	1.00000	0.99912	0.99845	0.13	0.87	0.00
ν_{18}	a'	1.00293	0.99959	1.00021	0.45	0.55	0.00
ν_{19}	a'	1.00208	1.00004	0.99921	0.57	0.43	0.00
ν_{20}	a'	1.00161	0.99777	0.99677	0.33	0.67	0.00
ν_{21}	a"	1.00021	0.99895	0.99935	0.00	0.00	1.00

ν_{22}	a"	1.00727	1.00037	1.00020	0.00	0.00	1.00
ν_{23}	a"	0.99826	1.00003	0.99979	0.00	0.00	1.00
ν_{24}	a"	0.99674	0.99894	1.00015	0.00	0.00	1.00
ν_{25}	a"	0.99981	0.99934	1.00009	0.00	0.00	1.00
ν_{26}	a"	0.99923	0.99928	1.00008	0.00	0.00	1.00
ν_{27}	a"	0.99900	1.00010	1.00033	0.00	0.00	1.00
ν_{28}	a"	0.99821	1.00091	1.00139	0.00	0.00	1.00
ν_{29}	a"	0.99866	0.99617	0.99709	0.00	0.00	1.00
ν_{30}	a"	0.99552	1.00089	1.00380	0.00	0.00	1.00
mode	sym.	*anti-cis*-MACRO **(3d)**					
		A'/*A*"	*B*'/*B*"	*C*'/*C*"	type ratio		
					a	*b*	*c*
		A"=0.2958 cm^{-1}, *B*"=0.0681 cm^{-1}, *C*"=0.0559 cm^{-1}					
ν_{1}	a'	0.99889	0.99976	0.99964	0.49	0.51	0.00
ν_{2}	a'	0.99915	0.99975	0.99964	0.03	0.97	0.00
ν_{3}	a'	0.99914	0.99929	0.99927	0.01	0.99	0.00
ν_{4}	a'	1.00002	0.99974	0.99984	0.13	0.87	0.00
ν_{5}	a'	0.99940	1.00004	1.00005	0.96	0.04	0.00
ν_{6}	a'	0.99738	0.99944	0.99912	0.74	0.26	0.00
ν_{7}	a'	1.00018	0.99762	0.99791	0.97	0.03	0.00
ν_{8}	a'	0.99976	1.00004	1.00068	0.01	0.99	0.00
ν_{9}	a'	1.00002	0.99937	1.00023	0.02	0.98	0.00
ν_{10}	a'	0.99711	0.99912	0.99803	0.83	0.17	0.00
ν_{11}	a'	1.00050	0.99871	0.99887	0.01	0.99	0.00
ν_{12}	a'	0.99884	0.99928	0.99835	0.18	0.82	0.00
ν_{13}	a'	0.99749	0.99972	0.99927	0.62	0.38	0.00
ν_{14}	a'	0.99947	0.99906	0.99861	0.05	0.95	0.00
ν_{15}	a'	0.99871	0.99812	0.99787	0.69	0.31	0.00
ν_{16}	a'	0.99831	0.99835	0.99830	0.50	0.50	0.00
ν_{17}	a'	0.99918	0.99990	0.99950	0.44	0.56	0.00
ν_{18}	a'	0.99905	0.99935	0.99954	0.07	0.93	0.00
ν_{19}	a'	0.99901	1.00073	0.99950	0.20	0.80	0.00
ν_{20}	a'	1.00356	1.00098	0.99945	0.11	0.89	0.00
ν_{21}	a"	0.99955	1.00019	1.00013	0.00	0.00	1.00
ν_{22}	a"	1.00279	1.00082	1.00004	0.00	0.00	1.00
ν_{23}	a"	1.00112	0.99968	0.99973	0.00	0.00	1.00
ν_{24}	a"	0.99993	0.99944	1.00023	0.00	0.00	1.00
ν_{25}	a"	0.99817	0.99999	1.00032	0.00	0.00	1.00
ν_{26}	a"	0.99913	0.99999	1.00020	0.00	0.00	1.00
ν_{27}	a"	1.00422	0.99984	1.00055	0.00	0.00	1.00
ν_{28}	a"	0.99742	1.00073	1.00148	0.00	0.00	1.00
ν_{29}	a"	0.99971	0.99919	0.99964	0.00	0.00	1.00
ν_{30}	a"	0.99798	1.00013	1.00256	0.00	0.00	1.00
mode	sym.	dioxole					
		A'/*A*"	*B*'/*B*"	*C*'/*C*"	type ratio		
					a	*b*	*c*
		A"=0.2597 cm^{-1}, *B*"=0.1069 cm^{-1}, *C*"=0.0781 cm^{-1}					
ν_{1}	a	0.99868	0.99960	0.99933	0.60	0.40	0.00
ν_{2}	a	0.99961	0.99989	0.99987	0.05	0.95	0.00

v_3	a	0.99958	1.00042	1.00026	0.00	0.00	1.00
v_4	a	0.99945	1.00016	1.00014	0.85	0.15	0.00
v_5	a	0.99990	1.00084	1.00063	0.00	0.00	1.00
v_6	a	0.99952	1.00055	1.00049	0.08	0.92	0.00
v_7	a	0.99835	0.99804	0.99825	0.78	0.22	0.00
v_8	a	0.99928	1.00005	1.00092	0.80	0.20	0.00
v_9	a	0.99955	1.00015	1.00152	0.16	0.84	0.00
v_{10}	a	0.99963	1.00278	1.00018	0.00	0.00	1.00
v_{11}	a	1.00019	0.99666	0.99798	0.82	0.18	0.00
v_{12}	a	0.99911	0.99907	0.99788	0.84	0.16	0.00
v_{13}	a	0.99996	0.99997	0.99928	0.96	0.04	0.00
v_{14}	a	0.99945	0.99794	0.99742	0.89	0.11	0.00
v_{15}	a	0.99926	0.99927	1.00100	0.00	0.00	1.00
v_{16}	a	0.99936	0.99903	0.99922	0.92	0.08	0.00
v_{17}	a	0.99995	0.99914	0.99895	0.00	0.00	1.00
v_{18}	a	0.99922	0.99903	0.99864	0.97	0.03	0.00
v_{19}	a	1.00089	0.99991	0.99895	0.00	0.00	1.00
v_{20}	a	0.99887	0.99748	1.00090	0.19	0.81	0.00
v_{21}	a	0.99824	0.99916	0.99680	1.00	0.00	0.00
v_{22}	a	0.99886	0.99986	1.00029	0.00	0.00	1.00
v_{23}	a	0.99639	1.00031	1.00061	1.00	0.00	0.00
v_{24}	a	0.99946	0.99942	0.99605	0.46	0.54	0.00
v_{25}	a	0.99980	0.99950	0.99890	0.99	0.01	0.00
v_{26}	a	0.99922	0.99939	1.00050	0.00	0.00	1.00
v_{27}	a	1.01116	1.00107	0.99971	0.44	0.56	0.00
v_{28}	a	0.99011	1.00078	1.00155	0.00	0.00	1.00
v_{29}	a	0.99970	0.99901	0.99945	0.00	0.00	1.00
v_{30}	a	1.00236	1.00007	1.00667	0.00	0.00	1.00

Supplementary Table 11 Summary of estimates of relative abundance of species (3)−(5) observed under varied pressures.

	Description	Unit	Expt. 1	Expt. 2	Expt. 3	Expt. 4
	**Method I**					
1	Pressure of $CH_2IC(CH_3)CHI$ **(1)**	mTorr	32	57	55	55
2	Pressure of O_2	Torr	21.0	86.0	229	346
3	Probed period	μs	0−5	0−5	0−5	0−5
4	Integrated abs. (1272−1301 cm^{-1}) for loss of **(1)**	10^{-3} cm^{-1}	100	96	80	85
5	Integrated abs., B_1 (895−925 cm^{-1}) for **(3),** C_3 stripped		35	46	36	41
6	Subtracted factor for group C **(4)**		1.44	2.02	2.03	2.34
7	Subtraction factor for group D **(5)**		0.87	0.97	1.00	1.20
8	Relative **(3),** from row 5, normalize to Expt. 1		1	1.37	1.29	1.38
9	Relative **(4),** from row 6, normalize to Expt. 1		1	1.46	1.76	1.91
10	Relative **(5),** from row 7, normalize to Expt. 1		1	1.16	1.43	1.62
	**Method II**					
11	Integrated abs., B_1/C_3 (895−925 cm^{-1}) for **(3)/(4)**	10^{-3} cm^{-1}	53	69	61	74
12	Integrated abs. C_4 (880-895 cm^{-1}) for **(4)**	10^{-3} cm^{-1}	15	20	18	23
13	Integrated abs., C_1 (1083−1138 cm^{-1}) for **(4)**	10^{-3} cm^{-1}	14	24	38	51
14	Calculated abs., D_1 (1324−1344 cm^{-1}) for **(5)**	10^{-3} cm^{-1}	11	13	13	16
15	Estimated abs., C_3 for **(4)** from C_4, line 12 (Method II-C_4)	10^{-3} cm^{-1}	8	11	10	13
16	Estimated abs., C_3 for **(4)** from C_1, line 13 (Method II-C_1)	10^{-3} cm^{-1}	13	22	34	46
17	Estimated abs. B_1 for **(3)**, from lines 11 & 15 (by C_4)	10^{-3} cm^{-1}	45	58	51	61
18	Estimated abs. B_1 for **(3)**, from line's 11 & 16 (by C_1)	10^{-3} cm^{-1}	40	47	27	28
19	Reletive **(3),** from row 17, normalize to Expt. 1 (Method II-C_4)		1	1.35	1.43	1.61
20	Reletive **(3),** from row 18, normalize to Expt. 1 (Method II-C_1)		1	1.22	0.83	0.82
21	Reletive **(4),** from row 13, normalized to Expt. 1 (Method II-C_4)		1	1.39	1.50	1.80
22	Reletive **(4),** from row 12, normalized to Expt. 1 (Method II-C_1)		1	1.79	3.39	4.29
23	Loss of **(1),** from three bands	mTorr	3.44	3.30	2.75	2.93
24	Formation of **(3)**, from line 5	mTorr	0.20	0.27	0.21	0.24
25	percentage yield of **(3)**, from lines 23 & 24		5.8	8.2	7.6	8.2

(a) (E)-$CH_2IC(CH_3)CHI$ **(1a)**

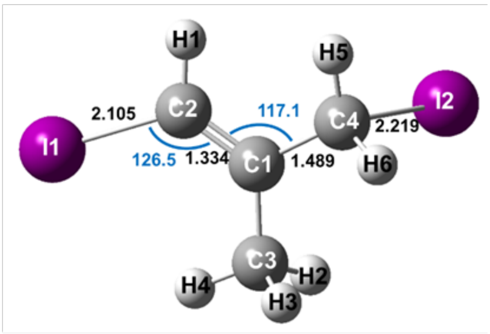

1.6 kJ mol^{-1}

(b) (Z)-$CH_2IC(CH_3)CHI$ **(1b)**

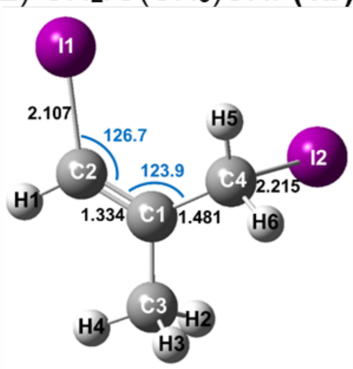

0.0 kJ mol^{-1}

(c) (E)-$CH_2C(CH_3)CHI$ **(2a)**

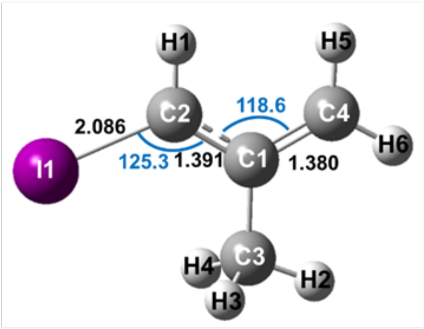

0.0 kJ mol^{-1}

(d) (Z)-$CH_2C(CH_3)CHI$ **(2b)**

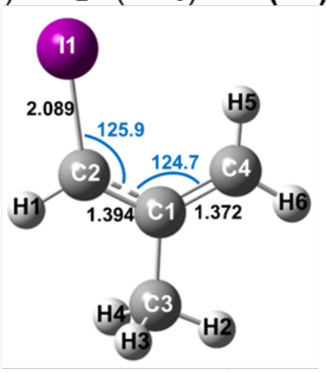

1.5 kJ mol^{-1}

(e) (E)-$CH_2IC(CH_3)CH$ **(6a)**

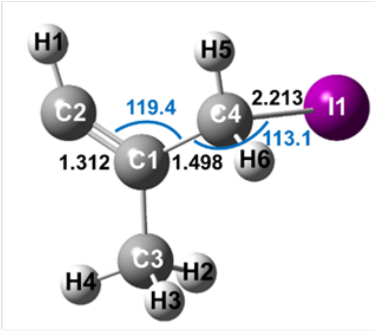

100 kJ mol^{-1}

(f) (Z)-$CH_2IC(CH_3)CH$ **(6b)**

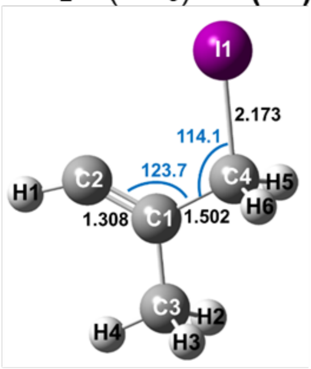

106 kJ mol^{-1}

Supplementary Fig. 1: Geometries of conformers of precursor $CH_2IC(CH_3)CHI$ (1), iodoalkenyl radicals $CH_2C(CH_3)CHI$ (2) and $CH_2IC(CH_3)CH$ (6) predicted with the B3LYP/aug-cc-pVTZ-pp method. a (E)-$CH_2IC(CH_3)CHI$ **(1a)**. **b** (Z)-$CH_2IC(CH_3)CHI$ **(1b)**. **c** (E)-$CH_2C(CH_3)CHI$ **(2a)**. **d** (Z)-$CH_2C(CH_3)CHI$ **(2b). e** (E)-$CH_2IC(CH_3)CH$ **(6a)**. **f** (Z)-$CH_2IC(CH_3)CH$ **(6b)**. Bond lengths (black) are in Å and angles (blue) are in degree. The ZVPE-corrected relative energies (in kJ mol^{-1}), computed with the B3LYP/aug-cc-pVTZ-pp method, are shown for conformers of each species.

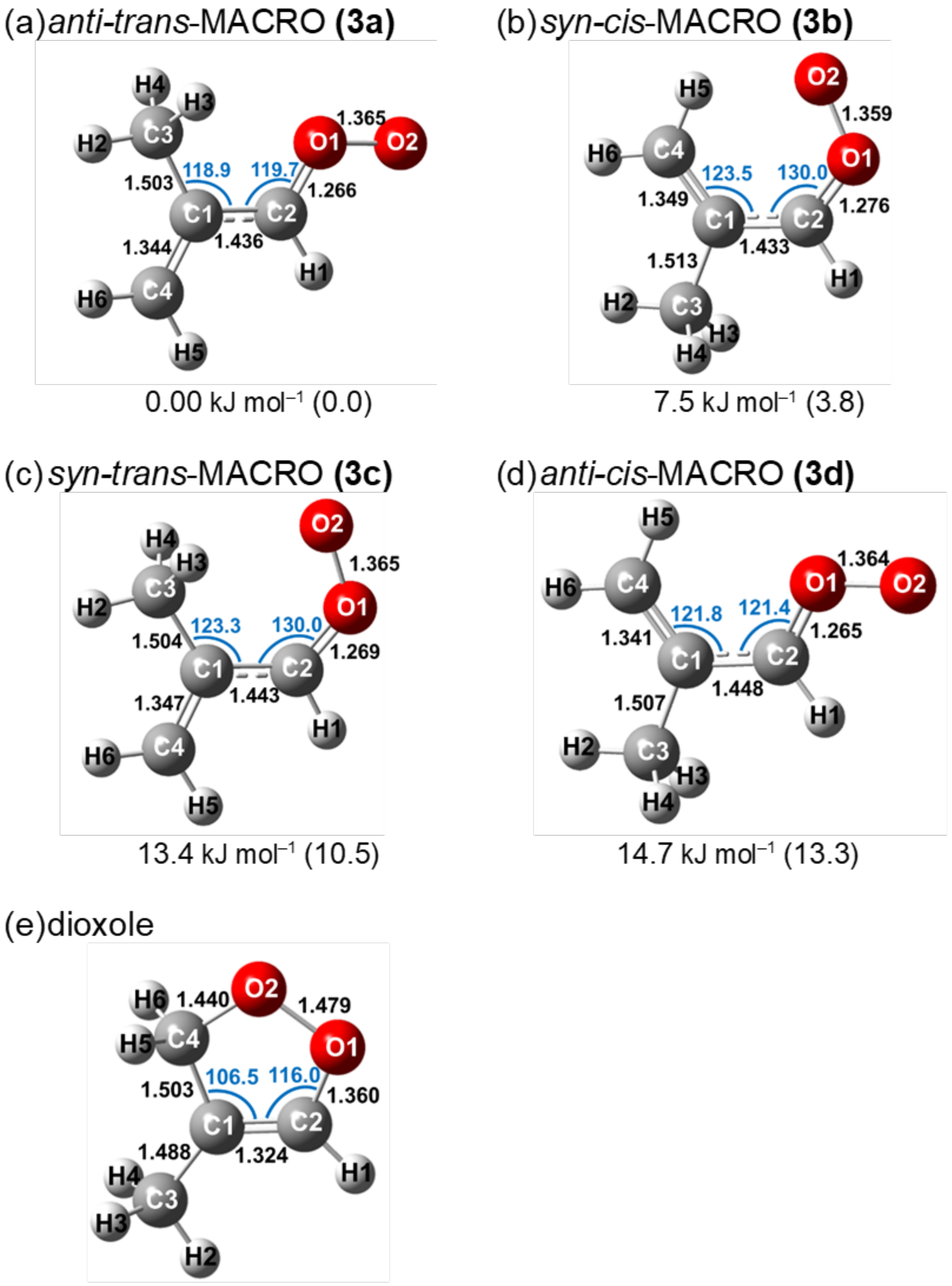

Supplementary Fig. 2: Geometries of conformers of methacrolein oxide (MACRO) and dioxole predicted with the B3LYP/aug-cc-pVTZ method. **a** *anti-trans*-MACRO **(3a)**. **b** *syn-cis*-MACRO **(3b)**. **c** *syn-trans*-MACRO **(3c)**. **d** *anti-cis*-MACRO **(3d)**. **e** dioxole. Bond lengths (black) are in Å and angles (blue) are in degree. The ZVPE-corrected relative energies (in kJ mol^{-1}), computed with the B3LYP/aug-cc-pVTZ method, are shown for conformers of each species; those of CCSD(T)-F12/CBS(TZ-F12,QZ-F12)//B2PLYP-D3/cc-pVTZ, reported by Vansco *et al*. in reference 1, are listed in parentheses.

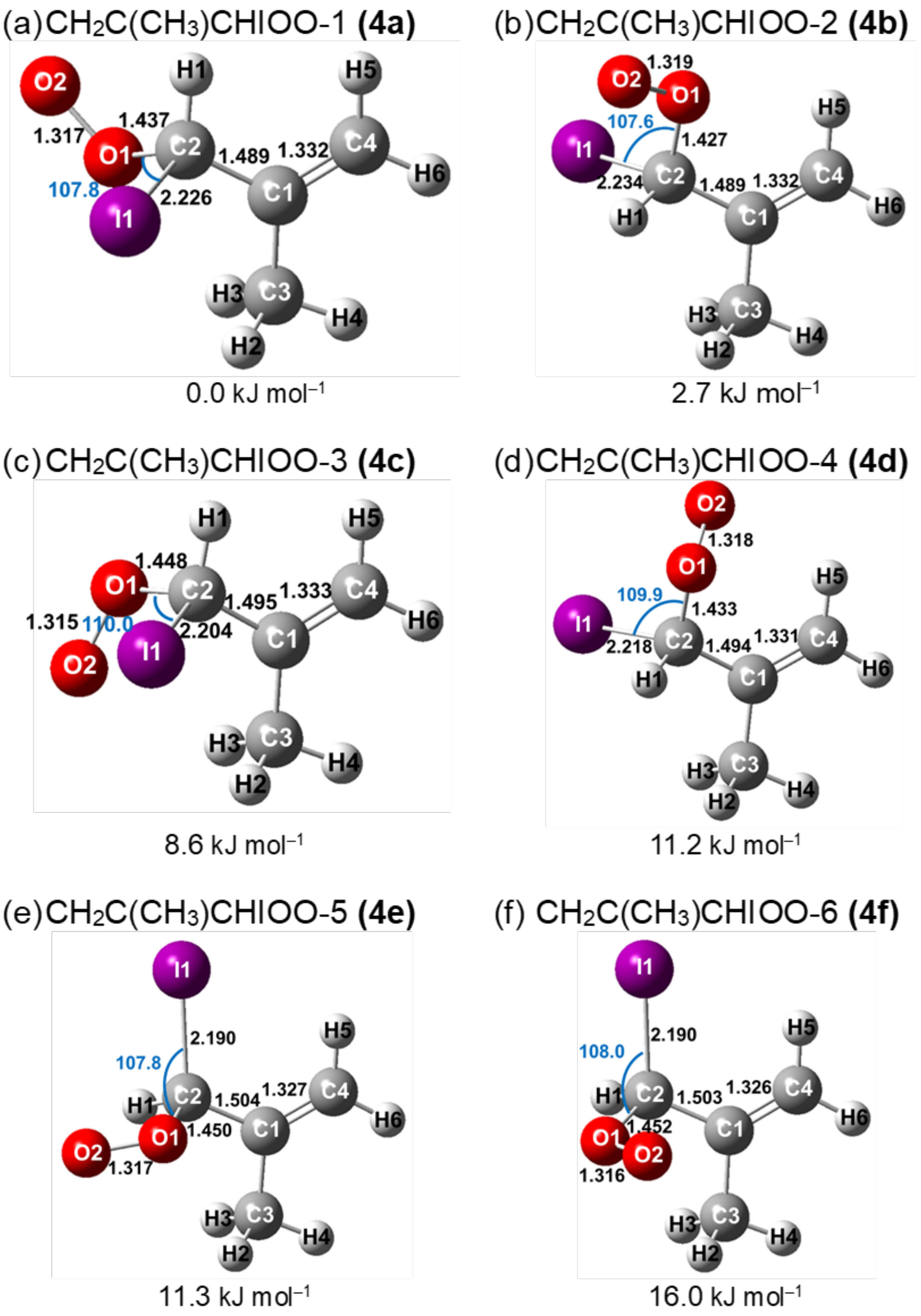

Supplementary Fig. 3: Geometries of six conformers of iodoperoxy radicals 3-hydroperoxy-3-iodo-2- methyl-prop-1-ene $CH_2C(CH_3)CHIOO$ (4) predicted with the B3LYP/aug-cc-pVTZ-pp method. Bond lengths (black) are in Å and angles (blue) are in degree. The ZPE-corrected relative energies (in kJ mol^{-1}), computed with the B3LYP/aug-cc-pVTZ-pp method, are shown.

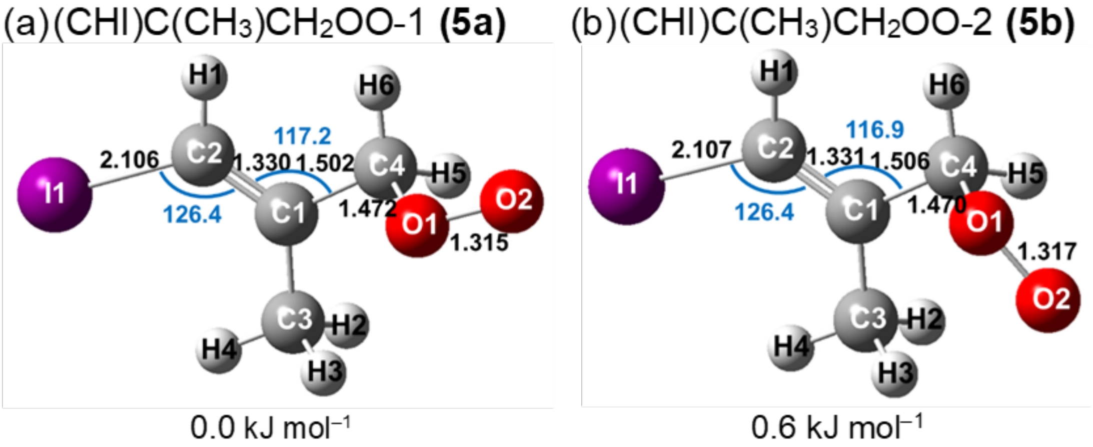

Supplementary Fig. 4: Geometries of two conformers of 3-hydroperoxy-1-iodo-2-methyl-prop-1-ene (CHI)C(CH_3)CH_2OO (5) predicted with the B3LYP/aug-cc-pVTZ-pp method. Bond lengths (black) are in Å and angles (blue) are in degree. The ZPE-corrected relative energies (in kJ mol^{-1}), computed with the B3LYP/aug-cc-pVTZ-pp method, are shown.

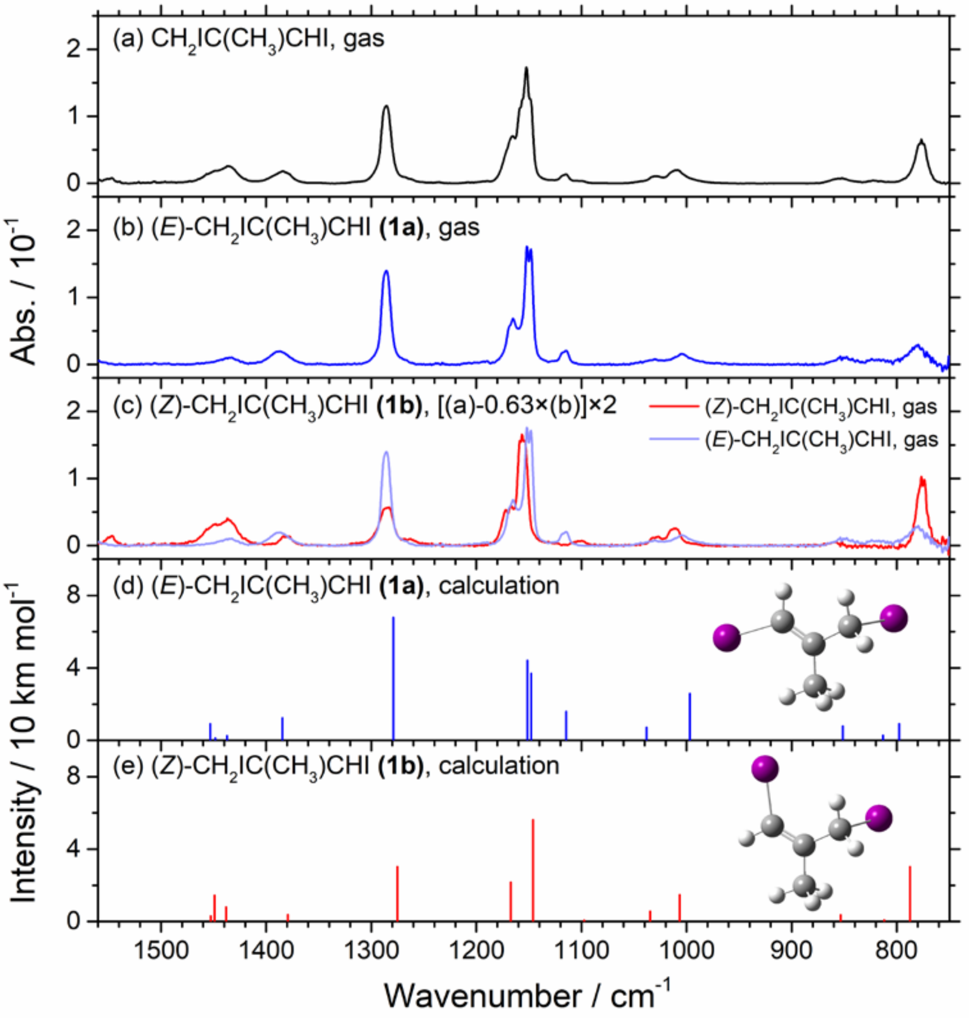

Supplementary Fig. 5: Comparison of IR spectra of gaseous (*E*)-/(*Z*)-$CH_2IC(CH_3)CHI$, (1a) and (1b), with predicted IR stick spectra. a Spectrum of a mixture of (*E*)-/(*Z*)-conformers of $CH_2IC(CH_3)CHI$ **(1)**. **b** Spectrum of (*E*)-$CH_2IC(CH_3)CHI$ **(1a)**. **c** Spectrum of (*Z*)-$CH_2IC(CH_3)CHI$ **(1b)** (red) derived by subtracting 0.63 times spectrum **b** from spectrum **a**. Spectrum of **(1a)** is shown in grey for comparison; both spectra are normalized. IR stick spectra according to scaled harmonic vibrational wavenumbers and IR intensities predicted with the B3LYP/aug-cc-pVTZ-pp method are shown for **(1a)** and **(1b)** in **f** and **g**, respectively.

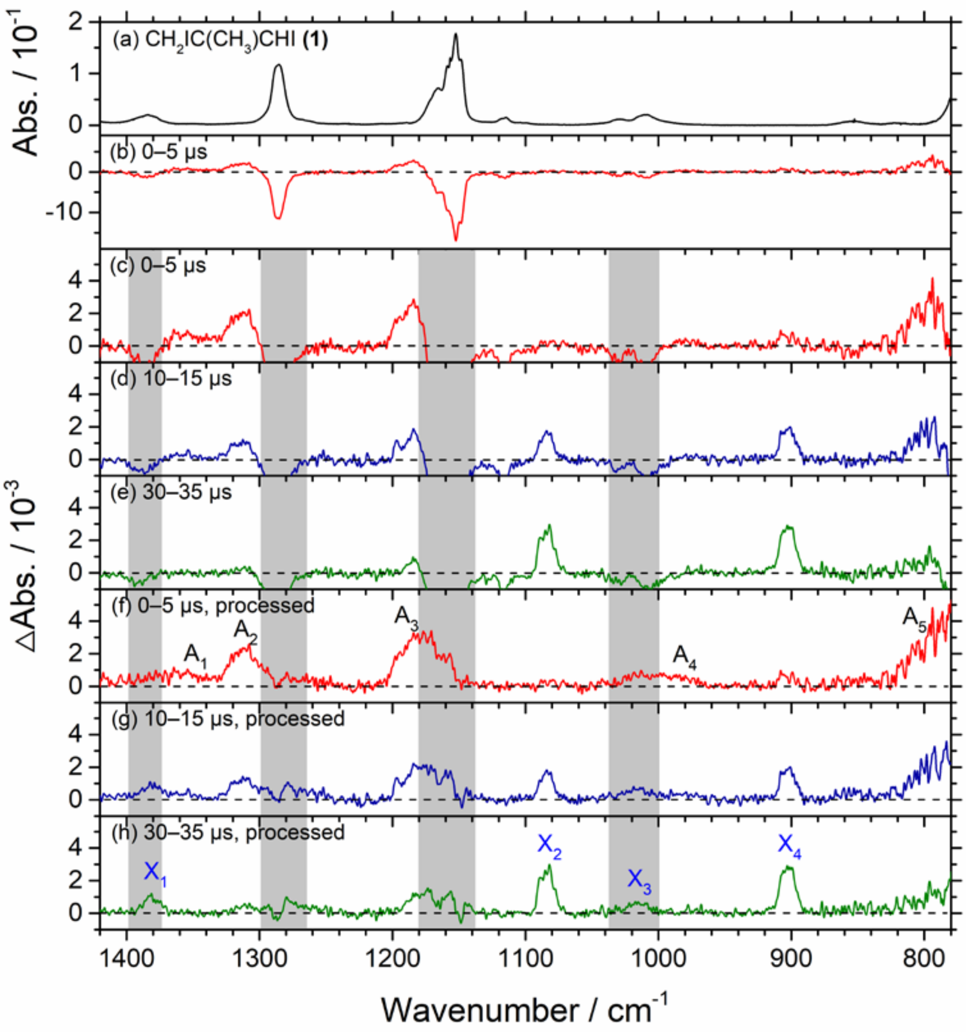

Supplementary Fig. 6: Observed and processed spectra in region 1420−780 cm^{-1} upon photolysis at 248 nm of a flowing mixture of $CH_2IC(CH_3)CHI$ (1)/O_2 (0.030/20.0 Torr). a Absorption spectrum before photolysis. **b** Difference spectra recorded 0−5 μs after photolysis. **c**−**e** Expanded difference spectra recorded 0−5, 10−15 and 30−35 μs after irradiation; negative bands are truncated. **f**−**h** Processed spectra of **c**–**e** with absorption bands of precursor **(1)**, spectrum **a**, added back to eliminate negative bands. Grey areas represent regions of possible interference from absorption of the parent molecule. New features with decreasing intensity are marked A_1−A_5 in **f**. New features with increasing intensity are marked X_1−X_4 in **h**. Spectral resolution is 1.0 cm^{-1}.

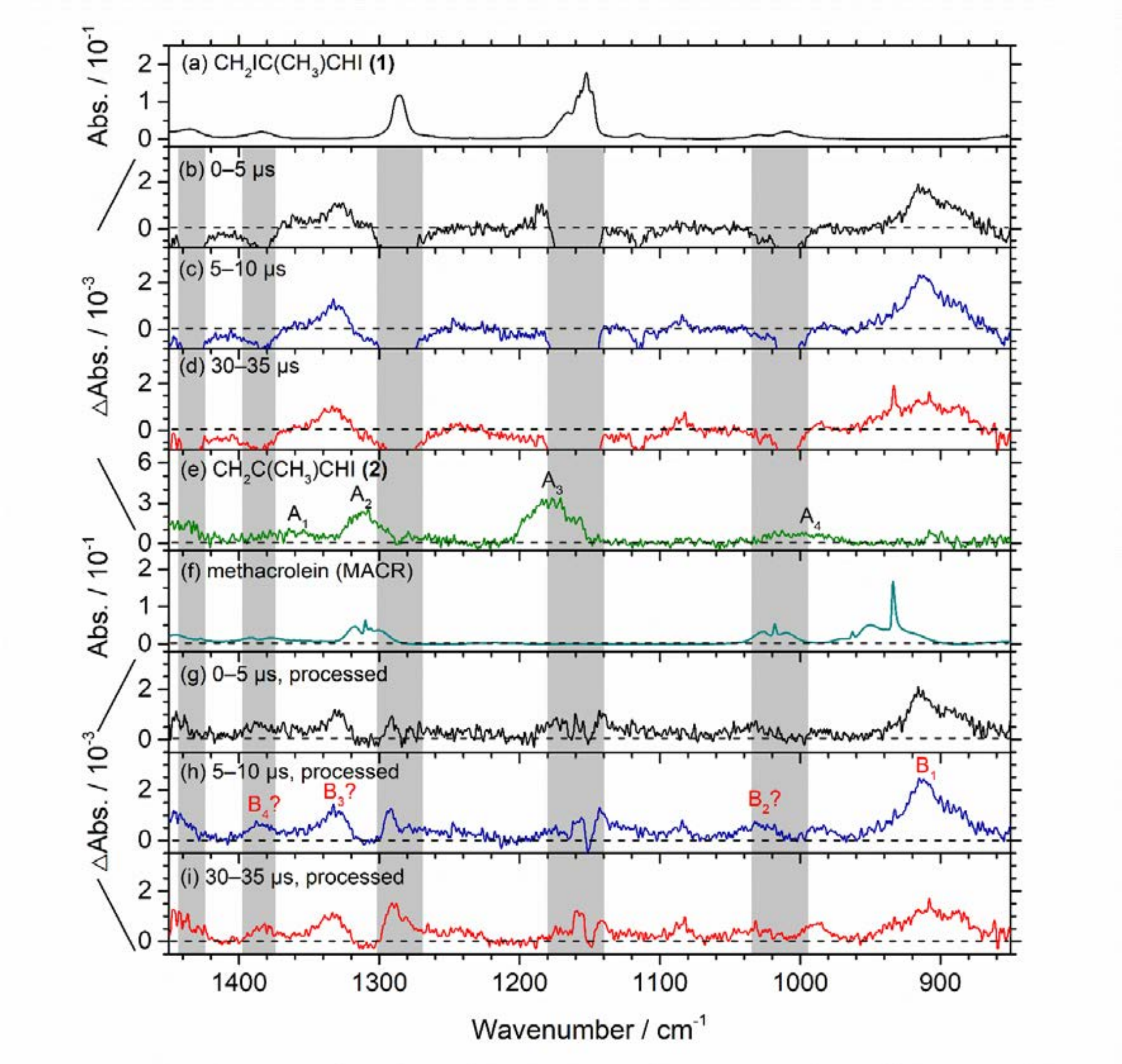

Supplementary Fig. 7: Temporal evolution of observed and processed spectra in region 1450–850 cm^{-1} on photolysis at 248 nm of a flowing mixture of $CH_2IC(CH_3)CHI$ (1)/O_2 (0.030/21.0 Torr). a Absorption spectrum before photolysis. Difference spectra recorded 0–5 (**b**), 5–10 (**c**), and 30–35 (**d**) μs after irradiation. **e** Spectrum of $CH_2C(CH_3)CHI$ **(2)** taken from Supplementary Fig. 6f. **f** Absorption spectrum of methacrolein (MACR). **g**–**i** Processed spectra of **b**–**d** with bands of **(2)** and MACR removed and those of the precursor **(1)** added back. Grey areas represent regions of possible interference from absorption of the parent molecules. New features are marked B_1−B_4 in **h**; B_1 and B_4 are uncertain because of the interference from parent absorption. Spectral resolution is 1.0 cm^{-1}.

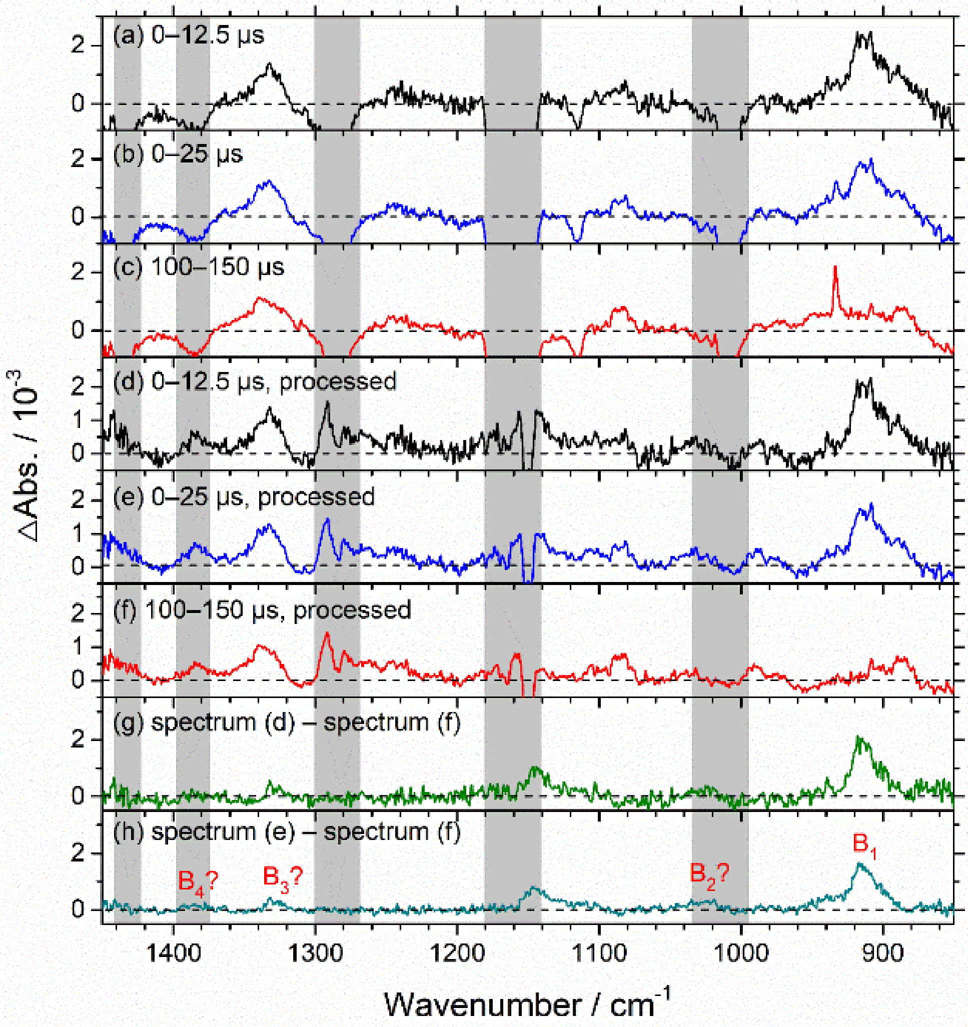

Supplementary Fig. 8: Observed and processed spectra in region 1450−850 cm^{-1} upon photolysis at 248 nm of a flowing mixture of $CH_2IC(CH_3)CHI$ (1)/O_2 (0.030/20.0 Torr). Difference spectra recorded 0−12.5 (**a**), 0−25 (**b**), and 100−150 (**c**) μs after irradiation; negative bands are truncated. **d**−**f** Processed spectra of **a**−**c** with bands of $CH_2C(CH_3)CHI$ **(2)** and methacrolein (MACR) removed and those of the precursor **(1)** added back. Grey areas represent regions of possible interference from absorption of the parent molecules. **g** Spectrum **d** subtracts spectrum **f**. **h** Spectrum **e** subtracts spectrum **f**. New features are marked B_1−B_4 in **h**; the latter three are uncertain because of their small intensities. Spectral resolution is 1.0 cm^{-1}.

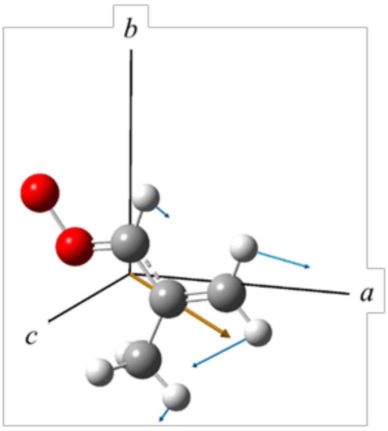

ν_{14}, 985 cm^{-1}

a : *b* : *c* = 0.80 : 0.20 : 0.00

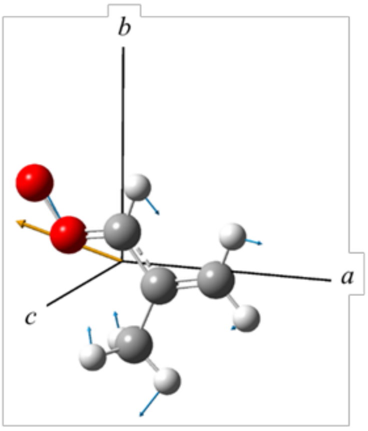

ν_{15}, 944 cm^{-1}

a : *b* : *c* = 0.93 : 0.07 : 0.00

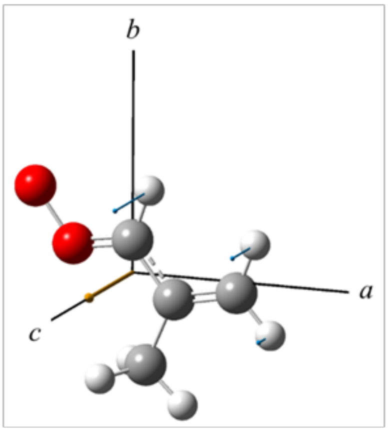

ν_{24}, 950 cm^{-1}

a : *b* : *c* = 0.00 : 0.00 : 1.00

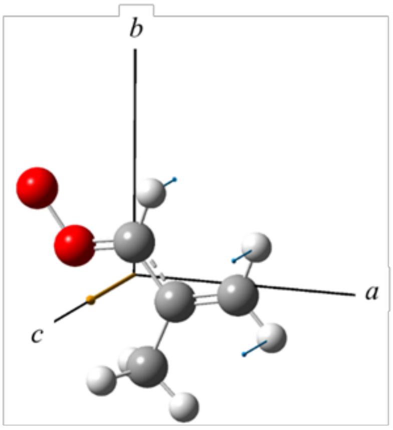

ν_{25}, 924 cm^{-1}

a : *b* : *c* = 0.00 : 0.00 : 1.00

Supplementary Fig. 9: Displacement vectors (blue arrows) and directions of dipole derivatives (yellow arrows) for modes ν_{14}, ν_{15}, ν_{24}, and ν_{25} of *anti-trans*-MACRO (3a) predicted with the B3LYP/aug-cc-pVTZ method. Molecular rotational axes are represented with labels *a*, *b*, and *c*. *a* : *b* : *c* is the mixing ratio of bands of types *a*, *b*, and *c* in each transition.

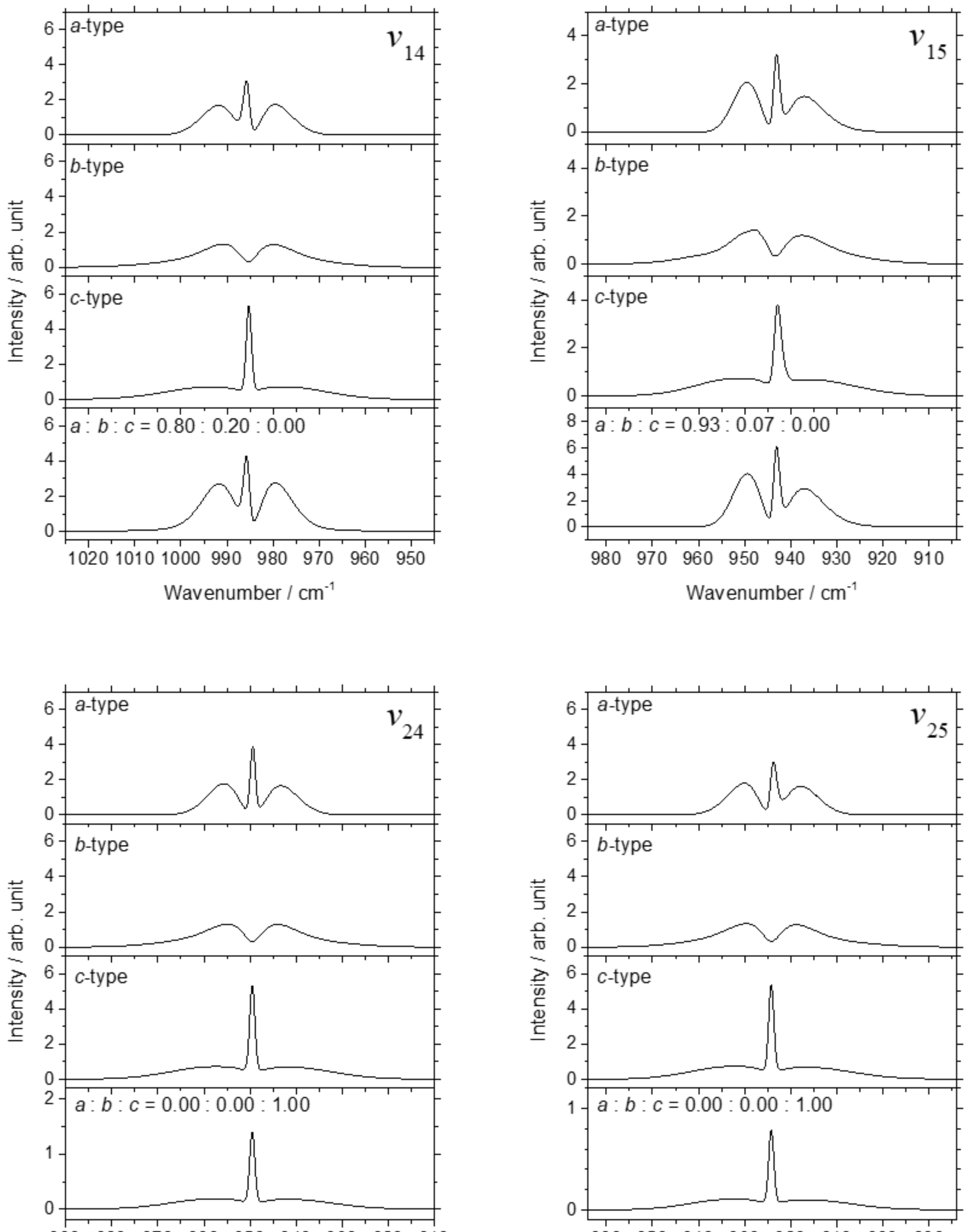

Supplementary Fig. 10: Rotational contours simulated for modes *v_{14}*, *v_{15}*, *v_{24}*, and *v_{25}* of *anti-trans*-MACRO (3a). Parameters used in the PGOPHER simulations are J_{max} = 150, T = 298 K, and Gaussian width (fwhm) = 1.28 cm^{-1}; type ratios are listed in figures.

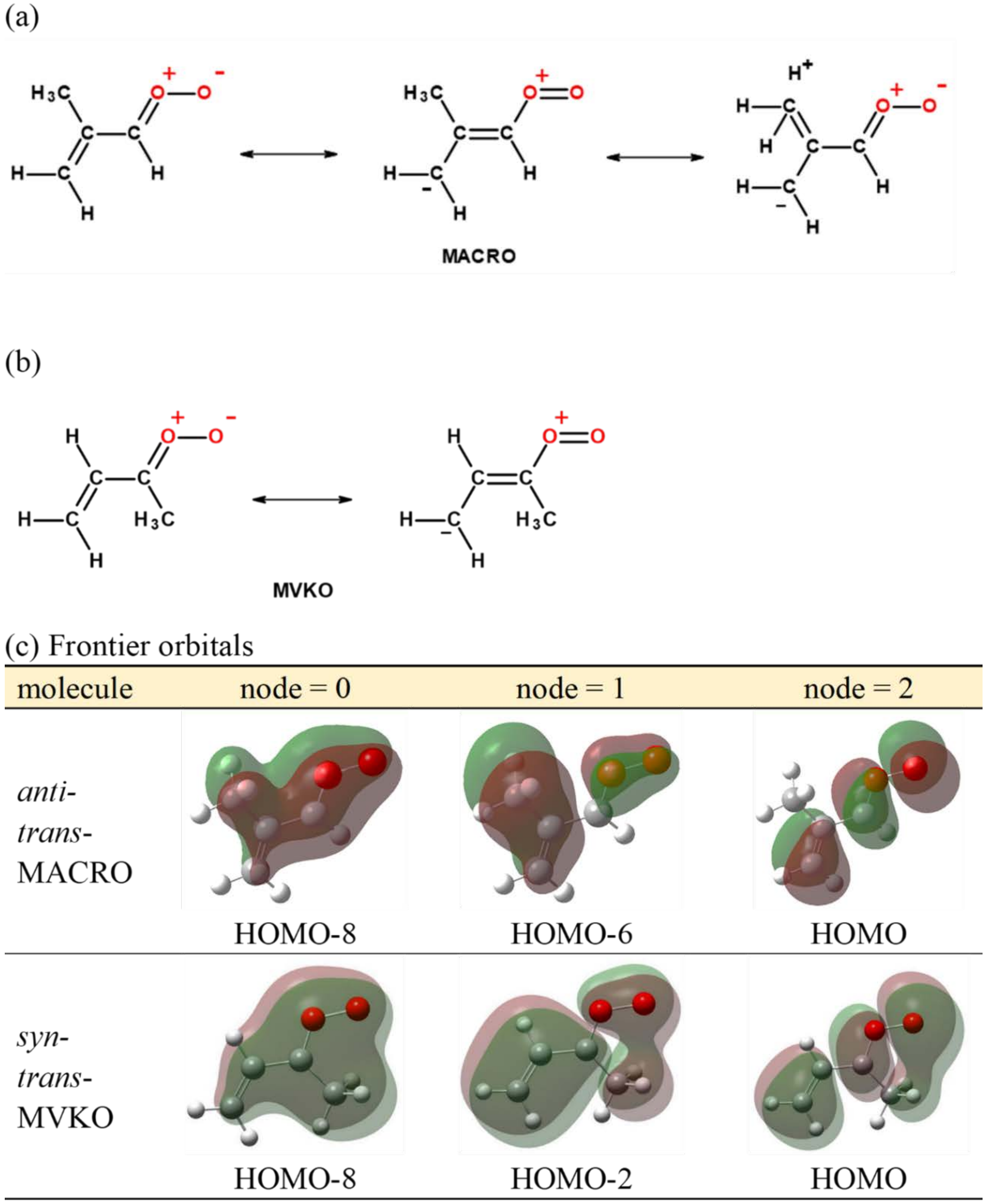

Supplementary Fig. 11: Resonance structures and frontier orbitals of *anti-trans*-MACRO (3a) and *syn-trans*-MVKO. Major resonance structures of *anti-trans*-MACRO **(3a)** and *syn-trans*-MVKO are shown in **a** and **b**, respectively; hyper-conjugation of **(3a)** is also shown in **a**. (c) Frontier orbitals of *anti-trans*-MACRO **(3a)** and *syn-trans*-MVKO showing delocalization over C–C–C–O–O.

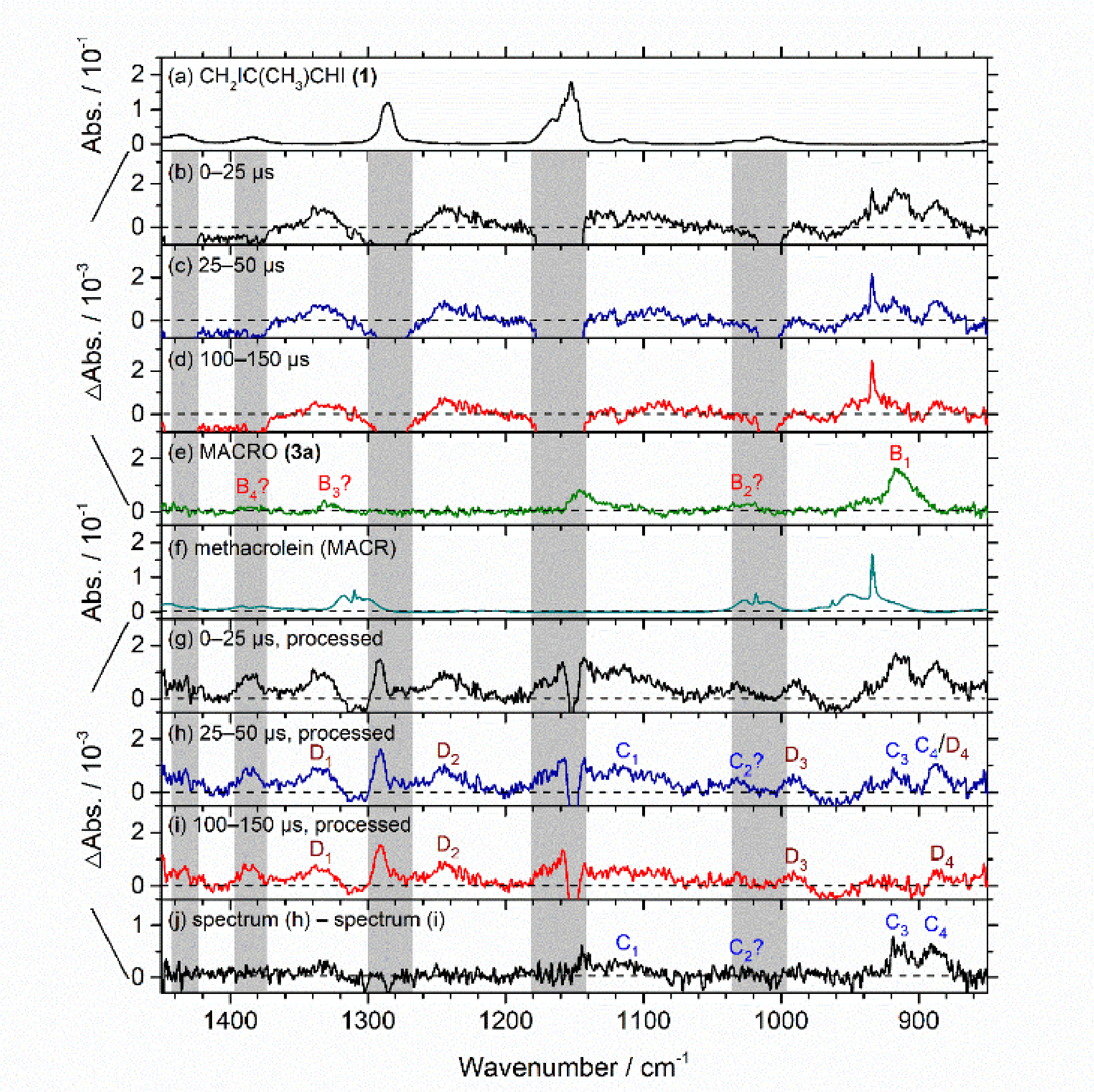

Supplementary Fig. 12: Observed and processed spectra in region 1450−850 cm^{-1} upon photolysis at 248 nm of a flowing mixture of $CH_2IC(CH_3)CHI$ (1)/O_2 (0.060/334 Torr). a Absorption spectrum before photolysis. Difference spectra recorded 0−25 (**b**), 25−50 (**c**), and 100−150 (**d**) μs after irradiation. **e** Spectrum of MACRO **(3)** taken from Supplementary Fig. 8h. **f** Absorption spectrum of methacrolein (MACR). **g**−**i** Processed spectra of **b**−**d** with bands of MACRO **(3a)** and MACR removed and those of the precursor **(1)** added back. **j** Spectrum **h** subtracts spectrum **i** to remove contribution of bands in group D. Grey areas represent regions of possible interference from absorption of the parent molecules **(1)**. New features are marked C_1−C_4 and D_1−D_4 in **h**−**j**. Spectral resolution is 1.0 cm^{-1}.

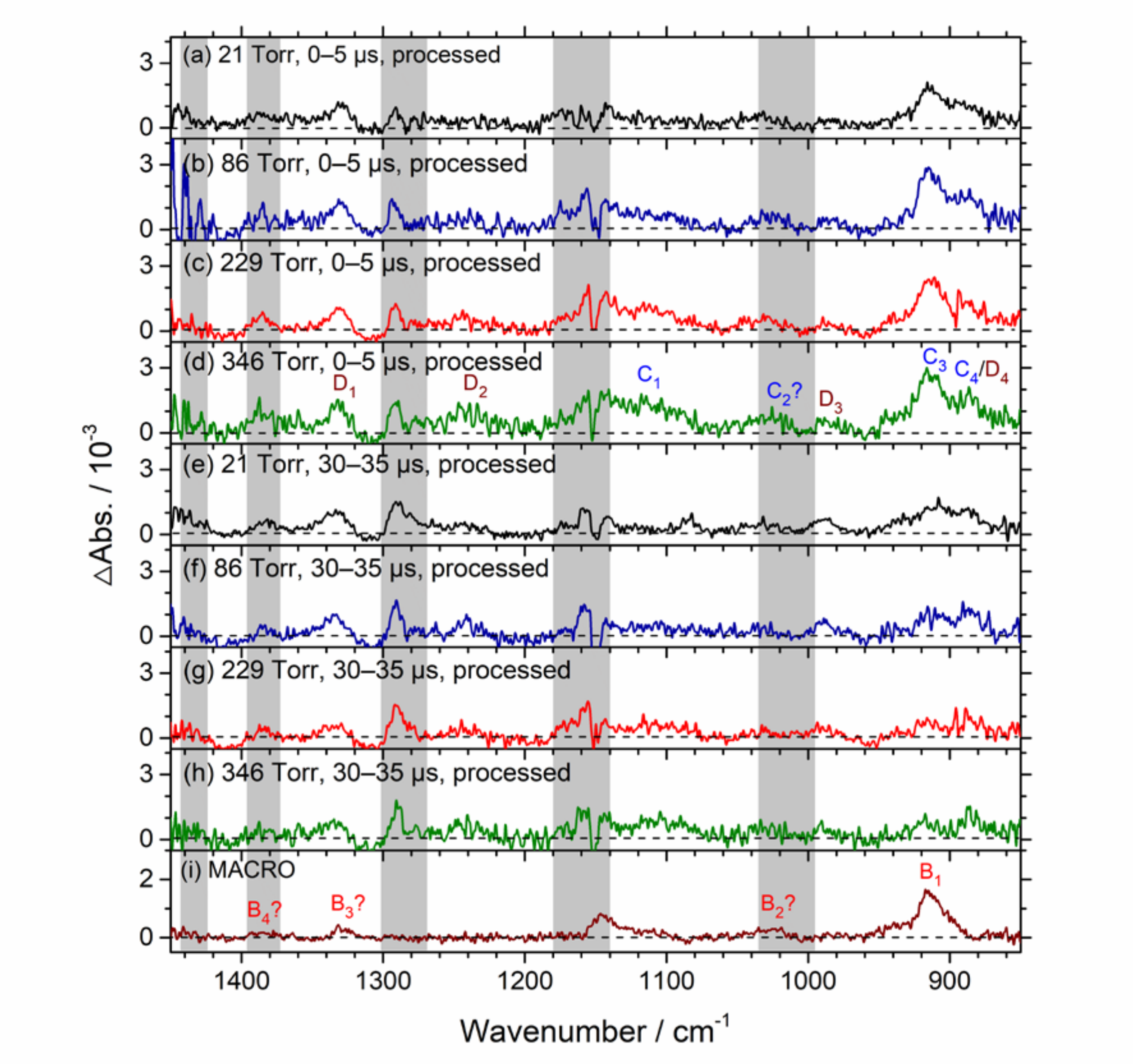

Supplementary Fig. 13: Comparison of processed spectra at various pressures recorded 0–5 and 30−35 μs in region 1450–850 cm^{-1} on photolysis at 248 nm of a flowing mixture of $CH_2IC(CH_3)CHI$ (1)/O_2 at 298 K. Processed spectra at (**a**) 21 Torr, (**b**) 86 Torr, (**c**) 229 Torr, and (**d**) 346 Torr recorded 0–5 μs after irradiation; bands of **(2)** and MACR were removed and those of the precursor **(1)** were added back. Grey areas represent regions of possible interference from absorption of the parent molecules. New features are marked C_1–C_4 and D_1–D_4 in **d**. **e**−**h** Processed spectra at **a** 21 Torr, **b** 86 Torr, **c** 229 Torr, and **d** 346 Torr recorded 30–35 μs after irradiation. **i** Spectrum of MACRO **(3)** taken from Supplementary Fig. 8h. Spectral resolution is 1.0 cm^{-1}.

Supplementary References

(1) Vansco, M. F. *et al*. Synthesis, electronic spectroscopy, and photochemistry of methacrolein oxide: a four-carbon unsaturated Criegee intermediate from isoprene ozonolysis. *J. Am. Chem. Soc.* **141**, 15058−15069 (2019).

(2) Western, C. M. *PGOPHER, A program for simulating rotational structure*, http://pgopher.chm.bris.ac.uk/.
